# Metabolic Reprogramming in Respiratory Viral Infections: A Focus on SARS-CoV-2, Influenza, and Respiratory Syncytial Virus

**DOI:** 10.3390/biom15071027

**Published:** 2025-07-16

**Authors:** Jordi Camps, Simona Iftimie, Andrea Jiménez-Franco, Antoni Castro, Jorge Joven

**Affiliations:** 1 Unitat de Recerca Biomèdica, Hospital Universitari de Sant Joan, Institut d’Investigació Sanitària Pere Virgili, Universitat Rovira i Virgili, Av. Dr. Josep Laporte 2, 43204 Reus, Catalonia, Spain; andrea.jimenez@urv.cat (A.J.-F.); jorge.joven@salutsantjoan.cat (J.J.); 2 Autoimmunity, Infection and Thrombosis Research Group (GRAIIT), Department of Internal Medicine, Hospital Universitari de Sant Joan, Institut d’Investigació Sanitària Pere Virgili, Universitat Rovira i Virgili, Av. Dr. Josep Laporte 2, 43204 Reus, Catalonia, Spain; simona.mihaela@salutsantjoan.cat (S.I.); antoni.castro@urv.cat (A.C.)

**Keywords:** infectious diseases, influenza, metabolism, respiratory infections, respiratory syncytial virus, severe acute respiratory syndrome coronavirus 2, viral infections

## Abstract

Respiratory infections caused by severe acute respiratory syndrome coronavirus 2, influenza virus, and respiratory syncytial virus pose significant global health challenges, leading to high morbidity and mortality, particularly in vulnerable populations. Despite their distinct virological characteristics, these viruses exploit host cellular metabolism to support replication, modulate immune responses, and promote disease progression. Emerging evidence shows that they induce metabolic reprogramming, shifting cellular energy production toward glycolysis to meet the bioenergetic demands of viral replication. Additionally, alterations in lipid metabolism, including enhanced fatty acid synthesis and disrupted cholesterol homeostasis, facilitate viral entry, replication, and immune evasion. The dysregulation of mitochondrial function and oxidative stress pathways also contributes to disease severity and long-term complications, such as persistent inflammation and immune exhaustion. Understanding these metabolic shifts is crucial for identifying new therapeutic targets and novel biomarkers for early disease detection, prognosis, and patient stratification. This review provides an overview of the metabolic alterations induced by severe acute respiratory syndrome coronavirus 2, influenza virus, and respiratory syncytial virus, highlighting shared and virus-specific mechanisms and potential therapeutic interventions.

## 1. Introduction

Respiratory viral infections remain a significant global health burden, contributing to substantial morbidity and mortality, particularly among vulnerable populations [1,2]. Among these, infections caused by severe acute respiratory syndrome coronavirus 2 (SARS-CoV-2), influenza virus, and respiratory syncytial virus (RSV) represent the most clinically relevant threats, leading to pandemics, seasonal outbreaks, and severe respiratory complications [3]. Despite their distinct virological characteristics, these viruses share a common strategy of hijacking host cellular metabolism to facilitate their replication, modulate immune responses, and drive pathogenesis.

Emerging evidence indicates that viral infections induce profound metabolic reprogramming, shifting cellular energy production, lipid metabolism, and amino acid utilization to favor viral propagation. SARS-CoV-2, influenza virus, and RSV all promote a metabolic switch toward glycolysis (resembling the Warburg effect observed in cancer cells) to meet the bioenergetic and biosynthetic demands of viral replication. Additionally, alterations in lipid metabolism, including increased fatty acid synthesis and disrupted cholesterol homeostasis, play a pivotal role in viral entry, replication, and immune evasion. Furthermore, the dysregulation of mitochondrial function and oxidative stress pathways contributes to disease severity and long-term complications, such as persistent inflammation and immune exhaustion [4,5,6].

Understanding these metabolic alterations is crucial for identifying novel therapeutic targets and optimizing treatment strategies. While current antiviral therapies primarily focus on direct inhibition of viral replication, targeting host metabolic pathways offers a promising complementary approach to limit viral spread and mitigate severe disease outcomes [7,8]. Moreover, investigating virus-induced metabolic reprogramming has the potential to reveal novel diagnostic and prognostic biomarkers, enabling earlier detection of disease progression and stratification of patients based on metabolic signatures [9]. This review aims to provide a comprehensive overview of the metabolic alterations induced by SARS-CoV-2, influenza virus, and RSV, highlighting shared and virus-specific mechanisms, their implications for disease pathogenesis, and potential metabolic interventions for future therapeutic development.

## 2. Lipid Metabolism

Viruses utilize lipid rafts for cell entry, manipulate fatty acid synthesis and β-oxidation to fuel replication, and disrupt cholesterol homeostasis, impairing the innate immune response. Lipid droplets also act as platforms for viral assembly and immune modulation. The interaction between viral infections and mitochondria is essential in lipid metabolism during infectious diseases. In catabolism, mitochondria are the primary site for lipid β-oxidation, where fatty acids are broken down into acetyl-CoA, which enters the Krebs cycle to generate adenosine triphosphate (ATP). In anabolism, mitochondria produce citrate, which is transported to the cytosol and converted into acetyl-CoA for fatty acid synthesis. Thus, mitochondria regulate the balance between lipid degradation and synthesis, adjusting metabolic processes in response to cellular energy demands during infection.

### 2.1. Role of Lipid Rafts in Viral Entry

**Figure 1:**
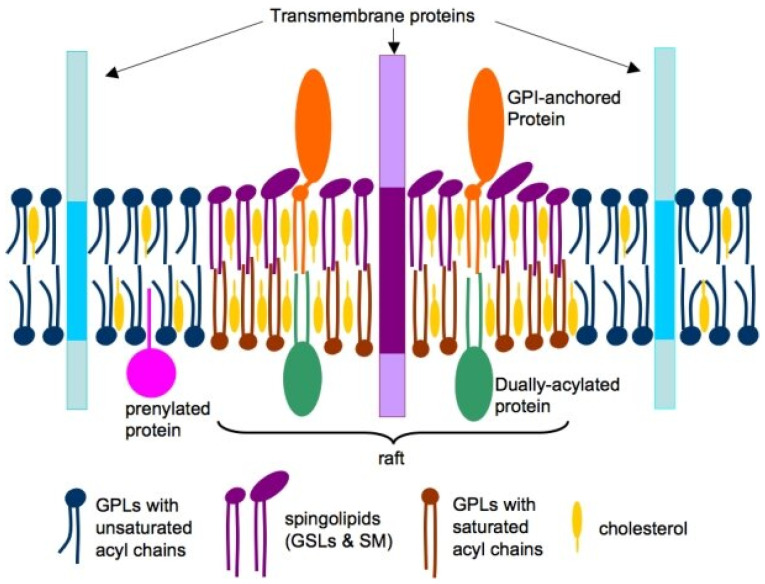
Conceptual illustration of lipid raft organization within cellular membranes. Cholesterol (yellow) and phospholipids (blue and brown) are present in both leaflets of the bilayer, while sphingolipids (violet) are predominantly localized in the outer leaflet. Lipids in raft regions typically feature long, saturated acyl chains (violet and brown), in contrast to the shorter and more unsaturated acyl chains (blue) found in non-raft areas. Raft microdomains are enriched in proteins with dual acylation (green) and glycosylphosphatidylinositol (GPI) anchors (brown), whereas transmembrane (blue) and prenylated (green) proteins are more commonly located in non-raft regions. GPLs: Glycerophospholipids; GSLs: Glycerosphingolipids; SM: Sphingomyelin. Reproduced from [11]. Copyright by the authors under a CC BY 4.0 license.

In animal cells, phospholipids comprise most of the lipids in cell membranes. However, the plasma membrane also incorporates glycolipids and cholesterol, essential for maintaining membrane fluidity [10]. While lipids typically move freely within the lipid bilayer, components such as cholesterol, glycosphingolipids, glycosylphosphatidylinositol (GPI)-anchored proteins, and some transmembrane proteins can cluster into specialized domains called lipid rafts [11,12]. The 2006 Keystone Symposium of Lipid Rafts and Cell Function defined these rafts as follows: “Small (10–200 nm), heterogenous, highly dynamic, sterol- and sphingolipid-enriched domains that compartmentalize cellular processes. Small rafts can sometimes be stabilized to form larger platforms through protein-protein and protein-lipid interactions” [13] (Figure 1).

Numerous viruses utilize these lipid rafts as key elements in the fusion process with the host cell. This complex process first requires the virus binding to the cell surface, often via a cell receptor. These interactions induce conformational changes that promote viral entry. Cholesterol, an essential component of rafts, is fundamental in stabilizing these complexes, promoting membrane fluidity, and facilitating fusion with the viral particles [14]. Before entry, many viruses establish weak ionic interactions with cell surface glycosaminoglycans, such as heparan and chondroitin sulfate, or with glycosphingolipids [15]. These interactions aid the virion’s adhesion to the membrane, allowing it to move across the cell surface until it finds the specific receptor needed for internalization [16]. Viral trafficking on the cell surface or within the endosomal network facilitates its arrival at the unmasking site while providing signals to activate its fusion machinery.

The location of viral receptors within lipid rafts affects the efficiency of the fusion process between the viral particle and the cell membrane. Some receptors are constitutively located in lipid rafts, while others are recruited to rafts after virus binding [17,18,19,20,21]. This dynamic highlights the complexity of the fusion process and underscores the regulatory role of lipid microdomains in infection. Different viruses may employ various routes to enter host cells, with some relying entirely on lipid rafts while others exploit alternative pathways. Raft-mediated entry mechanisms often involve the concentration of viral receptors within these domains, optimizing viral adhesion and fusion. In other cases, the virus attaches to regions outside the rafts before being directed to these structures to complete its entry [14,22,23,24].

In addition to direct fusion with the cell membrane [25,26,27], viruses may exploit endocytic pathways related to lipid rafts. Endocytosis is a cellular process that allows the internalization of substances from the extracellular environment. It can be divided into phagocytosis and pinocytosis. Phagocytosis is primarily employed by specialized cells, such as macrophages, to digest bacteria and large particles. Pinocytosis, conversely, is a more generalized, non-specific process for the uptake of liquids and macromolecules through endocytic vesicles [28]. Within this process, two primary mechanisms are distinguished: clathrin-mediated endocytosis and clathrin-independent endocytosis. Clathrin-mediated endocytosis [29] is the most widely studied pathway for internalizing small and medium-sized viruses. Virions, after moving laterally across the cell surface, are incorporated into clathrin-coated invaginations, which then detach from the plasma membrane to form endocytic vesicles. These vesicles lose their clathrin coat before fusing with endosomes. This mechanism depends on dynamin-II, a guanosine-5′-triphosphatase essential for separating clathrin-coated vesicles from the plasma membrane.

On the other side, clathrin-independent endocytosis [30,31,32] encompasses various cholesterol-sensitive pathways, including caveolae-mediated and other pathways that are neither clathrin- nor caveolae-dependent. Caveolae are specialized microdomains of lipid rafts that form invaginations in the plasma membrane. They are coated internally by caveolins, proteins that regulate cholesterol organization and caveola biogenesis. Additionally, cavins, which stabilize the caveola structure, play a role in membrane curvature and the formation of endocytic vesicles [33,34,35].

The central role of lipids in regulating these events has been recognized in the past decade. Although phosphatidylinositol (PI) is the least abundant phospholipid in the cell membrane, its signaling capacity is crucial for endosomal trafficking and maturation [10]. The differential phosphorylation of PI, regulated by specific kinases and phosphatases, generates various phosphorylated species that control cellular compartmentalization and associated signaling [36,37]. Many viruses have developed strategies to exploit PI-mediated signaling at different stages of infection, especially to coordinate their entry and reprogram the host cell.

The phosphoinositide 3-kinase (PI3K) pathway is one of the major signaling pathways activated during viral entry. The activation of PI3K and the production of PI triphosphate serve as anchoring platforms for proteins with lipid-binding domains, including Akt, a key regulator of the PI3K pathway [38]. This signaling pathway is involved in multiple forms of endocytosis, though its role has been best characterized in macropinocytosis, where it regulates cytoskeletal rearrangement and membrane dynamics during macropinosome formation [39]. Various viruses, such as influenza [40,41], activate PI3K signaling to facilitate their internalization. Additionally, some viruses use this pathway to regulate post-internalization events, such as viral replication and assembly.

Different viruses rely on lipid rafts to varying degrees. SARS-CoV-2 primarily enters cells through the binding of spike protein to the angiotensin-converting enzyme 2 (ACE2) receptor, a process facilitated by lipid rafts [42]. These cholesterol-rich microdomains stabilize the receptor and enhance viral fusion, and their disruption has been shown to reduce SARS-CoV-2 entry into epithelial cells [43], highlighting their importance in infection [44]. However, although lipid rafts are a major platform for SARS-CoV-2 entry, it has recently been reported that the virus can also bind to ACE2 outside of these microdomains and be internalized via fusion [45], suggesting that lipid rafts are not strictly required for viral entry. The influenza virus utilizes multiple entry mechanisms, including clathrin-mediated endocytosis and macropinocytosis [44,46,47], which may or may not involve lipid rafts. However, it has been suggested that rafts are attachment points, aiding multivalent binding and increasing infection efficiency [48]. While lipid rafts play a role in influenza virus attachment, their membrane fusion necessity is less pronounced than SARS-CoV-2. RSV has a less well-defined relationship with lipid rafts. While certain studies suggest that its fusion proteins are associated with raft-like domains [49], others indicate that infection can occur independently of these structures [50].

In summary, viruses may employ lipid rafts as platforms for viral attachment, fusion, and entry. Many viruses, including SARS-CoV-2, influenza, and RSV, exploit these microdomains to enhance infection efficiency. However, the degree of dependence on lipid rafts varies among viruses. While SARS-CoV-2 primarily utilizes these structures for entry, influenza virus and RSV exhibit greater flexibility, often engaging alternative pathways to facilitate infection. Beyond their role in viral entry, lipid metabolism influences downstream processes critical to viral replication and assembly. In particular, many viruses fine-tune their infection cycle by hijacking phosphoinositide-mediated pathways, ensuring efficient propagation within the host. 

### 2.2. Fatty Acid Synthesis, Lipid Droplets, and β-Oxidation Dysregulation 

**Figure 2:**
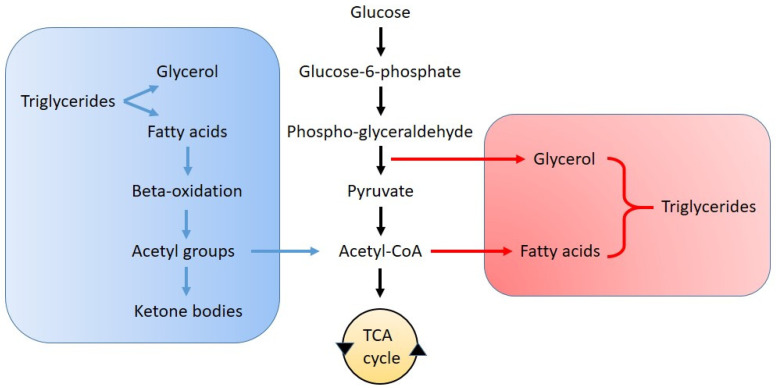
Schematic representation of the pathways of lipolysis (blue) and lipogenesis (red) in relation to glycolysis and the tricarboxylic acid (TCA) cycle.

The relationship between lipid synthesis and glycolysis is schematized in Figure 2. Fatty acid synthesis is a highly regulated anabolic pathway that converts acetyl-CoA into long-chain fatty acids and usually occurs in the liver and adipose tissues. This process is primarily controlled by acetyl-CoA carboxylase, which converts acetyl-CoA to malonyl-CoA, and fatty acid synthase (FASN), which catalyzes the sequential elongation of fatty acid chains. The sterol regulatory element-binding proteins (SREBPs), particularly SREBP-1c, play a key role in regulating the expression of these enzymes in response to metabolic cues. Additionally, the activity of ATP-citrate lyase, which provides cytosolic acetyl-CoA, and elongases that extend fatty acid chains further modulate lipid biosynthesis [51].

Viruses exploit fatty acid synthesis to support their replication. SARS-CoV-2 [52,53], influenza [54], and RSV [55] upregulate FASN expression to promote lipid biosynthesis. Increased fatty acid synthesis provides a lipid-rich environment for forming viral replication complexes and membranous vesicles that serve as viral replication platforms. Pharmacological inhibition of FASN has been shown to reduce viral replication in multiple models, highlighting its potential as an antiviral target [56].

Lipid droplets (LDs) are dynamic organelles of a neutral lipid core surrounded by a phospholipid monolayer. They store triacylglycerols and cholesterol esters, which can be mobilized through lipolysis under metabolic stress or increased cellular demand [57]. LD formation is regulated by diacylglycerol O-acyltransferase 1 and 2 (DGAT1 and DGAT2), which mediate triacylglycerol synthesis and packaging within the endoplasmic reticulum membrane [58]. The release of LD into the cytoplasm is influenced by SREBP-1 and perilipin, a droplet membrane protein [59].

SARS-CoV-2, influenza A, and RSV manipulate LD within host cells through distinct mechanisms to enhance their replication. SARS-CoV-2 promotes their synthesis, as evidenced by increased LD accumulation in monocytes from coronavirus disease (COVID-19) patients compared to uninfected individuals. In vitro studies have shown that SARS-CoV-2 modulates SREBP-1 and DGAT-1 expression, triggering LD formation [60]. In contrast, influenza A virus activates the mechanistic target of rapamycin (mTOR) complexes 1 and 2, inducing autophagy and LD degradation to support viral replication [61,62,63]. Although RSV’s interaction with LD is less understood, animal studies indicate that they disperse and degrade LD, contributing to oxidative stress, inflammation, and airway hyperresponsiveness [64].

Fatty acid oxidation (β-oxidation) breaks down fatty acids into acetyl-CoA for ATP production in mitochondria and peroxisomes and is regulated by carnitine palmitoyltransferases (CPT) [65,66]. Because β-oxidation is a significant energy source in times of high demand, viruses have evolved mechanisms to manipulate this pathway to optimize their replication, either by downregulating it to accumulate free fatty acids for membrane synthesis or upregulating it to boost ATP production [67,68]. For instance, SARS-CoV-2 has been shown to impair fatty acid oxidation in alveolar epithelial cells, leading to decreased expression of CPT1A and peroxisome proliferator-activated receptor-γ coactivator 1- α (PGC-1α), both critical for mitochondrial function [69]. Influenza virus can suppress β-oxidation by downregulating carnitine palmitoyltransferase II (CPT II) and altering mitochondrial dynamics, contributing to metabolic reprogramming in infected cells [70]. RSV infection has also been linked to metabolic dysfunction, including disruptions in mitochondrial fatty acid oxidation [71], though evidence remains limited and requires further confirmation.

### 2.3. Disrupted Cholesterol Homeostasis and Innate Immune Evasion

Cholesterol is essential to eukaryotic cell membranes, ensuring structural integrity, regulating membrane fluidity, and facilitating key cellular functions such as endocytosis, vesicular transport, and immune response modulation. Cellular cholesterol homeostasis is tightly controlled through endogenous synthesis, receptor-mediated uptake of low-density lipoproteins (LDLs), and high-density lipoprotein (HDL)-dependent efflux, governed by SREBP and liver X receptors [72].

HDL particles are considered to be a part of the innate immune system. Under normal conditions, HDLs exhibit anti-inflammatory, antioxidant, and protective properties [73]. They facilitate reverse cholesterol transport, prevent lipid oxidation, and modulate immune cell activity, thereby reducing oxidative stress and inflammation. Additionally, HDLs help neutralize bacterial toxins and support defense mechanisms [74].

**Figure 3:**
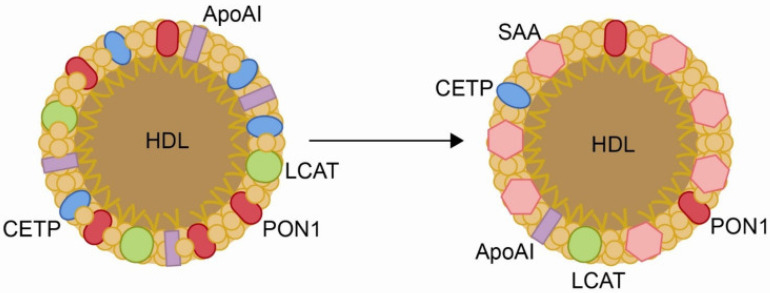
Changes in the structure of high-density lipoproteins (HDLs) produced by inflammation. Chronic inflammatory processes cause a decrease in the content of paraoxnase-1 (PON1), apolipoprotein AI (Apo AI), lecithin/cholesterol acyltransferase (LCAT), and cholesterol ester transfer protein (CETP), and an increase in the concentration of serum amyloid A (SAA). Reproduced from [77]. Copyright by the authors under a CC BY 4.0 license.

However, during infection and inflammation, the host initiates a cascade of responses known as the acute-phase response, which profoundly disrupts lipid metabolism, particularly HDL function. During the acute-phase response, the levels of key proteins involved in HDL-mediated reverse cholesterol transport decline, including lecithin/cholesterol acyltransferase, cholesterol ester transfer protein, phospholipid transfer protein, apolipoprotein A-I, and paraoxonase 1 [75]. These alterations impair HDL’s ability to remove excess cholesterol and neutralize oxidative damage. Simultaneously, HDL composition shifts, characterized by cholesterol ester depletion and enrichment in free cholesterol, triglycerides, and free fatty acids. Furthermore, apolipoprotein J and serum amyloid A levels increase substantially, replacing apolipoprotein A-I and transforming HDL into a pro-atherogenic and pro-inflammatory particle [76,77]. As a result, HDL loses its protective functions, instead promoting endothelial dysfunction, oxidative stress, and chronic inflammation, which may contribute to immune evasion by pathogens (Figure 3).

In addition to alterations in HDL composition, infectious diseases induce changes in other lipoproteins. The enhanced secretion of very low-density lipoproteins (VLDLs) is attributed to increased lipolysis in adipose tissue, augmented hepatic fatty acid synthesis, and suppressed fatty acid oxidation. Impaired lipoprotein lipase and apolipoprotein E activity further contribute to reduced VLDL clearance. LDLs are also affected, with their levels varying depending on the type and severity of infection. In some viral infections, LDL concentrations decrease due to increased catabolism or reduced hepatic synthesis, whereas in others, LDL oxidation contributes to foam cell formation, exacerbating inflammation and atherosclerosis. These disruptions in cholesterol homeostasis not only impair lipid transport but may also facilitate immune evasion by pathogens, highlighting the intricate interplay between lipid metabolism and host defense mechanisms [75,77,78].

Recent studies have indicated that COVID-19 patients have larger and more abundant VLDL particles than healthy individuals, along with elevated VLDL-cholesterol and VLDL–triglyceride concentrations. In contrast, LDL–cholesterol concentrations were lower, with LDL particles being larger and fewer in number. HDL particles exhibited reduced cholesterol content and increased triglycerides, accompanied by a notable decrease in small HDL particles [79,80,81,82,83,84].

Similar lipoprotein alterations have been observed in influenza, though the pattern varies. HDL cholesterol levels are often reduced in this disease, while LDL cholesterol levels tend to increase during the acute phase [85]. However, these changes are generally less pronounced than in SARS-CoV-2 infection and do not appear to be major drivers of disease progression. The exception is low HDL levels at hospital admission, which have been associated with higher inflammation and increased mortality, particularly in males [86]. 

Information on lipoprotein alterations in RSV infection is scarce. In vitro studies [87] suggested that RSV infection activates the SREBP2 and low-density lipoprotein receptor (SREBP2-LDLR) pathway, which is a key regulatory mechanism in cholesterol uptake. Activating this pathway increases the expression of LDL receptors and promotes cholesterol uptake into cells, contributing to cholesterol accumulation in lysosomes, which supports viral replication. This mechanism suggests that lipoprotein changes during RSV infection may involve increased LDL uptake, although specific alterations in lipoprotein profile (such as changes in HDL or LDL levels) have not yet been characterized.

Respiratory virus infections not only alter circulating lipoprotein concentrations but also modify intracellular cholesterol metabolism to enhance viral replication and release. A recent preprint reported that SARS-CoV-2 disrupts host lipid metabolism by inducing lysosomal cholesterol sequestration, a process facilitated by the interaction between the viral protein ORF3a and the host protein VPS39, which impairs cholesterol trafficking and contributes to viral pathogenesis [88]. In contrast, influenza A virus exploits cholesterol through distinct mechanisms, primarily relying on cholesterol-rich lipid rafts to facilitate receptor clustering and endocytosis via its hemagglutinin glycoprotein. Disrupting these lipid rafts with cholesterol-depleting agents, such as methyl-β-cyclodextrin, impairs viral entry and reduces infectivity [89]. This virus primarily manipulates intracellular cholesterol to promote viral ribonucleoprotein export, virion assembly at the plasma membrane, and efficient budding. Cholesterol-rich microdomains are essential for releasing infectious virions, and cholesterol depletion has been shown to result in malformed particles with diminished infectivity [90]. Unlike SARS-CoV-2 and influenza A virus, RSV does not appear to induce widespread systemic cholesterol dysregulation. However, cholesterol depletion in host cells has been shown to impair RSV fusion and reduce viral replication [87], highlighting its dependence on lipid rafts. Additionally, RSV infection seems to modulate cholesterol metabolism to support viral assembly and budding, although the exact molecular mechanisms remain less well characterized than other respiratory viruses [50].

Alterations of respiratory virus infections on the host lipid metabolism are summarized in Table 1.

## 3. Energy Metabolism and Mitochondrial Dynamics

The metabolic response to viral infections is characterized by alterations in energy demand and mitochondrial function [71]. Cellular energy metabolism is primarily driven by three interconnected pathways: glycolysis, the Krebs cycle, and oxidative phosphorylation (OXPHOS). Glycolysis occurs in the cytosol, breaking down glucose into pyruvate while generating ATP and reduced nicotinamide adenine dinucleotide (NADH). Under aerobic conditions, pyruvate is transported into mitochondria, where it enters the Krebs cycle, producing NADH and flavin adenine dinucleotide, which fuel the electron transport chain for ATP synthesis via OXPHOS. The balance between these metabolic pathways is crucial for cellular homeostasis, immune responses, and host defense mechanisms against viral infections [91].

Mitochondria play a central role in cellular bioenergetics, integrating energy production with immune signaling. Viral infections frequently disrupt mitochondrial function, leading to altered ATP generation, shifts in metabolic fluxes, and modulation of host defense mechanisms [71,92].

### 3.1. Glycolysis and the Warburg-like Effect in Viral Infections

**Figure 4:**
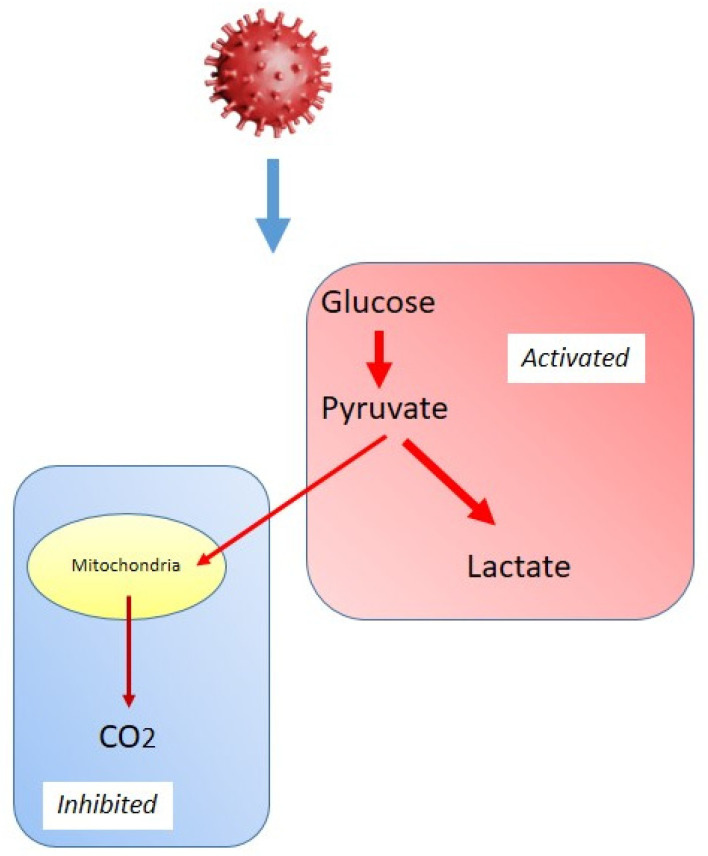
Schematic representation of the “Warburg-like effect” in viral infections.

Viruses extensively reprogram host cell metabolism to facilitate their replication and persistence. One of the most well-documented metabolic alterations induced by viral infections is the shift towards increased glycolysis, a phenomenon reminiscent of the Warburg effect observed in cancer cells (Figure 4). This metabolic adaptation, often called a “Warburg-like effect”, is characterized by enhanced glycolytic flux despite oxygen availability, leading to reduced mitochondrial respiration [93,94]. This strategy provides infected cells with a rapid supply of ATP and metabolic intermediates essential for viral genome replication, protein synthesis, and lipid membrane production. The Warburg-like effect in viral infections is driven by multiple host and viral factors, including the activation of hypoxia-inducible factor 1-α (HIF-1α), OXPHOS suppression, and modulation of key metabolic enzymes [95,96]. Respiratory viruses exhibit profound metabolic reprogramming that favors glycolysis over mitochondrial respiration.

One of the key mechanisms by which SARS-CoV-2 enhances glycolysis is through the upregulation of enzymes, including hexokinase 2, phosphofructokinase, and lactate dehydrogenase [97]. This metabolic shift ensures a continuous supply of ATP and biosynthetic precursors, sustaining viral replication and assembly. Moreover, SARS-CoV-2 has been shown to suppress OXPHOS by disrupting mitochondrial function, leading to metabolic dependence on glycolysis [98]. In addition to direct metabolic reprogramming, inflammatory responses associated with COVID-19 further reinforce glycolytic metabolism. Infected immune cells, particularly monocytes and macrophages, exhibit an increased glycolytic phenotype, contributing to a hyperinflammatory state [96]. This excessive metabolic shift may exacerbate disease severity, promoting lung tissue damage and cytokine storm.

Influenza virus reprograms host cell metabolism by promoting a shift toward glycolysis, primarily through the activation of HIF-1α, a key regulator of cellular metabolism under hypoxic conditions [99,100]. During infection, HIF-1α activation has been shown to upregulate glucose transporters (GLUT1, GLUT3) and key glycolytic enzymes, enhancing glucose uptake and metabolic flux [101,102]. This metabolic adaptation ensures a rapid supply of energy and biosynthetic intermediates essential for viral protein synthesis and replication while modulating the host immune response. However, excessive glycolytic activity may impair antiviral defense mechanisms, increasing host cell susceptibility to severe infection. In addition to enhancing glycolysis, influenza virus disrupts mitochondrial function by interfering with mitochondrial dynamics. Specifically, it has been reported to induce mitochondrial fission, resulting in fragmented mitochondria with diminished OXPHOS efficiency. This mitochondrial disruption further reinforces the reliance on glycolysis, creating a metabolic environment that facilitates viral replication and propagation [103].

RSV is another important pathogen known to rewire host cell metabolism towards glycolysis. Like SARS-CoV-2 and influenza, this virus enhances glycolytic flux to sustain viral replication. RSV infection has been shown to activate the insulin receptor-PI3K-Akt axis, upregulate the translation and activity of HIF-1α, increase the expression of GLUT1, GLUT3, and GLUT4, hexokinase 1 and 2, and platelet-type phosphofructokinase, and promote glucose uptake and glycolysis. In addition, mitochondrial damage induced by RSV resulted in the generation of large amounts of reactive oxygen species (ROS) in infected cells, which contributed to stabilizing HIF-1α [104]. 

The shift towards glycolysis in virally infected cells has profound implications for the host immune response. Activated immune cells, including macrophages, dendritic cells, and T cells, rely on glycolysis for rapid energy production and effector function. However, excessive metabolic reprogramming can harm immune function and disease progression. SARS-CoV-2-infected macrophages exhibit a hyperglycolytic state, leading to increased production of pro-inflammatory cytokines such as interleukin (IL)-6, tumor necrosis factor-α (TNF-α), and IL-1β [96]. This effect contributes to the hyperinflammatory response observed in severe COVID-19 cases. T cell activation depends on glycolysis, but persistent viral infections can lead to metabolic exhaustion, reducing T cell proliferation and cytotoxic function [105]. Metabolic reprogramming in dendritic cells affects their ability to present antigens and stimulate adaptive immune responses, potentially impairing viral clearance [106].

The Warburg-like effect in viral infections highlights the intricate relationship between host metabolism and viral pathogenesis. By shifting cellular energy production towards glycolysis while suppressing mitochondrial function, viruses create an environment conducive to their replication. This metabolic reprogramming sustains viral growth and impacts immune function, influencing disease severity and outcomes. Understanding these metabolic alterations provides new insights into antiviral strategies that target host cell metabolism, potentially leading to novel therapeutic interventions for respiratory viral infections.

### 3.2. Mitochondrial Dysfunction and Bioenergetic Failure

Mitochondria are essential organelles that serve as the cell’s powerhouse and key regulators of innate immunity. Their ability to generate ATP through OXPHOS is crucial for cellular homeostasis, while their role in immune signaling makes them a primary target for viral manipulation. Numerous viruses have evolved sophisticated mechanisms to disrupt mitochondrial function [107], leading to bioenergetic failure and immune evasion. This mitochondrial dysfunction contributes to viral persistence, increased pathogenicity, and exacerbation of disease severity [108].

One of the major consequences of viral infections on mitochondrial function is the suppression of OXPHOS, which leads to decreased ATP production [109]. Many viruses disrupt electron transport chain activity, impairing efficient ATP synthesis [110]. For example, research indicates that SARS-CoV-2 can suppress the expression of both nuclear-encoded and mitochondrial-encoded mitochondrial genes, impairing mitochondrial function. This downregulation affects electron transport chain components, including complexes I and III, thereby disrupting oxidative phosphorylation and ATP production [111,112]. 

Mitochondria are highly dynamic organelles that continuously undergo fission (division) and fusion (joining) to maintain their function and adapt to cellular demands [113]. Viral infections often hijack these processes to optimize viral replication and evade host immune responses. Fission is primarily mediated by dynamin-related protein 1 (Drp1), which translocates to the mitochondria upon activation, causing mitochondrial fragmentation [114]. SARS-CoV-2, influenza, and Dengue exploit Drp1-mediated fission to disrupt mitochondrial homeostasis [115]. SARS-CoV-2 has been shown to upregulate Drp1 and increase mitochondrial fission, decreasing ATP production in cultured cells [116]. This mitochondrial fragmentation also impairs immune cell function, dampening antiviral responses and facilitating viral persistence. Moreover, lymphocytes from patients recovered from severe COVID-19 were reported to have high Drp1 expression, a disruption in mitochondrial dynamics, as well as a lack of structural integrity in the electron transport chain, and altered circulating levels of IL-1β, interferon (IFN)-α2, and IL-27 [117]. Conversely, fusion is controlled by mitofusins 1 and 2 and optic atrophy 1, which help maintain mitochondrial integrity and bioenergetic efficiency [118]. Some viruses actively suppress fusion to hinder mitochondrial repair and prolong cellular dysfunction. However, the influenza virus has been reported to exert a dual effect in cultured cells, inducing mitochondrial fragmentation under serum starvation while promoting fusion and mitochondrial elongation under optimal culture conditions [119].

A crucial component of mitochondrial antiviral defense is the mitochondrial antiviral-signaling protein (MAVS) [120]. MAVS is localized on the outer mitochondrial membrane and acts as a signaling hub for type I IFN responses upon viral detection. Respiratory viruses have evolved strategies to inhibit MAVS function [121]. SARS-CoV-2 protein ORF degrades MAVS, thereby blocking downstream IFN signaling and allowing the virus to evade immune detection [122]. In addition to MAVS, other mitochondrial proteins play roles in antiviral defense. The NOD-, LRR-, and pyrin domain-containing protein 3 (NLRP3) inflammasome, a key component of innate immunity, is activated in response to mitochondrial stress [123]. Studies in cultured cells showed that SARS-CoV-2 and influenza virus exploit mitochondrial damage to overactivate NLRP3. Instead of effectively fighting the infection, excessive NLRP3 activation triggers uncontrolled inflammation, which can be harmful to the host and may even lead to organ failure and death [98,124,125].

Mitochondria possess quality control mechanisms, such as mitophagy (mitochondrial autophagy), to eliminate damaged mitochondria and maintain cellular homeostasis. Some viruses hijack these pathways to their advantage. For example, the Zika virus activates mitophagy in cultured trophoblasts to degrade mitochondria, suppressing immune responses and prolonging infection [126]. Conversely, SARS-CoV-2 has been reported to impair mitophagy, accumulating dysfunctional mitochondria and excessive inflammation. The inhibition of mitophagy can result in prolonged mitochondrial dysfunction, contributing to disease pathology and immune dysregulation [127].

Mitochondrial dysfunction is a common feature of many viral infections, contributing to metabolic reprogramming, immune evasion, and disease progression. By suppressing OXPHOS, promoting mitochondrial fragmentation, disrupting MAVS signaling, and manipulating mitochondrial quality control, many viruses, including SARS-CoV-2, IV, and RSV, create an environment conducive to favoring their replication and impairing the host defense mechanisms.

### 3.3. Cellular Metabolic Sensors and Viral Manipulation

**Figure 5:**
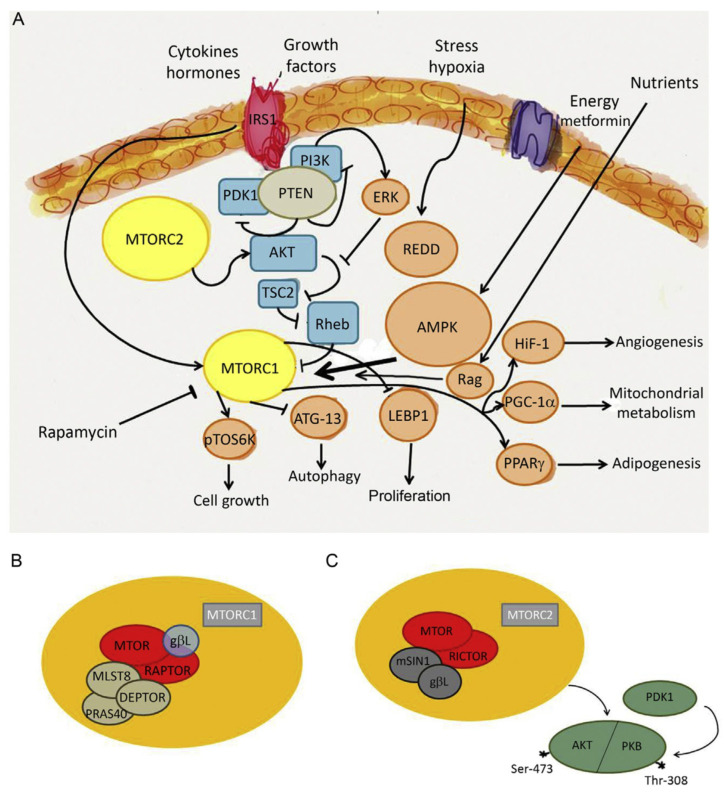
The mechanistic target of rapamycin (MTOR) belongs to the phosphatidylinositol 3-kinase-related kinase protein family. It is a serine/threonine protein kinase that regulates autophagy, cell growth, cell proliferation, cell motility, cell survival, protein synthesis, and transcription. MTOR integrates the input from upstream pathways that are involved in noncommunicable diseases, including insulin, growth factors, and the status of cellular nutrient, oxygen, and energy. Of note, other pathways unrelated to energy metabolism may also be activated. For instance, recombinationactivating gene (RAG) protein is a substrate of AMPK (**A**). MTOR is the catalytic subunit of two molecular complexes: MTORC1 and MTORC2. The role of MTORC1 is to activate translation of proteins and is composed of MTOR, regulatory-associated protein of MTOR (Raptor), mammalian lethal with SEC13 protein 8 (MLST8), and other partners such as PRAS40 and DEPTOR (**B**). MTORC2 functions as a regulator of the cytoskeleton and phosphorylates the serine/threonine protein kinase Akt/PKB at the serine residue S473. Phosphorylation of the serine stimulates Akt phosphorylation at the threonine Thr-308 residue via PDK1 and leads to full Akt activation. MTORC2 is composed of MTOR, rapamycin-insensitive companion of MTOR (RICTOR), GbL, and mammalian stress-activated protein kinase-interacting protein 1 (mSIN1) (**C**). Other abbreviations: ATG-13, autophagy-related protein 13; ERK, extracellular signal-regulated kinase; HIF-1, hypoxia-inducible factor 1; IRS, insulin receptor substrate; LEBP, lung epithelial binding peptide; PGC-1a, peroxisome proliferators-activated receptor g coactivator-1a; PI3K, phosphatidylinositol 3-kinase; PPARg, peroxisome proliferators-activated receptor g; PTEN, phosphatase and tensin homolog deleted on chromosome TEN; pTOS6k, p70 ribosomal S6 kinase; Rag, Ras-related GTPase; REDD, regulated in development and DNA damage responses; Rheb, Ras homolog enriched in brain; TSC2, tuberous sclerosis protein 2. Reproduced from [129] with permission. Copyright by Elsevier.

As discussed in the previous sections, mitochondrial dysfunction and the reprogramming of energy metabolism are hallmarks of viral infections, driving a shift from OXPHOS to glycolysis to support viral replication. Beyond these mitochondrial alterations, viruses also target key cellular metabolic sensors, including AMP-activated protein kinase (AMPK) and mTOR, to further manipulate host cell metabolism in their favor. These pathways are critical in determining whether a cell enters a catabolic or anabolic state, thereby influencing viral replication and immune responses [128,129] (Figure 5).

AMPK serves as a central energy sensor that becomes activated under conditions of energy stress, such as viral infection. When ATP levels drop, AMPK is phosphorylated and activated to restore energy homeostasis by promoting catabolic pathways, including fatty acid oxidation and autophagy, while simultaneously inhibiting anabolic processes such as protein and lipid synthesis [130]. By reducing cellular biosynthetic activity, AMPK activation creates a less favorable environment for viral replication, as many viruses rely on an abundance of host-derived macromolecules. Consequently, some viruses have evolved mechanisms to inhibit AMPK signaling to prevent host cells from entering an energy-saving state. For example, hepatitis B and C viruses have been reported to suppress AMPK activity to ensure sufficient lipid availability for viral envelope formation [129,131]. In contrast, certain viruses may activate AMPK to promote selective autophagy (mitophagy), removing damaged mitochondria that could trigger antiviral immune responses [132]. RSV, for instance, has been shown to induce mitophagy in infected cells, which may contribute to immune evasion by reducing mitochondrial ROS production and dampening innate immune activation [133].

While AMPK functions as a metabolic checkpoint that limits viral replication, mTOR plays an opposing role by promoting anabolic metabolism and cellular growth [129]. mTOR is a key regulator of protein and lipid synthesis, which viruses often hijack to facilitate their replication cycle. Many viruses, including the influenza virus, RSV, hepatitis C virus, and human cytomegalovirus, activate mTOR signaling to enhance the production of viral proteins and structural components [134,135]. The mTOR pathway is particularly important for regulating translation through its downstream effectors, such as ribosomal protein S6 kinase and eukaryotic initiation factor 4E-binding protein 1, which govern ribosome biogenesis and mRNA translation [136]. Additionally, mTOR activation can suppress autophagy and autophagy-induced catabolism by phosphorylating unc-51-like kinase 1, autophagy-related gene 13, and other molecules [137]. Since autophagy can degrade viral components, some viruses actively evade this process. Conversely, in certain scenarios, viruses may also exploit autophagy to generate intracellular membranes necessary for viral replication compartments, as observed in SARS-CoV-2 infection [138].

Another key metabolic signaling pathway frequently manipulated by viruses is the PI3K-Akt pathway. This pathway is a major upstream activator of mTOR, promoting cell growth and survival by integrating signals from growth factors and nutrients. Activation of PI3K leads to Akt phosphorylation, which activates mTOR complex 1, thereby regulating processes such as protein synthesis and autophagy suppression [139]. PI3K-Akt signaling plays a central role in cell survival, proliferation, and glucose metabolism, making it an attractive target for viral interference. Activation of this pathway promotes glycolysis by upregulating glucose transporters and key glycolytic enzymes, ensuring a continuous supply of biosynthetic precursors required for viral assembly [137,138,139,140]. Influenza virus, RSV, and SARS-CoV-2 have been reported to modulate the PI3K-Akt pathway to create a metabolic environment conducive to replication [140]. This pro-survival signaling facilitates sustained viral replication and contributes to immune evasion by preventing premature cell death and subsequent antigen presentation.

The interplay between AMPK, mTOR, and PI3K-Akt highlights the intricate metabolic rewiring during viral infections. While AMPK activation generally acts as a barrier to viral replication by inducing catabolic pathways and inhibiting biosynthesis, mTOR and PI3K-Akt activation facilitate viral propagation by driving anabolic metabolism and cell survival. However, the context-dependent effects of these metabolic regulators must be carefully considered, as their inhibition could also impact immune cell function and host defense mechanisms.

Overall, viruses have evolved sophisticated strategies to hijack metabolic signaling pathways to optimize their replication and evade immune responses. Understanding the interplay between AMPK, mTOR, and PI3K-Akt in the context of viral infections provides valuable insights into host–virus interactions and potential therapeutic targets to restore metabolic balance and enhance antiviral immunity.

Table 2 provides a comparative overview of the main characteristics of how SARS-CoV-2, influenza virus, and RSV affect glycolysis, mitochondrial function, and cellular metabolic sensors in host cells.

## 4. Metabolic Alterations in Amino Acid and Nucleotide Pathways

Respiratory viral infections induce profound disruptions in amino acid and nucleotide metabolism, which are crucial in disease progression and influence viral replication, immune function, and cellular stress responses. Understanding these metabolic shifts provides insight into the host–pathogen interaction and the consequences of viral infections on cellular homeostasis.

### 4.1. Amino Acid Metabolism

Glutamine is a critical amino acid involved in energy production, nitrogen balance, and the biosynthesis of nucleotides and other amino acids. During viral infections, glutamine metabolism is frequently upregulated to support increased energy demands and nucleotide synthesis required for viral replication. SARS-CoV-2, influenza virus, and RSV infections have been shown to enhance glutaminolysis, contributing to elevated levels of glutamate and downstream metabolites, which provide metabolic intermediates that support viral protein synthesis [141,142,143]. Additionally, glutamine is crucial in maintaining cellular redox balance by serving as a precursor for glutathione synthesis, a major antioxidant [144]. Therefore, while direct evidence linking glutamine depletion to oxidative stress in respiratory virus infections is limited, it is plausible that such depletion could contribute to increased oxidative stress by impairing glutathione production.

In addition to supporting viral replication, glutamine metabolism influences immune cell function. Activated lymphocytes, macrophages, and dendritic cells rely heavily on glutamine for proliferation and cytokine production [145]. Decreased glutamine availability during infection can impair adaptive immune responses, leading to suboptimal viral clearance. Moreover, metabolic competition between the host and the virus for glutamine may exacerbate disease severity by inducing metabolic stress in host cells.

Arginine plays a pivotal role in NO production, which is essential for immune defense [146]. SARS-CoV-2, influenza virus, and RSV infections lead to decreased arginine availability due to increased activity of arginase enzymes [147,148]. This reduction in arginine levels suppresses NO-mediated antiviral responses and promotes an immunosuppressive environment. Studies on patients with severe COVID-19 have shown that arginine depletion is associated with T-cell dysfunction and impaired antiviral immunity [149]. Similarly, influenza and RSV infections trigger a comparable metabolic shift, exacerbating disease severity through immune evasion [150,151].

Arginine metabolism also influences polyamine synthesis, which plays a role in viral RNA stabilization and replication [152]. Some viruses, including SARS-CoV-2, exploit polyamine metabolism to enhance their replication efficiency [153]. Depletion of arginine may, therefore, serve a dual purpose: impairing host immune responses while simultaneously facilitating viral proliferation [154]. Understanding the regulation of arginine metabolism during infection could provide insights into host–virus interactions and potential metabolic vulnerabilities.

Tryptophan metabolism is significantly altered during respiratory viral infections, primarily through activation of the kynurenine pathway by IFN-stimulated enzymes such as indoleamine 2,3-dioxygenase. Elevated serum kynurenine levels have been reported in SARS-CoV-2 infections, correlating with immune suppression and increased inflammatory markers [155,156,157]. Similar findings have been observed in influenza and RSV infections, where tryptophan catabolism reduces serotonin synthesis, impacting mood and immune function. The modulation of this pathway may contribute to the prolonged symptoms observed in post-viral syndromes, including psychological disturbances [158].

Tryptophan metabolism also plays a key role in regulatory T-cell function and immune tolerance. Kynurenine and its downstream metabolites can suppress effector T-cell responses while promoting an anti-inflammatory environment. While beneficial in preventing excessive immune activation, viruses may exploit this mechanism to evade immune detection [159]. Therefore, the balance between tryptophan catabolism and immune activation is critical to disease outcomes in respiratory viral infections.

Cysteine availability is crucial for synthesizing glutathione (GSH), a major antioxidant that protects cells from oxidative stress. GSH synthesis is limited by the availability of cysteine, which serves as a rate-limiting substrate in this process [160]. Respiratory viral infections often lead to GSH depletion, exacerbating oxidative damage and inflammation. For instance, studies have shown that SARS-CoV-2 infection downregulates the nuclear factor erythroid 2-related factor 2 (NRF2) levels and NRF2-dependent gene expression in human airway epithelial cells and in the lungs of mice, leading to reduced antioxidant responses [161]. Similarly, increased ROS production can activate the nuclear factor κB pathway during influenza infections, leading to lung damage. RSV infections have also been associated with oxidative stress and ROS-mediated cellular events [162,163]. Thus, the depletion of cysteine and glutathione plays a critical role in the pathophysiology of these infections. Moreover, GSH influences immune signaling and cytokine production beyond its role in mitigating oxidative stress. Specifically, GSH regulates the activity of transcription factors modulating inflammatory responses [164]. A decline in GSH levels can lead to exaggerated cytokine release, contributing to the hyperinflammatory state observed in severe COVID-19 and other viral pneumonias.

In summary, maintaining adequate cysteine levels is essential for GSH synthesis, which in turn plays a pivotal role in protecting against oxidative stress, regulating inflammation, and ensuring proper immune function during respiratory viral infections.

### 4.2. Nucleotide Metabolism

Respiratory viruses induce significant alterations in the host’s nucleotide metabolism to ensure sufficient nucleotide availability for viral replication and transcription. They upregulate purine and pyrimidine biosynthesis pathways, facilitating rapid viral genome replication. The increased nucleotide demand imposes metabolic stress on host cells, often leading to nucleotide depletion and cellular dysfunction. In response, host cells enhance nucleotide salvage pathways to compensate. However, the competition between host and viral replication can result in nucleotide shortages, impairing DNA repair mechanisms and immune cell proliferation. This metabolic bottleneck and impaired immune function may contribute to the prolonged recovery observed in severe viral infections [165,166,167,168].

SARS-CoV-2, influenza virus, and RSV exploit pyrimidine metabolism, increasing uridine triphosphate and cytidine triphosphate synthesis to support viral genome replication while depleting precursors essential for the host’s adaptive immunity. This metabolic competition can impair T-cell proliferation and antibody production [143,166,169,170]. Similarly, purine metabolism is disrupted, affecting energy balance and immune signaling. ATP and guanosine triphosphate are energy carriers, while purine metabolites such as adenosine modulate immune responses. Altered adenosine metabolism in COVID-19 has been associated with excessive inflammation, while influenza and RSV dysregulate purine pathways, contributing to cytokine storms and prolonged inflammation [171,172]. By modulating de novo biosynthesis and salvage pathways, these viruses create nucleotide imbalances that compromise cellular repair and immune responses, ultimately exacerbating disease severity [173].

Table 3 summarizes the main characteristics of how SARS-CoV-2, influenza virus, and RSV alter amino acid and nucleotide metabolism, contributing to viral replication, immune modulation, and systemic inflammation.

## 5. Oxidative Stress and the Inflammatory Reaction

### 5.1. Intracellular Factors Linking Oxidative Stress and Inflammation

Respiratory viruses trigger increased ROS production through mitochondrial dysfunction, activation of NADPH oxidase (NOX), and inhibition of endogenous antioxidant systems [174,175,176]. Excessive ROS levels produce lipid peroxidation, protein oxidation, and DNA damage, leading to cellular dysfunction. ROS also activate nuclear factor kappa B (NF-κB) and activator protein-1 [177,178], promoting the transcription of pro-inflammatory cytokines, while inflammatory mediators like TNF-α and IL-1β further stimulate ROS production, creating a self-perpetuating cycle of oxidative stress and inflammation [179].

One proposed way virus-induced oxidative stress can enhance the inflammatory response is by activating inflammasomes, which are multiprotein complexes that initiate an immune response [180]. Inflammasomes, such as NLRP3, play a key role in the inflammatory response. ROS activate NLRP3 in macrophages, triggering their assembly and the synthesis of compounds like the chemokine (C-C motif) ligand 2 (CCL2, formerly termed monocyte chemoattractant protein-1, MCP-1), which recruits immune cells to the infection site [181,182]. This chemokine is upregulated after tissue injury and can induce endoplasmic reticulum stress and autophagy and regulate NF-kB expression [183]. 

Pattern recognition receptors are proteins primarily expressed by cells of the innate immune system that detect molecules characteristic of pathogens. They recognize two main classes of molecular patterns: pathogen-associated molecular patterns, which are derived from microbial pathogens, and damage-associated molecular patterns, which originate from host cell components released during cellular damage or death. Recognition of these patterns leads to the activation of NF-κB and the subsequent production of adhesion molecules and chemokines [184,185,186,187]. Additional pathways, such as PI3K, mitogen-activated protein kinase MAPK, and Janus kinase/signal transducers and activators of transcription, further regulate inflammatory responses and cellular stress [188,189].

The unfolded protein response, activated through inositol-requiring enzyme 1, protein kinase R-like endoplasmic reticulum kinase, and activating transcription factor 6, links oxidative stress with endoplasmic reticulum stress and inflammation [190,191,192]. Endoplasmic reticulum stress, driven by CCL2, can modulate inflammation by upregulating C–C chemokine receptor type 2 expression and is linked to cell death and autophagy [193]. Autophagy plays a crucial role in maintaining mitochondrial integrity, and its dysregulation contributes to inflammasome activation and age-related disease susceptibility [194]. Therefore, mitochondrial dysfunction, autophagy impairment, and metabolic alterations are interconnected in the pathology of respiratory viral infections.

### 5.2. Alterations in Endogenous Antioxidant Systems

While inflammation is essential for viral clearance, excessive immune activation can cause severe complications, such as cytokine storms, leading to vascular leakage and multiorgan dysfunction, as seen in severe COVID-19 and influenza cases. Chronic inflammation also depletes antioxidant defenses, increasing cellular vulnerability to oxidative damage.

The organism relies on multiple antioxidant systems to defend against oxidative stress. Respiratory virus infections can profoundly disrupt these protective mechanisms. Among the key components involved in maintaining redox homeostasis and regulating inflammatory responses are GSH, superoxide dismutase (SOD), catalase (CAT), and paraoxonase 1 (PON1). Emerging evidence suggests that although respiratory viruses share common pathogenic pathways, they exert distinct and virus-specific effects on the antioxidant systems they impair.

GSH, the most abundant intracellular non-enzymatic antioxidant, protects cells from oxidative damage by directly scavenging ROS and as a cofactor for glutathione peroxidase. In COVID-19, a marked depletion of GSH has been observed in patients with moderate to severe disease [195,196]. This depletion correlates with elevated oxidative stress markers and pro-inflammatory cytokines, suggesting that inadequate GSH levels may contribute to cytokine storm and tissue injury. Similarly, in influenza virus infection, GSH levels drop significantly in the respiratory tract epithelium [197]. However, in contrast to COVID-19, the depletion appears transient and less systemically profound. In infants, RSV infection also leads to GSH oxidation and disruption of the GSH/GSSG balance [198], exacerbating inflammation and mucus hypersecretion.

SOD, which catalyzes the dismutation of superoxide radicals into hydrogen peroxide and oxygen, is another critical enzymatic defense. There are three isoforms of SOD: cytosolic (SOD1), mitochondrial (SOD2), and extracellular (SOD3) [199]. In SARS-CoV-2 infection, decreased activity of SOD, especially SOD2, has been reported in severe cases [200,201], likely due to mitochondrial dysfunction due to persistent ROS production. In influenza, initial upregulation of SOD occurs as a compensatory response, but this is not sustained in advanced stages of infection [202,203]. RSV also induces oxidative stress with high superoxide levels, and although SOD expression increases early in infection, this upregulation is often insufficient to neutralize the oxidative burden [198].

CAT, responsible for converting hydrogen peroxide into water and oxygen, prevents the accumulation of this toxic intermediate and the subsequent formation of highly reactive hydroxyl radicals. Reduced CAT activity has been reported in SARS-CoV-2, influenza virus, and RSV infections, contributing to redox imbalance and alveolar damage [198,202,204]. 

**Figure 6:**
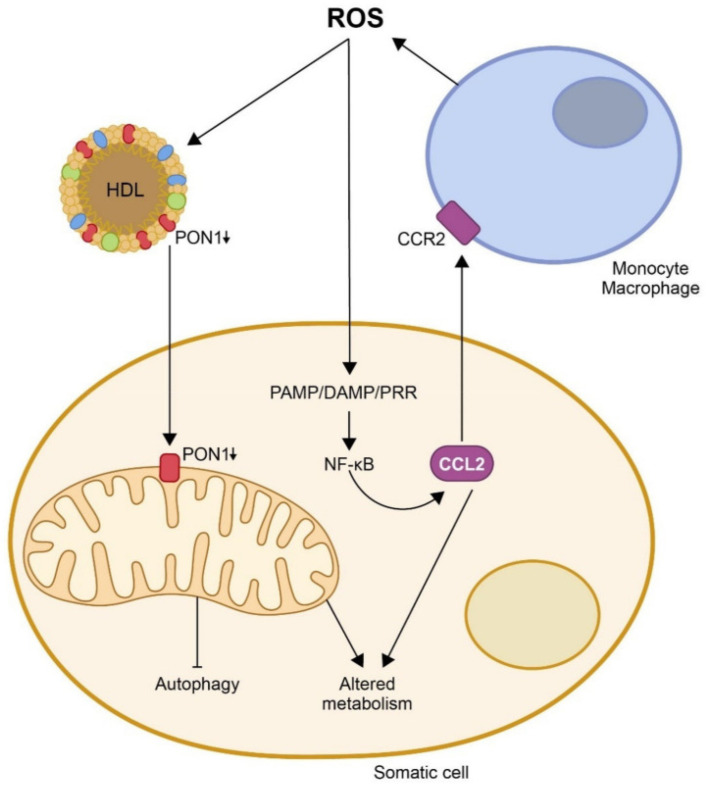
Oxidation, inflammation, and disturbances in energy metabolism are closely related. To date, the evidence reported suggests that excessive production of reactive oxygen species (ROS) would inhibit paraoxonase-1 (PON1) activity in high-density lipoprotein (HDL) particles and in the mitochondrial membranes of somatic cells. At the same time, it would stimulate the synthesis of chemokine (C-C motif) ligand 2 (CCL2) through several pathways, notably that of pathogen-associated molecular patterns/damage-associated molecular patterns/pattern-recognition receptors (PAMP/DAMP/PRR). The decrease in PON1 activity and the increase in CCl2 would cause alterations in mitochondrial metabolism and an inhibition of autophagy. At the same time, CCL2 would interact with its receptor (CRR2) and present on monocytes, promoting their migration to sites of injury, their differentiation to macrophages, and their synthesis of new ROS, producing a vicious circle that would trigger and aggravate the disease. Reproduced from [77]. Copyright by the authors under a CC BY 4.0 license.

PON1, an esterase associated with HDL, plays a multifaceted antioxidant and anti-inflammatory role. It degrades oxidized lipids, modulates macrophage activation, and helps preserve endothelial function (Figure 6) [76,77,78]. Serum PON1 activity is markedly decreased in COVID-19, especially in patients with comorbidities such as diabetes or cardiovascular disease [205,206,207], and the measurement of the serum levels of this enzyme has been proposed as a diagnostic marker [206]. For influenza virus and RSV, the available literature is limited. However, given their known ability to increase oxidative stress and alter HDL structure and composition [75], it is plausible that these infections also lead to changes in PON1 activity similar to those observed in COVID-19, albeit possibly to a lesser extent. 

In comparison, and considering the overall evidence on respiratory viral infections and antioxidant systems, SARS-CoV-2 infection appears to induce a more systemic and persistent oxidative imbalance, likely driven by prolonged viral shedding, endothelial involvement, and dysregulated immune responses. The impact on GSH and PON1 is particularly pronounced, suggesting that these components may be crucial in modulating the severity of COVID-19. Influenza infections, while capable of triggering oxidative stress, often provoke a more localized and transient antioxidant response, with early compensatory increases that may protect against severe tissue damage in immunocompetent hosts. RSV, predominantly affecting infants and older people, leads to intense localized oxidative stress in the lower airways. Depleting GSH and suppressing CAT activity are especially relevant to its pathogenesis. In conclusion, respiratory viruses such as SARS-CoV-2, influenza, and RSV disrupt the host’s antioxidant defense systems through distinct but overlapping mechanisms. The depletion of GSH, reduced activity of SOD and CAT, and suppression of PON1 are key features that contribute to disease severity and tissue damage.

Table 4 summarizes the main changes in endogenous antioxidant systems in infections caused by SARS-CoV-2, influenza virus, and RSV.

## 6. Liquid–Liquid Phase Separation (LLPS) as a Mechanism of Viral Modulation and Metabolic Reprogramming

While we have delineated the key metabolic pathways disrupted by respiratory viral infections, the mechanisms through which viruses orchestrate such broad cellular reprogramming remain incompletely understood. Recent studies have highlighted LLPS as a critical organizing principle in the spatial and temporal regulation of cellular processes. In the context of viral infections, LLPS may facilitate the compartmentalization of viral and host factors, enabling efficient hijacking of metabolic machinery and modulation of host responses. The following section examines the emerging role of LLPS in respiratory virus pathogenesis and metabolic reprogramming, offering a mechanistic framework that may partially unify the diverse observations described above.

LLPS is a biophysical process by which proteins and nucleic acids dynamically condense into membraneless organelles, also known as biomolecular condensates, enabling the compartmentalization of biochemical reactions within the crowded cellular environment [208,209]. These dynamic structures include stress granules, processing bodies, nucleoli, and replication factories. LLPS has emerged as a relevant mechanism in cellular regulation, particularly under stress or infection conditions. Importantly, viruses such as SARS-CoV-2, influenza virus, and RSV have evolved strategies to both utilize and disrupt LLPS to optimize their replication and modulate host responses, with direct and indirect consequences for host cell metabolism.

### 6.1. LLPS in SARS-CoV-2, Influenza, and RSV Infections

Several studies showed that some SARS-CoV-2 proteins possess an intrinsic propensity to undergo LLPS [210]. The nucleocapsid (N) protein, in particular, forms phase-separated condensates with viral RNA, which are believed to facilitate genome packaging and the formation of replication complexes. The N protein also coalesces with stress granule components and modulates their dynamics, impairing host antiviral responses and altering RNA metabolism [211,212,213]. Moreover, the nonstructural protein NSP5, along with other replication-associated proteins, may be recruited to LLPS-driven viral replication compartments, which help shield viral components from immune detection and coordinate essential enzymatic processes [211,214]. The sequestration of host factors within viral condensates, or the disruption of normal condensate formation (e.g., inhibition of stress granules), can interfere with cellular mRNA translation, redox balance, and the distribution of metabolic enzymes. Indirect evidence suggests that SARS-CoV-2 may interfere with glycolysis, at least in part, by displacing glycolytic enzymes such as glyceraldehyde 3-phosphate dehydrogenase (GADPH) and pyruvate kinase M2 (PKM2) from cytoplasmic condensates. In uninfected cells, several glycolytic enzymes, including PKM2 and phosphofructokinase, are known to localize within LLPS compartments, where their spatial organization contributes to metabolic regulation [215]. During SARS-CoV-2 infection, PKM2 expression is preferentially maintained in its dimeric, catalytically less active form, which can impair pyruvate production and divert glycolytic intermediates toward biosynthetic pathways. Furthermore, the viral nucleocapsid (N) protein interacts with host RNA-binding proteins such as YBX1, a key regulator of mRNA stabilization and condensate formation, leading to decreased PKM transcript levels [216]. Together, these findings support the notion that SARS-CoV-2 may exploit LLPS mechanisms to disrupt glycolytic flux and promote metabolic reprogramming in infected cells. In parallel, the suppression of stress granules and other LLPS-mediated stress responses may contribute to prolonged activation of glycolysis and lipid biosynthesis, which are key features of metabolic reprogramming during infection.

The influenza A virus also exploits LLPS to enhance replication efficiency. The viral nucleoprotein and polymerase components form LLPS-like replication compartments in the nucleus, sometimes referred to as “viral inclusions” [217]. These compartments concentrate viral RNA and replication factors, creating microenvironments favorable for genome replication and mRNA synthesis. Additionally, influenza A virus proteins can interact with cellular phase-separated bodies such as stress granules and nuclear speckles, disrupting their function. For example, the NS1 protein of influenza A virus has been shown to antagonize stress granule formation and modulate host mRNA export and translation [218]. Influenza-induced alterations of LLPS structures may perturb nucleocytoplasmic transport and protein quality control pathways, both of which are tightly linked to cellular energy expenditure and metabolic stress [219]. Viral inhibition of stress granules and interference with host LLPS-dependent quality control may promote a shift in metabolic priorities toward biosynthetic processes required for virion production, similar to what is observed in cancer cell metabolism.

In contrast, RSV has been reported to rely on cytoplasmic LLPS mechanisms. RSV nucleoprotein (N) and phosphoprotein (P) drive the formation of cytoplasmic inclusion bodies that serve as viral replication centers [220]. These cytoplasmic inclusion bodies exhibit many hallmarks of LLPS-derived condensates, including dynamic fusion, sensitivity to 1,6-hexanediol, and enrichment of proteins and RNA. Furthermore, they have been shown to sequester innate immune signaling molecules such as the melanoma differentiation-associated protein 5 and MAVS, subverting antiviral responses [221].

The formation of viral inclusion bodies may influence cellular metabolism by creating physical barriers to the diffusion of signaling and metabolic proteins, or by altering the localization of enzymes involved in key pathways such as the tricarboxylic acid cycle, lipid metabolism, or nucleotide biosynthesis. Infected cells may also undergo compensatory reprogramming of mitochondrial function or redox balance in response to viral condensates disrupting normal cellular organization.

### 6.2. LLPS as a Nexus of Viral Control and Metabolic Perturbation

Viral interference with LLPS may have several metabolic consequences. Enhanced glycolysis and suppressed OXPHOS may result from the mislocalization, conformational change, or inhibition of key metabolic enzymes such as PKM2 and GAPDH, which are known to participate in condensates that regulate glycolytic flux [222,223,224,225,226]. In addition, viruses suppress stress granule formation, a process highly dependent on LLPS, to favor translation of viral mRNAs and disrupt host protein synthesis, leading to altered amino acid and nucleotide metabolism [227]. Antioxidant responses are also impaired during infection, partly due to the sequestration or altered phase behavior of redox-sensitive enzymes or NRF2 regulators, which themselves can undergo LLPS [228,229]. Moreover, LLPS-related modulation of mTORC1 signaling and endoplasmic reticulum condensates has been implicated in enhanced lipid biosynthesis during viral infection, particularly in the context of SARS-CoV-2 and influenza, where increased lipid availability supports membrane-bound viral replication complexes [230].

Beyond promoting viral replication, the dysregulation of LLPS has been linked to pathological consequences, including endothelial dysfunction, hyperinflammation, and metabolic syndrome-like manifestations observed in severe cases of COVID-19 and influenza [231,232,233]. These effects underscore the importance of condensate dynamics as a critical interface between viral strategies and host metabolic regulation. Targeting LLPS and the molecular interactions that govern condensate formation may therefore represent a novel therapeutic approach to disrupt viral replication and mitigate metabolic dysregulation during infection.

## 7. Analysis of Peripheral Circulating Metabolites and Search for Metabolic Biomarkers

### 7.1. Omics Disciplines and the Rationale for Metabolic Biomarkers in Viral Infections

In the previous sections, we have reviewed how respiratory viruses induce profound alterations in host cell metabolism, reflecting both viral replication strategies and the host immune response. These metabolic disruptions converge on key pathways, including glycolysis, lipid metabolism, mitochondrial function, amino acid turnover, and redox homeostasis. As a result, metabolites and enzymes involved in these pathways represent promising candidates for biomarkers of disease severity, prognosis, and therapeutic response [96,234]. Early stratification of patients based on metabolic profiles could inform treatment decisions, identify individuals requiring hospitalization, and anticipate complications such as acute respiratory distress or post-viral syndromes [235].

Modern science provides powerful analytical tools for exploring the potential of metabolic parameters as disease biomarkers. In addition to traditional techniques such as spectrophotometry and immunoassays, recent decades have witnessed the emergence of advanced approaches, notably multi-omics. Multi-omics refers to the integrative analysis of multiple layers of biological data to comprehensively understand biological systems. This approach reveals complex interactions among genes, proteins, metabolites, and other biomolecules, offering more profound insights into the mechanisms of health, disease progression, and treatment response [236].

Omics disciplines can be broadly classified into molecular and phenotypic/clinical categories. Molecular omics encompasses genomics, transcriptomics, proteomics, metabolomics, and epigenomics, each focusing on specific molecular components—DNA, RNA, proteins, metabolites, and epigenetic modifications. Phenotypic or clinical omics includes data derived from clinical observations and diagnostic technologies, such as radiomics, pathomics, and hematological omics. Radiomics extracts quantitative features from medical imaging; pathomics integrates molecular profiles with digital pathology and histological data; and hematological omics involves the detailed molecular and cellular analysis of blood to elucidate the complex biology of hematologic conditions [237].

Among these, metabolomics plays a particularly important role [238]. One of its main advantages in clinical research is the relative ease and minimal invasiveness of sample collection, often requiring only a simple blood draw. This practicality facilitates large-scale studies and longitudinal monitoring, making metabolomics a powerful tool in the search for clinically relevant biomarkers in viral infections.

Metabolomics relies on various analytical platforms, each with distinct advantages in terms of sensitivity, specificity, and metabolome coverage. The two most widely employed approaches are mass spectrometry (MS), typically coupled with chromatographic separation techniques such as gas chromatography (GC) or liquid chromatography (LC), and proton nuclear magnetic resonance (^1^H-NMR) spectroscopy [79,239,240,241]. ^1^H-NMR-based metabolomics offers high reproducibility, non-destructive analysis, and minimal sample preparation, making it particularly well-suited for longitudinal or large-scale comparative studies. However, its relatively low sensitivity can limit the detection of low-abundance metabolites. In contrast, MS-based methods—especially when combined with chromatographic separation—enable high sensitivity and broad metabolite coverage, allowing for the detection of compounds at very low concentrations [242,243]. Targeted metabolomics focuses on predefined sets of metabolites, often associated with specific pathways or conditions, offering high accuracy and quantitative precision. Untargeted metabolomics, on the other hand, aims to profile as many metabolites as possible without prior bias, facilitating the discovery of novel biomarkers or previously unrecognized metabolic alterations [244,245,246]. Together, these complementary approaches provide a robust framework for elucidating metabolic signatures associated with viral infections and identifying potential diagnostic or prognostic biomarkers.

The term biomarker is frequently and, at times, indiscriminately used in the biomedical literature. However, the mere presence of a statistically significant difference in the levels of a given parameter between two clinical conditions is not sufficient to classify that parameter as a biomarker. An accurate biomarker is a measurable indicator that distinguishes between two well-defined biological states, such as health versus disease, favorable versus poor prognosis, or responsiveness versus resistance to a specific treatment [247,248]. For a parameter to fulfill this role, it must enable unambiguous discrimination between these conditions, ideally with minimal or no overlap in their respective distributions. This characteristic implies exceptionally high diagnostic performance, typically characterized by sensitivity and specificity values approaching 100% and an area under the receiver operating characteristic (ROC) curve (AUC) nearing 1.0 [249]. These stringent criteria are rarely met, particularly in the context of metabolic biomarkers.

This section reviews the most relevant studies identifying candidate metabolites as biomarkers of respiratory viral infections. We also critically evaluate their diagnostic accuracy and practical limitations.

### 7.2. Lipid Signatures and the Lipidome

A comparative lipidomic analysis revealed significant alterations in serum lipid mediators between COVID-19-positive patients and healthy individuals, with elevated levels of O-octanoyl-L-carnitine (CAR 8:0) and lysophosphatidylethanolamine (LPE), and decreased levels of arachidonic acid and oxylipins like 9/13-HODE and 15-HETE [250]. Elevated CAR 8:0 suggests mitochondrial dysfunction, a known feature of viral infections, including COVID-19, reflecting impaired fatty acid β-oxidation and potential lung injury through surfactant inhibition [251,252,253]. However, similar lipid disturbances were observed in COVID-19-negative patients with bacterial infections, indicating a general inflammatory response rather than a SARS-CoV-2 effect. To refine specificity, comparisons with COVID-19-negative patients with bacterial infections highlighted an increased phosphatidylcholine/lysophosphatidylcholine (PC/LPC) ratio in COVID-19 patients, showing strong diagnostic potential (AUC = 0.950) [250]. Nonetheless, the generalizability of this marker is limited by inconsistent findings across studies, likely due to differences in disease severity, patient comorbidities, and the choice of control groups. Indeed, prior studies have reported varying patterns, including decreased PC with increased LPC, the reverse, or simultaneous reductions in both lipid classes [254,255,256,257,258,259,260].

Lipidomic profiles of lung cells further underscore disease-specific patterns. COVID-19-positive patients exhibited elevated long-chain triglycerides, particularly in immune cells, which could modulate inflammatory signaling. Similar serum triglycerides, VLDL, and polyunsaturated fatty acid elevations have been noted in Ebola and COVID-19 infections [261,262,263,264]. Importantly, some lipid species—like long-chain triglycerides, PC 36:5, LPC 22:6-sn2, PC 36:1, and secondary bile acids—were distinctly altered between COVID-19-positive and -negative groups. These may contribute to long-term cardiovascular risk and point to potential post-discharge interventions, such as dietary or lipid-lowering strategies [250].

One of the most promising findings was the significant reduction in arachidonic acid in COVID-19 patients, a pattern also observed in cases of influenza A and RSV infections [265]. This result holds practical relevance, as arachidonic acid benefits from the availability of specific calibrators and antibodies, enabling the development of simple, cost-effective assays suitable for routine use in general clinical laboratories—unlike more complex lipids such as LPC or PC. Arachidonic acid plays a central role in both the synthesis of inflammatory mediators and the formation of viral membranes. Notably, in vitro studies have shown that its supplementation can inhibit viral replication [266]. Its depletion in patients likely reflects increased utilization for host immune responses or viral replication requirements and has been associated with disease severity [250,267].

Recently, a novel biosensing platform has been developed using a competitive immunoassay integrated with magnetic microbeads and screen-printed carbon electrodes to quantify arachidonic acid in serum. This system demonstrated high sensitivity, reproducibility, and practicality for clinical settings, with strong concordance with conventional assays [268]. Although still at the prototype stage and not commercially available, the platform holds significant promise for point-of-care diagnostics and the personalized assessment of vaccine responses.

Lipoprotein alterations detected by ^1^H-NMR spectroscopy have provided valuable insights into host metabolic responses to respiratory viral infections. In COVID-19, several studies have shown a marked reduction in HDL particle numbers—especially small HDL—together with increased levels of triglyceride-rich lipoproteins. These changes correlate with systemic inflammation, elevated branched-chain amino acids, and β-hydroxybutyrate, indicating altered energy and hepatic metabolism [80,84]. Inflammatory cytokines, such as IL-6 and IFN-γ, show positive correlations with LDL subfractions and inverse associations with HDL, confirming a link between immune and lipid responses [81].

Recent work by the International COVID-19 Research Network—a global collaboration focused on metabolomics—has contributed significantly to our understanding of how COVID-19 affects human metabolism. Utilizing the B.I. platform, a standardized and automated ^1^H-NMR-based system specifically tailored for biofluid analysis, this consortium has shown that SARS-CoV-2 infection induces consistent and marked shifts in the metabolic profiles of patients. These alterations include notable changes in concentrations of various metabolites (e.g., mannose, pyruvate, and 3-hydroxybutyrate) as well as in the composition of specific lipoprotein subfractions. Importantly, many of these metabolic signatures appear to correlate with disease severity [269]. In parallel, another widely adopted approach developed by Nightingale Health has used pre-pandemic serum samples to identify metabolic traits linked to vulnerability to severe COVID-19. This research revealed that lower levels of cholesterol, omega-3 and omega-6 fatty acids, and albumin, alongside elevated glycoprotein A, may serve as predictive markers of disease progression [270]. 

In a multicenter Spanish cohort, ^1^H-NMR-based machine learning models using lipoprotein profiles accurately predicted COVID-19 severity [271]. Persistent abnormalities in HDL and triglyceride-rich lipoproteins have also been reported months after recovery, suggesting a potential role in the development of long-term complications [264,272,273].

Although less studied, influenza and RSV infections also induce lipoprotein alterations. Influenza A has been associated with reduced HDL and elevated triglycerides, and lower HDL levels predict worse outcomes [86]. In RSV, a lipidomic study of infants hospitalized for bronchiolitis revealed specific lipid changes—e.g., in phosphatidylcholine and dihydroceramides—associated with severity [274].

These findings highlight the relevance of lipoprotein profiling in respiratory infections. However, pre-analytical variables, such as sample inactivation, may introduce artifacts [275], and distinguishing infection-specific signatures from general inflammatory responses remains a challenge [84]. Moreover, comorbidities and treatment effects must be considered when interpreting metabolic data [276,277,278].

### 7.3. Altered Carbohydrate Flux, Energy Imbalance, and Perturbations in Amino Acid and Nucleotide Metabolism

Comparative studies analyzing the serum metabolomic profiles of healthy individuals, COVID-19-positive patients, and COVID-19-negative patients with infections have revealed pronounced differences in pentose glucuronate interconversion, ascorbate and fructose metabolism, the nucleotide sugar biosynthetic route, as well as nucleotide and amino acid metabolic processes, all of which are related to carbohydrate and energy metabolism [279]. Among these routes, pentose and glucuronate interconversion emerged as the most characteristic alteration observed in COVID-19-positive patients, with notably elevated activity compared to COVID-19-negative individuals and healthy controls. This pathway is crucial for detoxification, wherein d-glucuronic acid conjugates with hydroxyl or amino groups of toxic compounds under the catalysis of UDP-glucuronosyltransferase, enhancing their water solubility and facilitating excretion through bile or urine [280]. Although investigations into this metabolic alteration in COVID-19 are still limited, recent reports have associated enhanced pentose and glucuronate interconversion with microbiome disruptions in patients suffering from oral infections [281,282]. Additionally, pharmacological studies have indicated that the modulation of this pathway could be a mechanism through which certain anti-inflammatory drugs exert their effects in both human subjects and animal models [283,284].

During viral transcription, the energy requirements and precursor molecules for building viral structural components are primarily supplied by an upregulation of aerobic glycolysis and activation of the pentose phosphate pathway [285,286]. Enhanced aerobic glycolysis increases the function of hexokinase, the enzyme that controls the pace of glycolytic flux, consequently supporting the activation of the pentose phosphate pathway. Hexokinase initiates this process by phosphorylating glucose to form glucose-6-phosphate, which is then oxidized by glucose-6-phosphate dehydrogenase within the pentose phosphate pathway to produce ribose-5-phosphate. Ribose-5-phosphate is essential for nucleotide biosynthesis, while sugar-phosphate intermediates derived from this pathway are also critical for producing amino acids and NADPH [287,288]. Several viruses, such as the influenza virus, SARS-CoV-2, hepatitis C virus, and HIV-1, have been shown to enhance the activity of the pentose phosphate pathway [289,290,291]. Overall, these findings highlight significant disruptions in pathways linked to energy production, nucleotide synthesis, and amino acid metabolism, which are highly interconnected and exert mutual influences.

Researchers have also explored whether metabolic profiles differed among COVID-19 patients according to comorbidities and disease severity. Particularly notable were the observations related to the severity of illness. Volcano plot analyses revealed that individuals who required intensive care unit (ICU) admission or who succumbed to the disease exhibited elevated serum levels of lauric acid [279]. When ingested from oils, lauric acid is metabolized into laurate-monoglyceride, a compound capable of inactivating enveloped viruses by disrupting the viral membrane and preventing viral entry into host cells [292,293]. Additional studies have described that laurate-monoglyceride can disintegrate viral envelopes, leading to viral inactivation [294]. At first glance, these findings may seem paradoxical, as higher lauric acid levels were associated with worse clinical outcomes. A potential explanation proposed is that free lauric acid may not display the same virucidal activity as its monoglyceride form; thus, elevated levels of free lauric acid might correspond with reduced amounts of the active monoglyceride form. Alternatively, an upsurge in lauric acid might reflect an attempt by the body to boost monoglyceride synthesis in response to viral infection. These hypotheses point to a promising area of future investigation into the relationship between lauric acid dynamics and COVID-19 severity.

Furthermore, lower xylitol concentrations were documented in patients requiring intensive care compared to less severe cases. Xylitol, a metabolite derived from the pentose and glucuronate interconversion pathway, has been recognized for its anti-inflammatory, antiglycemic, antiviral, and antibacterial actions in the context of pulmonary infections [295]. It has been shown that xylitol can reduce salt concentrations in the airway surface liquid of the lungs, thereby enhancing antibody function [296]. In vitro research has further demonstrated that xylitol-treated macrophages show a tenfold reduction in adhesion capacity relative to untreated controls, alongside diminished expression of adhesion molecules, a key process in modulating pulmonary inflammatory responses [297]. Additional preclinical studies have reported that dietary xylitol supplementation reduces viral loads in mice infected with human RSV or influenza A virus [296,298].

Moreover, machine learning-based analyses [279] identified maltose, glyceric acid, mannonic acid, xylitol, and erythronic acid as the most discriminative metabolites for distinguishing COVID-19-positive patients from healthy controls (AUC = 0.98). In contrast, the combination of succinic, phosphoric, and malic acids, hypoxanthine, and S-adenosylhomocysteine most effectively differentiated COVID-19-positive from COVID-19-negative individuals (AUC = 0.85). 

The biological significance of these observations is not straightforward. Since maltose, mannonic acid, and erythronic acid are predominantly plant-derived compounds and are not endogenously synthesized in large amounts by humans, it has been hypothesized that changes in their serum concentrations may be influenced by alterations in gut microbiota secondary to infection. Indeed, the concept of a gut–lung axis has been proposed, suggesting that respiratory infections can impact the gut microbiota and vice versa, ultimately manifesting in altered circulating metabolite profiles [299]. Dysbiosis of the gut microbiome has been implicated in respiratory diseases and infections [300], and antibiotic-induced microbiome disruptions have been shown to influence lung disease outcomes [301]. Moreover, modifications to the lung microbiota are known to affect gut microbial communities reciprocally [302]. Several studies have connected alterations in maltose, mannose, succinate, and erythronic acid serum levels with changes in the gastrointestinal microbiome [303,304,305,306,307]. Recently, a multiomics investigation further detailed complex networks linking gut microbes, metabolites, and cytokines in the context of COVID-19 [308]. Of particular interest, shifts in the gut microbiota have been associated with changes in metabolites belonging to the pentose and glucuronate interconversion pathway [309,310,311].

Although reprogramming of carbohydrate and energy metabolism is a shared feature among respiratory viral infections, the specific signatures of SARS-CoV-2 differ in essential ways and suggest the presence of unique metabolic biomarkers. Influenza virus, for instance, also enhances glycolysis and pentose phosphate pathway (PPP) activity to support viral replication [70,142,312], but does not appear to induce the same degree of alteration in detoxification-related pathways, such as the pentose and glucuronate interconversion route, which is markedly affected in COVID-19. Studies exploring the utility of such detoxification pathway metabolites as biomarkers in influenza are limited or nonexistent.

RSV infection, on the other hand, promotes mitochondrial fragmentation and significantly alters amino acid metabolism, especially glutamine and arginine pathways [313]. While these findings are mechanistically relevant, systematic analyses to identify consistent metabolic biomarkers of RSV infection remain scarce.

### 7.4. Redox Imbalance and the Antioxidant Response

GSH is the most abundant intracellular antioxidant and a critical regulator of redox homeostasis. GSH depletion and a decreased GSH/GSSG ratio have been consistently reported in viral infections. However, the severity and persistence of these changes appear greater in COVID-19 compared to influenza and RSV. A mechanistic hypothesis [195] posits that endogenous GSH deficiency may be a central factor in developing severe COVID-19 manifestations, due to unchecked oxidative stress and a weakened immune response. Clinical studies have since confirmed that patients hospitalized with COVID-19 present significantly lower plasma GSH levels, which correlate with elevated markers of systemic inflammation [314,315]. Although no robust ROC-based metrics have been reported for GSH alone, its measurement contributes valuable prognostic information when incorporated into broader biomarker panels.

SOD and CAT are key enzymatic antioxidants that neutralize superoxide radicals and hydrogen peroxide, respectively. Respiratory virus infections usually elicit a short-lived increase in their activities, followed by suppression or depletion in advanced stages of the disease [316]. This dynamic shift is indicative of sustained oxidative pressure and impaired mitochondrial function. While SOD and CAT have been included in some prognostic models, their individual diagnostic accuracy, measured by sensitivity, specificity, or AUC, remains insufficiently documented.

PON1 activity is reduced during viral infections, but the decrease is especially pronounced in COVID-19 compared to influenza and RSV. Hospitalized COVID-19 patients showed significantly reduced PON1 arylesterase activity [205]. In a model designed to differentiate COVID-19 from other acute respiratory syndromes, PON1 activity demonstrated a sensitivity of 84%, specificity of 79%, and an AUC of 0.87, making it a promising biomarker for differential diagnosis [206].

Although not classical antioxidants, the tryptophan–kynurenine pathway metabolites are modulated by oxidative stress and systemic inflammation. In COVID-19, there is a sustained increase in the kynurenine/tryptophan (Kyn/Trp) ratio, associated with immune activation and disease severity. A metabolomic study reported a high discriminatory performance (AUC = 0.95) for distinguishing COVID-19-positive from COVID-19-negative patients; however, the authors cautioned that these findings should be interpreted carefully due to the limited sample size [156].

Summarizing the information presented in this section, redox imbalance emerges as a shared hallmark of respiratory viral infections, yet accumulating evidence points to distinct antioxidant and metabolic signatures for each virus. In COVID-19, key alterations include a marked reduction in GSH and PON1, a consistently elevated kynurenine-to-tryptophan ratio, sustained SOD downregulation, and increased levels of oxidative lipid byproducts such as 4-hydroxynonenal and malondialdehyde. In contrast, influenza virus infection is generally associated with transient oxidative stress, with moderate GSH depletion, variable upregulation of enzymatic antioxidants such as SOD and CAT, and subsequent normalization of redox markers during convalescence. RSV infection shows a different pattern, featuring early mitochondrial oxidative injury, GSH depletion during the acute phase, and increased lipid peroxidation. However, data on enzymatic antioxidant responses in RSV remain limited.

These findings suggest that the combined presence of low GSH and PON1 activity, along with elevated kynurenine levels and oxidative lipid metabolites, may constitute a distinctive redox-based biosignature for COVID-19. Importantly, this biosignature not only differentiates COVID-19 from healthy controls, but the results indicate that it may also help to distinguish it from other respiratory viral infections.

## 8. Identification of Potential Therapeutic Targets

The metabolic reprogramming induced by respiratory viral infections creates vulnerabilities that can be exploited for therapeutic purposes. Beyond the canonical antiviral approaches, targeting host metabolic pathways altered during infection provides an opportunity to modulate viral replication, control inflammation, and mitigate long-term sequelae. This section identifies promising therapeutic targets emerging from preclinical and clinical studies on SARS-CoV-2, influenza, and RSV, focusing on host metabolic and redox pathways.

### 8.1. Inhibiting Viral Entry

Several host factors crucial for viral entry are modulated by cellular metabolic pathways, offering indirect avenues for antiviral intervention. For instance, the expression of the transmembrane protease, serine 2 (TMPRSS2) and ACE2, key entry factors for SARS-CoV-2, can be influenced by metabolic status and hormonal regulation linked to insulin signaling and energy-sensing pathways such as AMPK. Similarly, the fusion machinery of RSV depends on host membrane lipid composition and cholesterol content, both shaped by metabolic activity. 

These insights have prompted the exploration of drugs that modulate host metabolism to interfere with viral entry. Camostat mesylate, a TMPRSS2 inhibitor, has shown promise in preclinical models by blocking SARS-CoV-2 spike protein priming, thus preventing cell entry [19]. By altering cholesterol synthesis and lipid raft composition, statins may reduce viral attachment and assembly, with some observational studies reporting improved outcomes in viral pneumonia [89]. Chloroquine and hydroxychloroquine, despite their controversial clinical use, were found to interfere with endosomal pH and glycosylation of ACE2, thereby affecting SARS-CoV-2 entry in vitro [317].

Amantadine and rimantadine act on the M2 ion channel protein of influenza A virus, inhibiting viral entry [318,319]. Unfortunately, the high mutation rate of the M2 protein has led to widespread resistance among influenza A virus strains, so these compounds are no longer recommended [320]. More promising results have been obtained with DAS-181, a drug that cleaves sialic acid receptors on respiratory epithelial cells. Preliminary clinical trials have indicated that it may effectively block infection by the influenza virus [321].

Palivizumab is a monoclonal antibody that specifically targets the protein inducing fusion of RSV to the host epithelial cell membranes (protein F). This compound is used prophylactically to prevent serious lower respiratory tract infections in high-risk children [322]. Some other compounds targeting the F protein have been identified in recent studies, and several of these inhibitors, including rilematovir (JNJ-53718678), ziresovir (AK-0529), presatovir (GS-5806), and sisunatovir (RV-521), have shown therapeutic efficacy in individuals challenged with RSV [323].

### 8.2. Targeting Lipid Metabolism

Various studies have highlighted the reliance of respiratory viruses on host lipid metabolism, including de novo lipogenesis and cholesterol trafficking. FASN inhibitors, such as orlistat, disrupt viral envelope formation and replication in enveloped viruses by interfering with host lipid biosynthesis, and have shown antiviral activity in vitro against SARS-CoV-2, influenza, and RSV [52,324].

Statins, which inhibit HMG-CoA reductase and reduce cholesterol biosynthesis, have demonstrated anti-inflammatory and potential antiviral effects in observational studies of COVID-19, with meta-analyses suggesting a protective effect on mortality [325,326,327,328]. 

Omega-3 fatty acids and their specialized pro-resolving mediators, including resolvins and protectins, have also emerged as modulators of inflammation in viral infections and may counteract the persistent inflammatory response observed in long COVID-19 [329].

Data on the effect of statins and omega-3 fatty acids on influenza and RSV infections are limited and inconclusive. While some studies suggest statins may reduce influenza prevalence and mortality, others indicate they might increase the risk of common infections. Similarly, omega-3 fatty acids show potential benefits in some cardiovascular studies, but their impact on viral infections is not well-established. Further research is needed to clarify the role of these treatments in preventing or treating viral respiratory illnesses [330,331,332].

### 8.3. Inhibiting Glycolysis

The enhanced glycolytic flux activation observed in infected cells reflects a shift toward anabolism. Inhibiting key glycolytic enzymes such as hexokinase 2 or 6-phosphofructo-2-kinase/fructose-2,6-biphosphatase 3 (PFKFB3) can reduce viral replication and inflammation. For instance, 2-deoxy-D-glucose has shown efficacy in reducing SARS-CoV-2 replication in vitro and in clinical trials in India, where it shortened oxygen dependency and hospital stay in moderately ill patients [333,334,335,336]. Similarly, inhibition of PFKFB3 has shown potential in reducing viral replication and inflammatory responses in preclinical models of influenza. Recent studies, such as those using the selective inhibitor KAN0438757 [337], demonstrated significant suppression of H1N1 influenza virus replication and cytokine production in human epithelial cells [338]. In addition, although the compound 3-(3-pyridinyl)-1-(4-pyridinyl)-2-propen-1-one (3PO) has also been reported to attenuate influenza-related inflammation, its specificity as a PFKFB3 inhibitor is debated, suggesting that its observed effects may involve off-target mechanisms [339,340,341]. Therefore, while targeting PFKFB3 is a promising therapeutic strategy, further studies using more selective inhibitors are needed to validate its efficacy and mechanism of action.

### 8.4. Modulating Mitochondrial Dynamics and Bioenergetics

One promising therapeutic avenue is the modulation of mitochondrial dynamics, especially the inhibition of excessive mitochondrial fission, which is associated with inflammation and cell death. Mitochondrial division inhibitor 1 (Mdivi-1), a selective inhibitor of Drp1, prevents mitochondrial fragmentation by blocking Drp1-mediated fission. Studies in experimental animals showed that Mdivi-1 reduces viral-induced mitochondrial fragmentation, restores mitochondrial membrane potential, and attenuates inflammatory cytokine release [342,343].

Moreover, mitochondrial protectants such as melatonin, a potent antioxidant and regulator of mitochondrial homeostasis, have demonstrated anti-inflammatory and antiviral properties in COVID-19 models. Melatonin reduces oxidative stress, supports mitochondrial biogenesis via PGC-1α activation, and modulates immune responses [344]. Coenzyme Q10, another mitochondrial antioxidant, may similarly improve mitochondrial respiration and reduce oxidative injury [345,346], although clinical data in viral infections remain limited.

Other pharmacological candidates targeting mitochondrial metabolism include metformin, which supports mitochondrial function via AMPK activation and reduces pro-inflammatory signaling in viral pneumonia [347,348], and Szeto–Schiller (SS) peptides, such as SS-31, which selectively target the inner mitochondrial membrane, reduce mitochondrial ROS, and improve mitochondrial efficiency in models of oxidative stress [349]. Despite their promise in models of ischemia, neurodegeneration, and metabolic dysfunction [350,351,352,353], no published evidence supports the use of SS peptides in viral infections. Nevertheless, their mechanisms of action align well with the known mitochondrial disturbances induced by respiratory viruses, making them candidates for future investigation.

Preserving mitochondrial function and preventing mitochondrial fragmentation represent rational strategies to limit inflammation, promote cellular survival, and improve outcomes in respiratory viral infections. Drugs such as Mdivi-1, melatonin, and SS-31 illustrate the potential of this approach, which warrants further investigation in clinical settings. Agents enhancing OXPHOS, such as dichloroacetate, may also counteract the Warburg-like phenotype in infected cells and restore energy balance [354]. Moreover, supplementation with mitochondrial cofactors like coenzyme Q10 and nicotinamide riboside has been proposed to alleviate COVID-19-related mitochondrial dysfunction [355,356].

Experimental evidence from COVID-19, influenza, and RSV models supports the broader applicability of this therapeutic approach. In influenza-infected cells and mice, mitochondrial fission is induced via Drp1 activation, contributing to inflammation and lung injury [357]. RSV also disrupts mitochondrial morphology and function via similar mechanisms, with Drp1 inhibition restoring mitochondrial integrity and reducing cytokine release [358]. Moreover, melatonin and metformin have shown protective effects in experimental models of respiratory virus infections by reducing oxidative stress and preserving mitochondrial function, confirming mitochondrial targeting as a shared antiviral strategy [359,360].

### 8.5. Antioxidant System Modulation

N-acetylcysteine (NAC), a well-known precursor of cysteine and GSH, has been widely studied among antioxidant therapies. In patients with COVID-19, NAC administration has been associated with improved oxygenation, reduction in inflammatory markers, and lower risk of mechanical ventilation in observational studies [361,362]. Studies in cultured A549 lung cancer cells showed that NAC inhibited oxidant-sensitive pathways, including NF-κB and mitogen-activated protein kinase p38. Moreover, it reduced viral replication and production of pro-inflammatory molecules [363], and prophylactic benefits by NAC were observed in elderly individuals during influenza seasons [364].

Melatonin, an endogenous molecule with antioxidant and anti-inflammatory properties, upregulates enzymatic defenses such as SOD and CAT, and downregulates NF-κB-mediated cytokine production. In preclinical models of viral infections, including RSV and influenza, melatonin treatment has reduced pulmonary inflammation and oxidative stress [365,366,367]. Recent preclinical and clinical studies are evaluating its efficacy as an adjuvant therapy in COVID-19 [344,368,369,370].

Tempol, a membrane-permeable nitroxide that mimics SOD activity, has shown efficacy in preclinical models by neutralizing superoxide radicals, reducing lipid peroxidation, and attenuating inflammation [371]. Tempol treatment significantly reduced viral replication and lung pathology in Syrian hamsters infected with SARS-CoV-2. The treated animals showed decreased viral RNA levels in the lungs, reduced infectious virus titers, and less severe histopathological changes compared to control groups [372], though human studies are lacking.

Ebselen, a glutathione peroxidase mimetic, has shown dual action in COVID-19 in an in silico study. This compound possesses direct antiviral activity through the inhibition of the SARS-CoV-2 main protease (Mpro) and has antioxidant effects [373].

Despite the growing interest in these antioxidant strategies, the therapeutic modulation of PON1 in viral infections remains largely unexplored. Although reduced PON1 activity has been consistently reported in COVID-19 and associated with disease severity, no pharmacological interventions have been developed to restore PON1 function. Experimental approaches to increase PON1 activity, such as dietary polyphenols, HDL-targeted therapies, or gene modulation, have been proposed in cardiovascular and metabolic diseases [374,375]. However, no clinical or preclinical studies have evaluated their efficacy in respiratory viral infections. This gap highlights a potential opportunity for future research, particularly given PON1′s role in neutralizing lipid peroxides and modulating inflammatory responses. Whether increasing PON1 activity could mitigate oxidative stress or reduce complications in viral infections such as COVID-19 or influenza remains an open question.

### 8.6. Modulation of the Kynurenine Pathway

The kynurenine pathway is the principal route of tryptophan catabolism, and its activation plays a central role in immune regulation, oxidative stress, and metabolic control during viral infections. One of its key regulatory enzymes, indoleamine 2,3-dioxygenase 1 (IDO1), is upregulated in response to inflammatory cytokines, particularly IFN-γ, leading to increased conversion of tryptophan into kynurenine and other downstream metabolites. In COVID-19, a persistently elevated Kyn/Trp ratio has been consistently associated with disease severity, lymphopenia, and systemic immune dysregulation [156,257]. This metabolic shift serves both antiviral and immunoregulatory purposes. On the one hand, tryptophan depletion can limit viral replication, but on the other hand, kynurenine and its metabolites exert immunosuppressive effects by inducing regulatory T cells (Tregs), inhibiting effector T cell responses, and modulating dendritic cell function [376]. Kynurenine also acts as an endogenous ligand of the aryl hydrocarbon receptor (AhR), a transcription factor that regulates mucosal immunity, barrier function, and inflammatory cytokine production [377]. Chronic activation of AhR by elevated kynurenine levels may contribute to persistent inflammation and immune exhaustion in severe COVID-19 and possibly other viral infections.

Given its role in immune escape and homeostasis, the kynurenine pathway has emerged as a potential therapeutic target, particularly in diseases marked by chronic inflammation and immune dysfunction. Inhibitors of IDO1, such as epacadostat and navoximod, have been tested extensively in oncology for their ability to reverse immune suppression and restore antitumor immunity [378,379,380]. Although these agents have not yet been tested in large trials for viral infections, their mechanism of action may be relevant for conditions such as severe COVID-19, where elevated kynurenine activity correlates with worse outcomes [257].

In addition to IDO1 blockade, targeting downstream components of the pathway may offer therapeutic opportunities. For instance, kynurenic acid and quinolinic acid exert neuroactive and pro-oxidant effects, and the possibility exists that they are implicated in the pathophysiology of viral encephalopathy [381]. Another promising approach is modulating the AhR, which is activated by kynurenine and related metabolites. Selective AhR antagonists or modulators, such as CH223191, have been shown to reduce the traffic-derived particulate matter-induced cytokine release in human macrophages and bronchial epithelial cells [382].

Nutritional modulation of the kynurenine pathway is also under investigation. Niacin (vitamin B3), a downstream product of the pathway, may act as a feedback regulator of tryptophan metabolism and has been proposed as an adjunctive therapy to buffer oxidative stress and inflammation [383]. Furthermore, gut microbiota composition influences tryptophan availability and kynurenine pathway activity, suggesting that probiotic or microbiome-directed interventions might indirectly affect this immunometabolic axis [384].

Despite these promising avenues, no therapeutic interventions targeting the kynurenine axis have yet been approved or validated in acute viral respiratory infections. Given the strong correlation between the kynurenine pathway and disease severity in COVID-19, this biochemical route represents a compelling target for future translational research.

Table 5 provides an overview of the principal drugs investigated, their associated metabolic pathways, and the viral infections in which they have been proposed as therapeutic agents.

### 8.7. Multi-Omics Guided Therapies and Personalized Interventions

Multi-omics approaches provide a comprehensive, systems-level view of disease pathophysiology, enabling the identification of biosignatures that serve as robust diagnostic or prognostic biomarkers and reveal actionable therapeutic targets. Rather than isolated molecular readouts, multi-omics facilitates the construction of dynamic interaction networks that map the cascade of host responses, from gene expression to metabolite accumulation, offering unprecedented precision for therapeutic intervention.

In the context of respiratory viral infections, this holistic approach allows for the stratification of patients based on their individual metabolic and immune profiles. For instance, redox status, lipidomic alterations, and amino acid imbalances can guide the personalized use of antioxidant regimens, lipid-modulating agents, amino acid supplementation, or targeted metabolic inhibitors. Notably, integrating metabolic and immune data can help identify patient subgroups more likely to benefit from specific interventions, minimizing ineffective or potentially harmful treatments [236,385].

One prominent example is the recognition of arginine deficiency in severe COVID-19, derived from metabolomic analyses showing significant depletion of circulating arginine levels. This finding has led to the hypothesis that therapeutic arginine depletion or modulation might influence immune responses and viral replication. Pegylated arginase (PEG-Arg1), which depletes extracellular arginine, has been proposed as a potential therapy to modulate hyperinflammation in COVID-19 [386]. Similarly, glutamine metabolism, which is altered in both influenza and SARS-CoV-2 infections, has been proposed as a therapeutic target, with inhibitors of glutaminolysis showing antiviral effects in preclinical models [387].

Furthermore, machine learning-enhanced metabolomics has demonstrated that combinations of metabolites (e.g., maltose, xylitol, glyceric acid) can differentiate COVID-19 patients from healthy controls and patients without COVID-19 with high accuracy. These panels offer diagnostic utility and may assist in therapeutic stratification, identifying patients with specific metabolic phenotypes that respond better to tailored interventions [279]. 

Another key application of multi-omics is in the monitoring of therapeutic response. Changes in metabolic or lipidomic profile during treatment can serve as dynamic biomarkers, providing real-time feedback on drug efficacy or therapeutic adjustment [250,279]. While integrating multi-omics data holds promise for personalizing pharmacological or nutritional interventions in respiratory viral infections, current clinical trials have not yet adopted this approach. Existing studies on omega-3 supplementation provide valuable insights but do not utilize patient-specific omics data to tailor interventions. Further research is needed to develop and assess personalized nutritional strategies guided by comprehensive omics profiling [388,389].

Ultimately, the multi-omics framework paves the way for a multi-therapeutic approach, combining pharmacological and nutritional strategies adapted to the individual molecular profile. For example, a patient showing GSH depletion, dysregulated kynurenine metabolism, and altered mitochondrial lipid composition could be managed with a tailored regimen combining NAC, AhR modulators, and mitochondrial stabilizers. This personalized, multi-targeted intervention model represents a shift from disease-based to mechanism-based precision medicine, especially relevant for complex viral syndromes like COVID-19 or severe influenza.

However, translating multi-omics findings into clinical practice poses significant challenges, including data standardization, analytical complexity, cost, and interdisciplinary coordination. Nonetheless, ongoing systems biology and computational medicine efforts are progressively overcoming these barriers, bringing personalized multi-therapeutic strategies closer to clinical application [390,391].

## 9. Conclusions and Future Perspectives

Respiratory viral infections such as those caused by SARS-CoV-2, influenza virus, and RSV continue to exert a significant global health burden. Although distinct in structure and host tropism, these viruses share a common feature: the ability to hijack and reprogram host metabolic pathways to facilitate their replication, evade immune responses, and modulate disease progression. This review has detailed how respiratory viruses manipulate lipid metabolism, energy production, amino acid and nucleotide pathways, and oxidative stress responses, often driving the host cell into a pro-viral metabolic state.

A key advance in recent years has been integrating multi-omics technologies, allowing for a more comprehensive understanding of the host response to infection. These tools facilitate the discovery of biosignatures with diagnostic and prognostic value and highlight actionable metabolic targets. In particular, personalized therapeutic approaches, such as antioxidant regimens tailored to redox imbalance, or amino acid and lipid-based interventions guided by metabolomic signatures, have emerged as promising strategies. Furthermore, targeting virus-induced mitochondrial dysfunction, glycolysis, or pathways like kynurenine metabolism holds translational potential.

While these conceptual and technical advances have been largely propelled by the intense global research efforts on COVID-19, there is a growing recognition that similar efforts are urgently needed for influenza and RSV. Despite the unprecedented depth of metabolic and immunological profiling conducted for SARS-CoV-2, comparable multi-omics datasets and therapeutic trials remain limited for other respiratory viruses. This disparity is especially concerning given recent epidemiological trends. For example, during the 2023–2024 season in the United States, influenza was responsible for approximately 40 million illnesses, 470,000 hospitalizations, and 28,000 deaths, which now rival or exceed those associated with COVID-19 in the same period [392]. Similarly, RSV is increasingly recognized as a serious pathogen in older adults, causing 60,000–160,000 hospitalizations and 6,000–10,000 deaths annually in individuals over 65 [393].

Given this context, future research should build upon the methodologies and insights developed during the COVID-19 pandemic to study influenza and RSV with equivalent rigor. Priority areas include expanding the application of multi-omics to uncover metabolic vulnerabilities, conducting randomized clinical trials to validate metabolically guided therapies, and integrating machine learning models for patient stratification and prediction of disease trajectories. Additionally, systems biology approaches that combine omics data with clinical phenotyping will be essential to unravel the complex interplay between viral infection, host metabolism, and immune response.

In conclusion, metabolic reprogramming is a central and dynamic aspect of respiratory viral pathogenesis. Leveraging this knowledge deepens our mechanistic understanding and opens new avenues for diagnosis, risk stratification, and therapeutic intervention. Bridging the current research gap between SARS-CoV-2 and other respiratory viruses like influenza and RSV is essential to ensure that all patients benefit from the advances in precision medicine that are now within reach.

## 10. Caveats and Limitations of the Present Review

This review focuses on the metabolic reprogramming of host cells induced by respiratory viral infections, highlighting SARS-CoV-2, influenza virus, and respiratory syncytial virus. While we discuss some interactions between metabolism and immune responses, a comprehensive treatment of immunometabolism—the field studying how metabolic pathways regulate immune cell function and influence infection outcomes—was beyond the scope of this work. Immunometabolic processes have a critical influence on antiviral immunity, inflammation, and disease progression, representing a rapidly evolving area of research. For readers interested in a deeper exploration of immunometabolism in viral infections, we recommend the following recent reviews [394,395,396], which provide extensive analyses of these mechanisms and their potential therapeutic implications.

Additionally, it is essential to recognize that many metabolic alterations described herein are not exclusive to these respiratory viral infections. Several studies have reported similar metabolic dysregulations, including enhanced glycolysis, mitochondrial dysfunction, and oxidative stress, in bacterial infections such as pneumonia, as well as systemic viral diseases like hepatitis [397,398,399,400]. These findings suggest that some metabolic signatures may reflect generalized host responses to inflammation, hypoxia, and immune activation rather than pathogen-specific effects. Consequently, distinguishing whether observed metabolic changes in plasma or serum samples are directly attributable to specific viral infections or secondary to overlapping clinical manifestations and inflammatory states remains challenging. This limitation should be considered when interpreting metabolic biomarkers, highlighting the need for comparative studies across a broader range of infectious diseases.

## Figures and Tables

**Table 1:** Comparison of lipid metabolism in SARS-CoV-2, influenza virus, and RSV infections.

Feature	SARS-CoV-2	Influenza Virus	RSV
Lipid rafts in viral entry	Spike protein binding to ACE2 and entry.	Mainly clathrin-dependent endocytosis involving lipid rafts.	May bind to heparan sulfate and use rafts for attachment and fusion.
Fatty acid synthesis	Increased FASN	Increased FASN	Increased FASN
LD involvement	Induces LD accumulation for immune evasion and viral assembly.	Depletes or degrades LD via mTOR activation to fuel viral replication; inhibits LD biogenesis.	Disperses and reduces LD content; mechanisms less well characterized, but likely interfere with LD-associated immune signaling.
β-oxidation alterations	Inhibits β-oxidation, causing lipid accumulation.	Suppresses β-oxidation; shifts toward lipogenesis.	Mitochondrial dysfunction affects β-oxidation; less studied.
Cholesterol dependency	Cholesterol metabolism dysregulation.	Cholesterol metabolism dysregulation.	Not well characterized.
Lipoprotein metabolism	Decreased HDL; increased VLDL and LDL.	Transient decreased HDL and increased LDL.	Not well characterized.

FASN: Fatty acid synthase; HDL: High-density lipoproteins; LD: Lipid droplets; LDL: Low-density lipoproteins; mTOR: Mechanistic target of rapamycin; RSV: Respiratory syncytial virus; VLDL: Very low-density lipoproteins.

**Table 2:** Effects of respiratory viruses on energy metabolism and mitochondrial dynamics.

Feature	SARS-CoV-2	Influenza Virus	RSV
Glycolysis and OXPHOS	Increased glycolysis (Warburg-like shift); decreased OXPHOS.	Increased glycolysis (Warburg-like shift); decreased OXPHOS.	Increased glycolysis (Warburg-like shift); decreased OXPHOS.
Mitochondrial dysfunction	Altered morphology, decreased function; increased ROS; inhibited MAVS; activated NLRP3.	Altered morphology, decreased function; increased ROS; activated NLRP3.	Altered morphology, decreased function; increased ROS.
Bioenergetic failure	Decreased ATP production; impaired mitochondrial respiration; shift to anaerobic metabolism.	Decreased ATP production; impaired mitochondrial respiration; shift to anaerobic metabolism.	Decreased ATP production; impaired mitochondrial respiration; shift to anaerobic metabolism.
Cellular metabolic sensors	HIF-1α activation; AMPK inhibition; altered mTOR and PI3K–Akt signaling.	HIF-1α and PI3K–Akt–mTOR axis activation; energy reprogramming.	mTOR and PI3K–Akt upregulation.

AMPK: AMP-activated protein kinase; ATP: Adenosine triphosphate; HIF-1α: Hypoxia-inducible factor 1α; MAVS: Mitochondrial antiviral-signaling protein; mTOR: Mechanistic target of rapamycin; NLRP3: NOD-, LRR-, and pyrin domain-containing protein 3; PI3K–Akt: Phosphoinositide 3 kinase-Akt; ROS: Reactive oxygen species; RSV: Respiratory syncytial virus.

**Table 3:** Main alterations in amino acid and nucleotide metabolism in SARS-CoV-2, influenza virus, and RSV infections.

Pathway	SARS-CoV-2	Influenza Virus	RSV
Glutamine/glutamate	Increased glutaminolysis	Increased glutaminolysis	Increased glutaminolysis
Arginine metabolism	Arginine depletion; arginase upregulation; decreased NO production.	Arginine depletion; arginase upregulation; decreased NO production.	Arginine depletion; arginase upregulation; decreased NO production.
Tryptophan–kynurenine	Strongly activated; increased IDO1 activity; correlates with severity	Moderate activation; role in immune modulation	Upregulated IDO1 in severe cases; less characterized
Cysteine metabolism	Decreased cysteine availability.	Decreased cysteine availability.	Decreased cysteine availability.
Nucleotide metabolism	Increased biosynthesis and salvage pathways	Increased biosynthesis and salvage pathways	Increased biosynthesis and salvage pathways

IDO1: Indoleamine 2,3-dioxygenase 1; GSH: Glutathione; RSV: Respiratory syncytial virus.

**Table 4:** Main alterations in endogenous antioxidant systems during SARS-CoV-2, influenza virus, and RSV infections.

Pathway	SARS-CoV-2	Influenza Virus	RSV
Glutathione	Increased glutahione oxidation.	Transient increased glutahione oxidation.	Increased glutahione oxidation.
Superoxide dismutase	Decreased activity in plasma and tissues; associated with disease severity.	Variable: Decreased activity in severe disease; oxidative burden overwhelms the enzyme.	Variable: Decreased activity in severe disease; oxidative burden overwhelms the enzyme.
Catalase	Decreased activity.	Decreased activity.	Decreased activity.
Paraoxonase 1	Markedly decreased activity.	Less characterized.	Less characterized.

RSV: Respiratory syncytial virus.

**Table 5:** Candidate drugs targeting virus-induced metabolic alterations in respiratory infections.

Metabolic Pathway	Proposed Drug	Targeted Virus
Viral entry	Camostat mesylate Statins Chloroquine Amantadine, rimantadine DAS-181 Palivizumab Other protein F inhibitors	SARS-CoV-2 Influenza SARS-CoV-2 Influenza Influenza RSV RSV
Lipid metabolism	Orlistat Statins Omega-3 fatty acids	SARS-CoV-2, Influenza, RSV SARS-CoV-2 SARS-CoV-2
Glycolysis	2-deoxy-D-glucose KAN0438757 3PO	SARS-CoV-2 Influenza Influenza
Mitochondrial dynamics Bioenergetics	Mdivi 1 Melatonin Coenzyme Q10 Metformin Szeto-Schiller peptides	Other viruses SARS-CoV-2, Influenza, RSV Not tested on viruses SARS-CoV-2, Influenza Not tested on viruses
Endogenous antioxidants	N-acetylcysteine Melatonin Tempol Ebselen	SARS-CoV-2, Influenza SARS-CoV-2, Influenza, RSV SARS-CoV-2 SARS-CoV-2
Kynurenine pathway	Epacadostat, navoximod	Not tested on viruses

3PO: 3-(3-pyridinyl)-1-(4-pyridinyl)-2-propen-1-one; Mdivi 1: Mitochondrial division inhibitor 1; RSV: Respiratory syncytial virus.

## Data Availability

No new data were created or analyzed in this study. Data sharing is not applicable to this article.

## Abbreviations

ACE2	Angiotensin-converting enzyme 2
AMPK	AMP-activated protein kinase
ATP	Adenosine triphosphate
AUC	Area under the curve
AhR	Aryl hydrocarbon receptor
CAR 8:0	O-octanoyl-L-carnitine
CAT	Catalase
COVID-19	Coronavirus disease 2019
CPT	Carnitine palmitoyltransferases
CCL2	Chemokine (C-C motif) ligand 2
DGAT	Diacylglycerol O-acyltransferase
Drp1	Dynamin-related protein 1
FASN	Fatty acid synthase
GADPH	Glyceraldehyde 3-phosphate dehydrogenase
GC	Gas chromatography
GLUT	Glucose transporter
GSH	Glutathione
HDL	High-density lipoproteins
HETE	Hydroxyeicosatetraenoic acid
HIF-1α	Hypoxia-inducible factor 1-α
HODE	Hydroxyoctadecadienoic acid
IDO1	Indoleamine 2,3-dioxygenase 1
IFN	Interferon
IL	Interleukin
Kyn/Trp	Kynurenine/tryptophan
LC	Liquid chromatography
LD	Lipid droplets
LDL	Low-density lipoproteins
LLPS	Liquid–liquid phase separation
LPC	Lysophosphatidylcholine
LPE	Lysophosphatidylethanolamine
MAVS	Mitochondrial antiviral-signaling protein
MCP-1	Monocyte chemoattractant protein-1
Mdivi-1	Mitochondrial division inhibitor 1
MS	Mass spectrometry
mTOR	Mechanistic target of rapamycin
NAC	N-acetylcysteine
NADH	Reduced nicotinamide adenine dinucleotide
NADPH	Reduced nicotinamide adenine dinucleotide phosphate
NF-κB	Nuclear factor kappa B
NLRP3	NOD-, LRR-, and pyrin domain-containing protein 3
NMR	Nuclear magnetic resonance
NOX	NADPH oxidase
NRF2	Nuclear factor erythroid 2-related factor 2
OXPHOS	Oxidative phosphorylation
PGC-1α	Peroxisome proliferator-activated receptor-γ coactivator 1-α
PC	Phosphatidylcholine
PFKFB3	6-phosphofructo-2-kinase/fructose-2,6-biphosphatase 3
PI	Phosphatidylinositol
PI3K	Phosphoinositide 3-kinase
3PO	3-(3-pyridinyl)-1-(4-pyridinyl)-2-propen-1-one
PON1	Paraoxonase-1
ROC	Receiver operating characteristic
ROS	Reactive oxygen species
RSV	Respiratory syncytial virus
SARS-CoV-2	Severe acute respiratory syndrome coronavirus 2
SOD	Superoxide dismutase
SREBP	Sterol regulatory element-binding proteins
SREBP2-LDLR	Sterol regulatory element-binding protein 2 and low-density lipoprotein receptor
TMPRSS2	Transmembrane protease, serine 2
TNF-α	Tumor necrosis factor-α
VLDL	Very low-density lipoproteins

## References

[1] Safiri S., Mahmoodpoor A., Kolahi A.A., Nejadghaderi S.A., Sullman M.J.M., Mansournia M.A., Ansarin K., Collins G.S., Kaufman J.S., Abdollahi M. (2023). Global burden of lower respiratory infections during the last three decades. Front. Public Health.

[2] Gravenstein S. (2025). Foreword: Prevention of COVID-19, influenza, and respiratory syncytial virus in at-risk populations. Infect. Dis. Ther..

[3] Hanage W.P., Schaffner W. (2025). Burden of acute respiratory infections caused by influenza virus, respiratory syncytial virus, and SARS-CoV-2 with consideration of older adults: A narrative review. Infect. Dis. Ther..

[4] Kleinehr J., Wilden J.J., Boergeling Y., Ludwig S., Hrincius E.R. (2021). Metabolic modifications by common respiratory viruses and their potential as new antiviral targets. Viruses.

[5] Allen C.N.S., Arjona S.P., Santerre M., Sawaya B.E. (2022). Hallmarks of metabolic reprogramming and their role in viral pathogenesis. Viruses.

[6] Shahpar A., Sofiani V.H., Nezhad N.Z., Charostad M., Ghaderi R., Farsiu N., Kiskani A.K., Pezeshki S., Nakhaie M. (2024). A narrative review: Exploring viral-induced malignancies through the lens of dysregulated cellular metabolism and glucose transporters. BMC Cancer.

[7] Konaklieva M.I., Plotkin B.J. (2024). Targeting host-specific metabolic pathways-opportunities and challenges for anti-infective therapy. Front. Mol. Biosci..

[8] Zumla A., Rao M., Wallis R.S., Kaufmann S.H., Rustomjee R., Mwaba P., Vilaplana C., Yeboah-Manu D., Chakaya J., Ippolito G. (2016). Host-directed therapies for infectious diseases: Current status, recent progress, and future prospects. Lancet Infect. Dis..

[9] Palmer C.S. (2022). Innate metabolic responses against viral infections. Nat. Metab..

[10] Mazzon M., Mercer J. (2014). Lipid interactions during virus entry and infection. Cell. Microbiol..

[11] Waheed A.A., Freed E.O. (2010). The role of lipids in retrovirus replication. Viruses.

[12] Simons K., Sampaio J.L. (2011). Membrane organization and lipid rafts. Cold Spring Harb. Perspect. Biol..

[13] Pike L.J. (2006). Rafts defined: A report on the Keystone Symposium on Lipid Rafts and Cell Function. J. Lipid Res..

[14] Ripa I., Andreu S., López-Guerrero J.A., Bello-Morales R. (2021). Membrane rafts: Portals for viral entry. Front. Microbiol..

[15] Lorizate M., Kräusslich H.G. (2011). Role of lipids in virus replication. Cold Spring Harb. Perspect. Biol..

[16] Marsh M., Helenius A. (2006). Virus entry: Open sesame. Cell.

[17] Glende J., Schwegmann-Wessels C., Al-Falah M., Pfefferle S., Qu X., Deng H., Drosten C., Naim H.Y., Herrler G. (2008). Importance of cholesterol-rich membrane microdomains in the interaction of the S protein of SARS-coronavirus with the cellular receptor angiotensin-converting enzyme 2. Virology.

[18] Lu Y., Liu D.X., Tam J.P. (2008). Lipid rafts are involved in SARS-CoV entry into Vero E6 cells. Biochem. Biophys. Res. Commun..

[19] Hoffmann M., Kleine-Weber H., Schroeder S., Krüger N., Herrler T., Erichsen S., Schiergens T.S., Herrler G., Wu N.H., Nitsche A. (2020). SARS-CoV-2 cell entry depends on ACE2 and TMPRSS2 and is blocked by a clinically proven protease inhibitor. Cell.

[20] Chakraborty S., Veettil M.V., Bottero V., Chandran B. (2012). Kaposi’s sarcoma-associated herpesvirus interacts with EphrinA2 receptor to amplify signaling essential for productive infection. Proc. Natl. Acad. Sci. USA.

[21] Jiang Y., Liu S., Shen S., Guo H., Huang H., Wei W. (2020). Methyl-β-cyclodextrin inhibits EV-D68 virus entry by perturbing the accumulation of virus particles and ICAM-5 in lipid rafts. Antivir. Res..

[22] Roncato R., Angelini J., Pani A., Talotta R. (2022). Lipid rafts as viral entry routes and immune platforms: A double-edged sword in SARS-CoV-2 infection?. Biochim. Biophys. Acta Mol. Cell Biol. Lipids.

[23] Bukrinsky M.I., Mukhamedova N., Sviridov D. (2020). Lipid rafts and pathogens: The art of deception and exploitation. J. Lipid Res..

[24] Song M.S., Lee D.K., Lee C.Y., Park S.C., Yang J. (2024). Host subcellular organelles: Targets of viral manipulation. Int. J. Mol. Sci..

[25] Rawat S.S., Viard M., Gallo S.A., Rein A., Blumenthal R., Puri A. (2003). Modulation of entry of enveloped viruses by cholesterol and sphingolipids. Mol. Membr. Biol..

[26] Harrison S.C. (2015). Viral membrane fusion. Virology.

[27] Barrett C.T., Dutch R.E. (2020). Viral membrane fusion and the transmembrane domain. Viruses.

[28] Basturea G. (2019). Endocytosis. Mater. Methods.

[29] Kaksonen M., Roux A. (2018). Mechanisms of clathrin-mediated endocytosis. Nat. Rev. Mol. Cell Biol..

[30] Mayor S., Parton R.G., Donaldson J.G. (2014). Clathrin-independent pathways of endocytosis. Cold Spring Harb. Perspect. Biol..

[31] Sandvig K., Kavaliauskiene S., Skotland T. (2018). Clathrin-independent endocytosis: An increasing degree of complexity. Histochem. Cell Biol..

[32] Shafaq-Zadah M., Dransart E., Johannes L. (2020). Clathrin-independent endocytosis, retrograde trafficking, and cell polarity. Curr. Opin. Cell Biol..

[33] Ruzzi F., Cappello C., Semprini M.S., Scalambra L., Angelicola S., Pittino O.M., Landuzzi L., Palladini A., Nanni P., Lollini P.L. (2024). Lipid rafts, caveolae, and epidermal growth factor receptor family: Friends or foes?. Cell Commun. Signal..

[34] McMahon K.A., Zajicek H., Li W.P., Peyton M.J., Minna J.D., Hernandez V.J., Luby-Phelps K., Anderson R.G. (2009). SRBC/cavin-3 is a caveolin adapter protein that regulates caveolae function. EMBO J..

[35] Kovtun O., Tillu V.A., Ariotti N., Parton R.G., Collins B.M. (2015). Cavin family proteins and the assembly of caveolae. J. Cell Sci..

[36] Vicinanza M., D’angelo G., Di Campli A., De Matteis M.A. (2008). Function and dysfunction of the PI system in membrane trafficking. EMBO J..

[37] Vanhaesebroeck B., Stephens L., Hawkins P. (2012). PI3K signalling: The path to discovery and understanding. Nat. Rev. Mol. Cell Biol..

[38] Das S., Chakraborty S., Basu A. (2010). Critical role of lipid rafts in virus entry and activation of phosphoinositide 3′ kinase/Akt signaling during early stages of Japanese encephalitis virus infection in neural stem/progenitor cells. J. Neurochem..

[39] Bohdanowicz M., Grinstein S. (2013). Role of phospholipids in endocytosis, phagocytosis, and macropinocytosis. Physiol. Rev..

[40] Marjuki H., Gornitzky A., Marathe B.M., Ilyushina N.A., Aldridge J.R., Desai G., Webby R.J., Webster R.G. (2011). Influenza A virus-induced early activation of ERK and PI3K mediates V-ATPase-dependent intracellular pH change required for fusion. Cell. Microbiol..

[41] Fujioka Y., Tsuda M., Hattori T., Sasaki J., Sasaki T., Miyazaki T., Ohba Y. (2011). The Ras-PI3K signaling pathway is involved in clathrin-independent endocytosis and the internalization of influenza viruses. PLoS ONE.

[42] Palacios-Rápalo S.N., De Jesús-González L.A., Cordero-Rivera C.D., Farfan-Morales C.N., Osuna-Ramos J.F., Martínez-Mier G., Quistián-Galván J., Muñoz-Pérez A., Bernal-Dolores V., Del Ángel R.M. (2021). Cholesterol-rich lipid rafts as platforms for SARS-CoV-2 entry. Front. Immunol..

[43] El Khoury M., Naim H.Y. (2024). Lipid rafts disruption by statins negatively impacts the interaction between SARS-CoV-2 S1 subunit and ACE2 in intestinal epithelial cells. Front. Microbiol..

[44] Zhang Y., Whittaker G.R. (2014). Influenza entry pathways in polarized MDCK cells. Biochem. Biophys. Res. Commun..

[45] Bolland W., Marechal I., Petiot C., Porrot F., Guivel-Benhassine F., Brelot A., Casartelli N., Schwartz O., Buchrieser J. (2025). SARS-CoV-2 entry and fusion are independent of ACE2 localization to lipid rafts. J. Virol..

[46] Rossman J.S., Leser G.P., Lamb R.A. (2012). Filamentous influenza virus enters cells via macropinocytosis. J. Virol..

[47] de Vries E., Tscherne D.M., Wienholts M.J., Cobos-Jiménez V., Scholte F., García-Sastre A., Rottier P.J., de Haan C.A. (2011). Dissection of the influenza A virus endocytic routes reveals macropinocytosis as an alternative entry pathway. PLoS Pathog..

[48] Verma D.K., Gupta D., Lal S.K. (2018). Host lipid rafts play a major role in binding and endocytosis of Influenza A virus. Viruses.

[49] Fleming E.H., Kolokoltsov A.A., Davey R.A., Nichols J.E., Roberts N.J. (2006). Respiratory syncytial virus F envelope protein associates with lipid rafts without a requirement for other virus proteins. J. Virol..

[50] Shaikh F.Y., Crowe J.E. (2013). Molecular mechanisms driving respiratory syncytial virus assembly. Future Microbiol..

[51] Chandel N.S. (2021). Lipid metabolism. Cold Spring Harb. Perspect. Biol..

[52] Chu J., Xing C., Du Y., Duan T., Liu S., Zhang P., Cheng C., Henley J., Liu X., Qian C. (2021). Pharmacological inhibition of fatty acid synthesis blocks SARS-CoV-2 replication. Nat. Metab..

[53] Williams C.G., Jureka A.S., Silvas J.A., Nicolini A.M., Chvatal S.A., Carlson-Stevermer J., Oki J., Holden K., Basler C.F. (2021). Inhibitors of VPS34 and fatty-acid metabolism suppress SARS-CoV-2 replication. Cell Rep..

[54] Limsuwat N., Boonarkart C., Phakaratsakul S., Suptawiwat O., Auewarakul P. (2020). Influence of cellular lipid content on influenza A virus replication. Arch. Virol..

[55] Ohol Y.M., Wang Z., Kemble G., Duke G. (2015). Direct inhibition of cellular fatty acid synthase impairs replication of respiratory syncytial virus and other respiratory viruses. PLoS ONE.

[56] Aliyari S.R., Ghaffari A.A., Pernet O., Parvatiyar K., Wang Y., Gerami H., Tong A.J., Vergnes L., Takallou A., Zhang A. (2022). Suppressing fatty acid synthase by type I interferon and chemical inhibitors as a broad spectrum anti-viral strategy against SARS-CoV-2. Acta Pharm. Sin. B.

[57] Wölk M., Fedorova M. (2024). The lipid droplet lipidome. FEBS Lett..

[58] Xu Y., Mak H.Y., Lukmantara I., Li Y.E., Hoehn K.L., Huang X., Du X., Yang H. (2019). CDP-DAG synthase 1 and 2 regulate lipid droplet growth through distinct mechanisms. J. Biol. Chem..

[59] Monks J., Orlicky D.J., Libby A.E., Dzieciatkowska M., Ladinsky M.S., McManaman J.L. (2022). Perilipin-2 promotes lipid droplet-plasma membrane interactions that facilitate apocrine lipid secretion in secretory epithelial cells of the mouse mammary gland. Front. Cell Dev. Biol..

[60] Dias S.S.G., Soares V.C., Ferreira A.C., Sacramento C.Q., Fintelman-Rodrigues N., Temerozo J.R., Teixeira L., Nunes da Silva M.A., Barreto E., Mattos M. (2020). Lipid droplets fuel SARS-CoV-2 replication and production of inflammatory mediators. PLoS Pathog..

[61] Chawla K., Subramanian G., Rahman T., Fan S., Chakravarty S., Gujja S., Demchak H., Chakravarti R., Chattopadhyay S. (2022). Autophagy in virus infection: A race between host immune response and viral antagonism. Immuno.

[62] Herrera-Moro Huitron L., De Jesús-González L.A., Martínez-Castillo M., Ulloa-Aguilar J.M., Cabello-Gutierrez C., Helguera-Repetto C., Garcia-Cordero J., León Juárez M. (2023). Multifaceted nature of lipid droplets in viral interactions and pathogenesis. Microorganisms.

[63] Kuss-Duerkop S.K., Wang J., Mena I., White K., Metreveli G., Sakthivel R., Mata M.A., Muñoz-Moreno R., Chen X., Krammer F. (2017). Influenza virus differentially activates mTORC1 and mTORC2 signaling to maximize late stage replication. PLoS Pathog..

[64] Dai P., Tang Z., Qi M., Liu D., Bajinka O., Tan Y. (2022). Dispersion and utilization of lipid droplets mediates respiratory syncytial virus-induced airway hyperresponsiveness. Pediatr. Allergy Immunol..

[65] Cheung W., Gill M., Esposito A., Kaminski C.F., Courousse N., Chwetzoff S., Trugnan G., Keshavan N., Lever A., Desselberger U. (2010). Rotaviruses associate with cellular lipid droplet components to replicate in viroplasms, and compounds disrupting or blocking lipid droplets inhibit viroplasm formation and viral replication. J. Virol..

[66] Longo N., Frigeni M., Pasquali M. (2016). Carnitine transport and fatty acid oxidation. Biochim. Biophys. Acta.

[67] Talley J.T., Mohiuddin S.S. (2025). Biochemistry, Fatty Acid Oxidation. StatPearls [Internet].

[68] Tanner L.B., Chng C., Guan X.L., Lei Z., Rozen S.G., Wenk M.R. (2014). Lipidomics identifies a requirement for peroxisomal function during influenza virus replication. J. Lipid Res..

[69] Andrade Silva M., da Silva A.R.P.A., do Amaral M.A., Fragas M.G., Câmara N.O.S. (2021). Metabolic alterations in SARS-CoV-2 infection and its implication in kidney dysfunction. Front. Physiol..

[70] Keshavarz M., Solaymani-Mohammadi F., Namdari H., Arjeini Y., Mousavi M.J., Rezaei F. (2020). Metabolic host response and therapeutic approaches to influenza infection. Cell. Mol. Biol. Lett..

[71] Pérez S.E., Gooz M., Maldonado E.N. (2024). Mitochondrial dysfunction and metabolic disturbances induced by viral infections. Cells.

[72] Feingold K.R., Feingold K.R., Anawalt B., Blackman M.R., Boyce A., Chrousos G., Corpas E., de Herder W.W., Dhatariya K., Dungan K., Hofland J. (2024). Introduction to Lipids and Lipoproteins. Endotext [Internet].

[73] Navab M., Reddy S.T., Van Lenten B.J., Fogelman A.M. (2011). HDL and cardiovascular disease: Atherogenic and atheroprotective mechanisms. Nat. Rev. Cardiol..

[74] Catapano A.L., Pirillo A., Bonacina F., Norata G.D. (2014). HDL in innate and adaptive immunity. Cardiovasc. Res..

[75] Camps J., Iftimie S., García-Heredia A., Castro A., Joven J. (2017). Paraoxonases and infectious diseases. Clin. Biochem..

[76] Hima Bindu G., Rao V.S., Kakkar V.V. (2011). Friend turns foe: Transformation of anti-inflammatory HDL to proinflammatory HDL during acute-phase response. Cholesterol.

[77] Camps J., Castañé H., Rodríguez-Tomàs E., Baiges-Gaya G., Hernández-Aguilera A., Arenas M., Iftimie S., Joven J. (2021). On the role of paraoxonase-1 and chemokine ligand 2 (C-C motif) in metabolic alterations linked to inflammation and disease. A 2021 update. Biomolecules.

[78] Camps J., Iftimie S., Arenas M., Castañé H., Jiménez-Franco A., Castro A., Joven J. (2023). Paraoxonase-1: How a xenobiotic detoxifying enzyme has become an actor in the pathophysiology of infectious diseases and cancer. Chem. Biol. Interact..

[79] Mallol R., Amigó N., Rodríguez M.A., Heras M., Vinaixa M., Plana N., Rock E., Ribalta J., Yanes O., Masana L. (2015). Liposcale: A novel advanced lipoprotein test based on 2D diffusion-ordered 1H NMR spectroscopy. J. Lipid Res..

[80] Ballout R.A., Kong H., Sampson M., Otvos J.D., Cox A.L., Agbor-Enoh S., Remaley A.T. (2021). The NIH lipo-COVID study: A pilot NMR investigation of lipoprotein subfractions and other metabolites in patients with severe COVID-19. Biomedicines.

[81] Lodge S., Nitschke P., Kimhofer T., Coudert J.D., Begum S., Bong S.H., Richards T., Edgar D., Raby E., Spraul M. (2021). NMR spectroscopic windows on the systemic effects of SARS-CoV-2 infection on plasma lipoproteins and metabolites in relation to circulating cytokines. J. Proteome Res..

[82] Schmelter F., Föh B., Mallagaray A., Rahmöller J., Ehlers M., Lehrian S., von Kopylow V., Künsting I., Lixenfeld A.S., Martin E. (2021). Metabolic and lipidomic markers differentiate COVID-19 from non-hospitalized and other intensive care patients. Front. Mol. Biosci..

[83] Rössler T., Berezhnoy G., Singh Y., Cannet C., Reinsperger T., Schäfer H., Spraul M., Kneilling M., Merle U., Trautwein C. (2022). Quantitative serum NMR spectroscopy stratifies COVID-19 patients and sheds light on interfaces of host metabolism and the immune response with cytokines and clinical parameters. Metabolites.

[84] Iftimie S., Amigó N., Martínez-Micaelo N., López-Azcona A.F., Martínez-Navidad C., Castañé H., Jiménez-Franco A., Ribalta J., Parra S., Castro A. (2024). Differential analysis of lipoprotein and glycoprotein profiles in bacterial infections and COVID-19 using proton nuclear magnetic resonance and machine learning. Heliyon.

[85] Van Lenten B.J., Wagner A.C., Anantharamaiah G.M., Garber D.W., Fishbein M.C., Adhikary L., Nayak D.P., Hama S., Navab M., Fogelman A.M. (2002). Influenza infection promotes macrophage traffic into arteries of mice that is prevented by D-4F, an apolipoprotein A-I mimetic peptide. Circulation.

[86] Heinzl M.W., Freudenthaler M., Fellinger P., Kolenchery L., Resl M., Klammer C., Obendorf F., Schinagl L., Berger T., Egger M. (2024). High-density lipoprotein predicts intrahospital mortality in influenza. J. Clin. Med..

[87] Chen L., Zhang J., Xu W., Chen J., Tang Y., Xiong S., Li Y., Zhang H., Li M., Liu Z. (2024). Cholesterol-rich lysosomes induced by respiratory syncytial virus promote viral replication by blocking autophagy flux. Nat. Commun..

[88] Doyle A., Goodson B.A., Kolaczkowski O.M., Liu R., Jia J., Wang H., Han X., Ye C., Bradfute S.B., Kell A.M. (2024). Manipulation of host cholesterol by SARS-CoV-2. bioRxiv.

[89] Li Y.J., Chen C.Y., Yang J.H., Chiu Y.F. (2022). Modulating cholesterol-rich lipid rafts to disrupt influenza A virus infection. Front. Immunol..

[90] Carter T., Iqbal M. (2024). The influenza A virus replication cycle: A comprehensive review. Viruses.

[91] Liu H., Wang S., Wang J., Guo X., Song Y., Fu K., Gao Z., Liu D., He W., Yang L.L. (2025). Energy metabolism in health and diseases. Signal Transduct. Target. Ther..

[92] Singh S.P., Amar S., Gehlot P., Patra S.K., Kanwar N., Kanwal A. (2021). Mitochondrial modulations, autophagy pathways shifts in viral infections: Consequences of COVID-19. Int. J. Mol. Sci..

[93] Sanchez E.L., Lagunoff M. (2015). Viral activation of cellular metabolism. Virology.

[94] Singh S., Singh P.K., Suhail H., Arumugaswami V., Pellett P.E., Giri S., Kumar A. (2020). AMP-activated protein kinase restricts Zika virus replication in endothelial cells by potentiating innate antiviral responses and inhibiting glycolysis. J. Immunol. Baltim. Md..

[95] Pouysségur J., Marchiq I., Parks S.K., Durivault J., Ždralević M., Vucetic M. (2022). ‘Warburg effect’ controls tumor growth, bacterial, viral infections and immunity-Genetic deconstruction and therapeutic perspectives. Semin. Cancer Biol..

[96] Codo A.C., Davanzo G.G., Monteiro L.B., de Souza G.F., Muraro S.P., Virgilio-da-Silva J.V., Prodonoff J.S., Carregari V.C., de Biagi Junior C.A.O., Crunfli F. (2020). Elevated glucose levels favor SARS-CoV-2 infection and monocyte response through a HIF-1α/glycolysis-dependent Axis. Cell Metab..

[97] Santos A.F., Póvoa P., Paixão P., Mendonça A., Taborda-Barata L. (2021). Changes in glycolytic pathway in SARS-COV 2 infection and their importance in understanding the severity of COVID-19. Front. Chem..

[98] Guarnieri J.W., Lie T., Albrecht Y.E.S., Hewin P., Jurado K.A., Widjaja G.A., Zhu Y., McManus M.J., Kilbaugh T.J., Keith K. (2024). Mitochondrial antioxidants abate SARS-COV-2 pathology in mice. Proc. Natl. Acad. Sci. USA.

[99] Guo X., Zhu Z., Zhang W., Meng X., Zhu Y., Han P., Zhou X., Hu Y., Wang R. (2017). Nuclear translocation of HIF-1α induced by influenza A (H1N1) infection is critical to the production of proinflammatory cytokines. Emerg. Microbes Infect..

[100] Meng X., Zhu Y., Yang W., Zhang J., Jin W., Tian R., Yang Z., Wang R. (2024). HIF-1α promotes virus replication and cytokine storm in H1N1 virus-induced severe pneumonia through cellular metabolic reprogramming. Virol. Sin..

[101] Zhao C., Chen J., Cheng L., Xu K., Yang Y., Su X. (2020). Deficiency of HIF-1α enhances influenza A virus replication by promoting autophagy in alveolar type II epithelial cells. Emerg. Microbes Infect..

[102] Reyes A., Duarte L.F., Farías M.A., Tognarelli E., Kalergis A.M., Bueno S.M., González P.A. (2021). Impact of hypoxia over human viral infections and key cellular processes. Int. J. Mol. Sci..

[103] Wang R., Zhu Y., Ren C., Yang S., Tian S., Chen H., Jin M., Zhou H. (2021). Influenza A virus protein PB1-F2 impairs innate immunity by inducing mitophagy. Autophagy.

[104] Chen L.F., Cai J.X., Zhang J.J., Tang Y.J., Chen J.Y., Xiong S., Li Y.L., Zhang H., Liu Z., Li M.M. (2023). Respiratory syncytial virus co-opts hypoxia-inducible factor-1α-mediated glycolysis to favor the production of infectious virus. mBio.

[105] Kahan S.M., Wherry E.J., Zajac A.J. (2015). T cell exhaustion during persistent viral infections. Virology.

[106] Wu L., Yan Z., Jiang Y., Chen Y., Du J., Guo L., Xu J., Luo Z., Liu Y. (2023). Metabolic regulation of dendritic cell activation and immune function during inflammation. Front. Immunol..

[107] Stefano G.B., Weissenberger S., Ptacek R., Anders M., Raboch J., Büttiker P. (2024). Viruses and mitochondrial dysfunction in neurodegeneration and cognition: An evolutionary perspective. Cell. Mol. Neurobiol..

[108] Gay L., Desquiret-Dumas V., Nagot N., Rapenne C., Van de Perre P., Reynier P., Molès J.P. (2024). Long-term persistence of mitochondrial dysfunctions after viral infections and antiviral therapies: A review of mechanisms involved. J. Med. Virol..

[109] Elesela S., Lukacs N.W. (2021). Role of mitochondria in viral infections. Life.

[110] Purandare N., Ghosalkar E., Grossman L.I., Aras S. (2023). Mitochondrial oxidative phosphorylation in viral infections. Viruses.

[111] Guarnieri J.W., Dybas J.M., Fazelinia H., Kim M.S., Frere J., Zhang Y., Soto Albrecht Y., Murdock D.G., Angelin A., Singh L.N. (2023). Core mitochondrial genes are down-regulated during SARS-CoV-2 infection of rodent and human hosts. Sci. Transl. Med..

[112] Miller B., Silverstein A., Flores M., Cao K., Kumagai H., Mehta H.H., Yen K., Kim S.J., Cohen P. (2021). Host mitochondrial transcriptome response to SARS-CoV-2 in multiple cell models and clinical samples. Sci. Rep..

[113] Westermann B. (2010). Mitochondrial fusion and fission in cell life and death. Nat. Rev. Mol. Cell Biol..

[114] Mehmood T., Nasir Q., Younis I., Muanprasat C. (2025). Inhibition of mitochondrial dynamics by mitochondrial division inhibitor-1 suppresses cell migration and metastatic markers in colorectal cancer HCT116 cells. J. Exp. Pharmacol..

[115] Barbier V., Lang D., Valois S., Rothman A.L., Medin C.L. (2017). Dengue virus induces mitochondrial elongation through impairment of Drp1-triggered mitochondrial fission. Virology.

[116] Archer S.L., Dasgupta A., Chen K.H., Wu D., Baid K., Mamatis J.E., Gonzalez V., Read A., Bentley R.E., Martin A.Y. (2022). SARS-CoV-2 mitochondriopathy in COVID-19 pneumonia exacerbates hypoxemia. Redox Biol..

[117] Gómez-Delgado I., López-Pastor A.R., González-Jiménez A., Ramos-Acosta C., Hernández-Garate Y., Martínez-Micaelo N., Amigó N., Espino-Paisán L., Anguita E., Urcelay E. (2025). Long-term mitochondrial and metabolic impairment in lymphocytes of subjects who recovered after severe COVID-19. Cell Biol. Toxicol..

[118] Tábara L.C., Morris J.L., Prudent J. (2021). The complex dance of organelles during mitochondrial division. Trends Cell Biol..

[119] Duan X., Liu R., Lan W., Liu S. (2025). The essential role of mitochondrial dynamics in viral infections. Int. J. Mol. Sci..

[120] Vazquez C., Horner S.M. (2015). MAVS coordination of antiviral innate immunity. J. Virol..

[121] Qi Y., Yin J., Xia W., Yang S. (2025). Exploring the role of mitochondrial antiviral signaling protein in cardiac diseases. Front. Immunol..

[122] Li X., Hou P., Ma W., Wang X., Wang H., Yu Z., Chang H., Wang T., Jin S., Wang X. (2022). SARS-CoV-2 ORF10 suppresses the antiviral innate immune response by degrading MAVS through mitophagy. Cell. Mol. Immunol..

[123] Swanson K.V., Deng M., Ting J.P. (2019). The NLRP3 inflammasome: Molecular activation and regulation to therapeutics. Nat. Rev. Immunol..

[124] Hsieh L.L., Looney M., Figueroa A., Massaccesi G., Stavrakis G., Anaya E.U., D’Alessio F.R., Ordonez A.A., Pekosz A.S., DeFilippis V.R. (2024). Bystander monocytic cells drive infection-independent NLRP3 inflammasome response to SARS-CoV-2. mBio.

[125] Pandey K.P., Zhou Y. (2022). Influenza A virus infection activates NLRP3 inflammasome through trans-Golgi network dispersion. Viruses.

[126] Lee J.K., Shin O.S. (2023). Zika virus modulates mitochondrial dynamics, mitophagy, and mitochondria-derived vesicles to facilitate viral replication in trophoblast cells. Front. Immunol..

[127] Shang C., Liu Z., Zhu Y., Lu J., Ge C., Zhang C., Li N., Jin N., Li Y., Tian M. (2022). SARS-CoV-2 causes mitochondrial dysfunction and mitophagy impairment. Front. Microbiol..

[128] Bhutta M.S., Gallo E.S., Borenstein R. (2021). Multifaceted role of AMPK in viral infections. Cells.

[129] Camps J., Rodríguez-Gallego E., García-Heredia A., Triguero I., Riera-Borrull M., Hernández-Aguilera A., Luciano-Mateo F., Fernández-Arroyo S., Joven J. (2014). Paraoxonases and chemokine (C-C motif) ligand-2 in noncommunicable diseases. Adv. Clin. Chem..

[130] Hardie D.G., Ross F.A., Hawley S.A. (2012). AMPK: A nutrient and energy sensor that maintains energy homeostasis. Nat. Rev. Mol. Cell Biol..

[131] Mankouri J., Tedbury P.R., Gretton S., Hughes M.E., Griffin S.D., Dallas M.L., Green K.A., Hardie D.G., Peers C., Harris M. (2010). Enhanced hepatitis C virus genome replication and lipid accumulation mediated by inhibition of AMP-activated protein kinase. Proc. Natl. Acad. Sci. USA.

[132] Deretic V. (2021). Autophagy in inflammation, infection, and immunometabolism. Immunity.

[133] Cheng J., Wang Y., Yin L., Liang W., Zhang J., Ma C., Zhang Y., Liu B., Wang J., Zhao W. (2023). The nonstructural protein 1 of respiratory syncytial virus hijacks host mitophagy as a novel mitophagy receptor to evade the type I IFN response in HEp-2 cells. mBio.

[134] Le Sage V., Cinti A., Amorim R., Mouland A.J. (2016). Adapting the stress response: Viral subversion of the mTOR signaling pathway. Viruses.

[135] Vincent H.A., Ziehr B., Moorman N.J. (2016). Human cytomegalovirus strategies to maintain and promote mRNA translation. Viruses.

[136] Majeed S.T., Batool A., Majeed R., Bhat N.N., Zargar M.A., Andrabi K.I. (2021). mTORC1 induces eukaryotic translation initiation factor 4E interaction with TOS-S6 kinase 1 and its activation. Cell Cycle.

[137] Xie Y., Lei X., Zhao G., Guo R., Cui N. (2023). mTOR in programmed cell death and its therapeutic implications. Cytokine Growth Factor Rev..

[138] Twu W.I., Lee J.Y., Kim H., Prasad V., Cerikan B., Haselmann U., Tabata K., Bartenschlager R. (2021). Contribution of autophagy machinery factors to HCV and SARS-CoV-2 replication organelle formation. Cell Rep..

[139] Laplante M., Sabatini D.M. (2012). mTOR signaling in growth control and disease. Cell.

[140] Dunn E.F., Connor J.H. (2012). HijAkt: The PI3K/Akt pathway in virus replication and pathogenesis. Prog. Mol. Biol. Transl. Sci..

[141] Greene K.S., Choi A., Yang N., Chen M., Li R., Qiu Y., Ezzatpour S., Rojas K.S., Shen J., Wilson K.F. (2025). Glutamine metabolism is essential for coronavirus replication in host cells and in mice. J. Biol. Chem..

[142] Smallwood H.S., Duan S., Morfouace M., Rezinciuc S., Shulkin B.L., Shelat A., Zink E.E., Milasta S., Bajracharya R., Oluwaseum A.J. (2017). Targeting metabolic reprogramming by influenza infection for therapeutic intervention. Cell Rep..

[143] Lu Y., Xu S., Sun H., Shan J., Shen C., Ji J., Lin L., Xu J., Peng L., Dai C. (2023). Analysis of temporal metabolic rewiring for human respiratory syncytial virus infection by integrating metabolomics and proteomics. Metabolomics.

[144] Yoo H.C., Yu Y.C., Sung Y., Han J.M. (2020). Glutamine reliance in cell metabolism. Exp. Mol. Med..

[145] Cruzat V., Macedo Rogero M., Noel Keane K., Curi R., Newsholme P. (2018). Glutamine: Metabolism and immune function, supplementation and clinical translation. Nutrients.

[146] Wu G., Meininger C.J., McNeal C.J., Bazer F.W., Rhoads J.M. (2021). Role of L-arginine in nitric oxide synthesis and health in humans. Adv. Exp. Med. Biol..

[147] Moraes T.J. (2010). Arginase and respiratory viral infections. Open Nitric Oxide J..

[148] van den Berg M.P., Meurs H., Gosens R. (2018). Targeting arginase and nitric oxide metabolism in chronic airway diseases and their co-morbidities. Curr. Opin. Pharmacol..

[149] Dean M.J., Ochoa J.B., Sanchez-Pino M.D., Zabaleta J., Garai J., Del Valle L., Wyczechowska D., Baiamonte L.B., Philbrook P., Majumder R. (2021). Severe COVID-19 is characterized by an impaired type I interferon response and elevated levels of arginase producing granulocytic myeloid derived suppressor cells. Front. Immunol..

[150] Churiso G., Husen G., Bulbula D., Abebe L. (2022). Immunity cell responses to RSV and the role of antiviral inhibitors: A systematic review. Infect. Drug Resist..

[151] West E.E., Merle N.S., Kamiński M.M., Palacios G., Kumar D., Wang L., Bibby J.A., Overdahl K., Jarmusch A.K., Freeley S. (2023). Loss of CD4+ T cell-intrinsic arginase 1 accelerates Th1 response kinetics and reduces lung pathology during influenza infection. Immunity.

[152] Mounce B.C., Olsen M.E., Vignuzzi M., Connor J.H. (2017). Polyamines and their role in virus infection. Microbiol. Mol. Biol. Rev..

[153] Firpo M.R., Mastrodomenico V., Hawkins G.M., Prot M., Levillayer L., Gallagher T., Simon-Loriere E., Mounce B.C. (2021). Targeting polyamines inhibits coronavirus infection by reducing cellular attachment and entry. ACS Infect. Dis..

[154] Cruz-Pulido Y.E., Mounce B.C. (2023). Good cop, bad cop: Polyamines play both sides in host immunity and viral replication. Semin. Cell Dev. Biol..

[155] Lionetto L., Ulivieri M., Capi M., De Bernardini D., Fazio F., Petrucca A., Pomes L.M., De Luca O., Gentile G., Casolla B. (2021). Increased kynurenine-to-tryptophan ratio in the serum of patients infected with SARS-CoV2: An observational cohort study. Biochim. Biophys. Acta Mol. Basis. Dis..

[156] Thomas T., Stefanoni D., Reisz J.A., Nemkov T., Bertolone L., Francis R.O., Hudson K.E., Zimring J.C., Hansen K.C., Hod E.A. (2020). COVID-19 infection alters kynurenine and fatty acid metabolism, correlating with IL-6 levels and renal status. JCI Insight.

[157] Dehhaghi M., Heydari M., Panahi H.K.S., Lewin S.R., Heng B., Brew B.J., Guillemin G.J. (2024). The roles of the kynurenine pathway in COVID-19 neuropathogenesis. Infection.

[158] Karimi Z., Chenari M., Rezaie F., Karimi S., Parhizgari N., Mokhtari-Azad T. (2022). Proposed pathway linking respiratory infections with depression. Clin. Psychopharmacol. Neurosci..

[159] Stone T.W., Williams R.O. (2023). Modulation of T cells by tryptophan metabolites in the kynurenine pathway. Trends Pharmacol. Sci..

[160] Shih A.Y., Erb H., Sun X., Toda S., Kalivas P.W., Murphy T.H. (2006). Cystine/glutamate exchange modulates glutathione supply for neuroprotection from oxidative stress and cell proliferation. J. Neurosci..

[161] Qu Y., Haas de Mello A., Morris D.R., Jones-Hall Y.L., Ivanciuc T., Sattler R.A., Paessler S., Menachery V.D., Garofalo R.P., Casola A. (2023). SARS-CoV-2 inhibits NRF2-mediated antioxidant responses in airway epithelial cells and in the lung of a murine model of infection. Microbiol. Spectr..

[162] Fernandes I.G., de Brito C.A., Dos Reis V.M.S., Sato M.N., Pereira N.Z. (2020). SARS-CoV-2 and other respiratory viruses: What does oxidative stress have to do with it?. Oxid. Med. Cell. Longev..

[163] Yang X., Liu X., Nie Y., Zhan F., Zhu B. (2023). Oxidative stress and ROS-mediated cellular events in RSV infection: Potential protective roles of antioxidants. Virol. J..

[164] Jones J.T., Qian X., van der Velden J.L., Chia S.B., McMillan D.H., Flemer S., Hoffman S.M., Lahue K.G., Schneider R.W., Nolin J.D. (2016). Glutathione S-transferase pi modulates NF-κB activation and pro-inflammatory responses in lung epithelial cells. Redox Biol..

[165] Mazzarino R.C. (2021). Targeting future pandemics, a case for de novo purine synthesis and basic research. Front. Immunol..

[166] Qin C., Rao Y., Yuan H., Wang T.Y., Zhao J., Espinosa B., Liu Y., Zhang S., Savas A.C., Liu Q. (2022). SARS-CoV-2 couples evasion of inflammatory response to activated nucleotide synthesis. Proc. Natl. Acad. Sci. USA.

[167] Diehl F.F., Miettinen T.P., Elbashir R., Nabel C.S., Darnell A.M., Do B.T., Manalis S.R., Lewis C.A., Vander Heiden M.G. (2022). Nucleotide imbalance decouples cell growth from cell proliferation. Nat. Cell Biol..

[168] Sahan A.Z., Hazra T.K., Das S. (2018). The pivotal role of DNA repair in infection mediated-inflammation and cancer. Front. Microbiol..

[169] Okesli A., Khosla C., Bassik M.C. (2017). Human pyrimidine nucleotide biosynthesis as a target for antiviral chemotherapy. Curr. Opin. Biotechnol..

[170] Luganini A., Boschi D., Lolli M.L., Gribaudo G. (2025). DHODH inhibitors: What will it take to get them into the clinic as antivirals?. Antivir. Res..

[171] Ferrari D., Rubini M., Burns J.S. (2022). The potential of purinergic signaling to thwart viruses including SARS-CoV-2. Front. Immunol..

[172] Pacheco-Hernández L.M., Ramírez-Noyola J.A., Gómez-García I.A., Ignacio-Cortés S., Zúñiga J., Choreño-Parra J.A. (2022). Comparing the cytokine storms of COVID-19 and pandemic influenza. J. Interferon Cytokine Res..

[173] Ali E.S., Ben-Sahra I. (2023). Regulation of nucleotide metabolism in cancers and immune disorders. Trends Cell Biol..

[174] Khomich O.A., Kochetkov S.N., Bartosch B., Ivanov A.V. (2018). Redox biology of respiratory viral infections. Viruses.

[175] Forrester S.J., Kikuchi D.S., Hernandes M.S., Xu Q., Griendling K.K. (2018). Reactive oxygen species in metabolic and inflammatory signaling. Circ. Res..

[176] Amatore D., Sgarbanti R., Aquilano K., Baldelli S., Limongi D., Civitelli L., Nencioni L., Garaci E., Ciriolo M.R., Palamara A.T. (2015). Influenza virus replication in lung epithelial cells depends on redox-sensitive pathways activated by NOX4-derived ROS. Cell. Microbiol..

[177] Yang W.S., Yi Y.S., Kim D., Kim M.H., Park J.G., Kim E., Lee S.Y., Yoon K., Kim J.H., Park J. (2017). Nuclear factor kappa-B- and activator protein-1-mediated immunostimulatory activity of compound K in monocytes and macrophages. J. Ginseng Res..

[178] Hong Y., Boiti A., Vallone D., Foulkes N.S. (2024). Reactive oxygen species signaling and oxidative stress: Transcriptional regulation and evolution. Antioxidants.

[179] Bhol N.K., Bhanjadeo M.M., Singh A.K., Dash U.C., Ojha R.R., Majhi S., Duttaroy A.K., Jena A.B. (2024). The interplay between cytokines, inflammation, and antioxidants: Mechanistic insights and therapeutic potentials of various antioxidants and anti-cytokine compounds. Biomed. Pharmacother..

[180] Naiditch H., Betts M.R., Larman H.B., Levi M., Rosenberg A.Z. (2025). Immunologic and inflammatory consequences of SARS-CoV-2 infection and its implications in renal disease. Front. Immunol..

[181] Blevins H.M., Xu Y., Biby S., Zhang S. (2022). The NLRP3 inflammasome pathway: A review of mechanisms and inhibitors for the treatment of inflammatory diseases. Front. Aging Neurosci..

[182] Ziehr B.K., MacDonald J.A. (2024). Regulation of NLRPs by reactive oxygen species: A story of crosstalk. Biochim. Biophys. Acta Mol. Cell Res..

[183] Kolattukudy P.E., Niu J. (2012). Inflammation, endoplasmic reticulum stress, autophagy, and the monocyte chemoattractant protein-1/CCR2 pathway. Circ. Res..

[184] Pandey E., Nour A.S., Harris E.N. (2020). Prominent receptors of liver sinusoidal endothelial cells in liver homeostasis and disease. Front. Physiol..

[185] Andersson U., Ottestad W., Tracey K.J. (2020). Extracellular HMGB1: A therapeutic target in severe pulmonary inflammation including COVID-19?. Mol. Med..

[186] Shamilov R., Ackley T.W., Aneskievich B.J. (2020). Enhanced wound healing -and inflammasome- associated gene expression in TNFAIP3-interacting protein 1-(TNIP1-) deficient HaCaT keratinocytes parallels reduced reepithelialization. Mediat. Inflamm..

[187] Relja B., Land W.G. (2020). Damage-associated molecular patterns in trauma. Eur. J. Trauma Emerg. Surg..

[188] Afrose S.S., Junaid M., Akter Y., Tania M., Zheng M., Khan M.A. (2020). Targeting kinases with thymoquinone: A molecular approach to cancer therapeutics. Drug Discov. Today.

[189] Dantonio P.M., Klein M.O., Freire M.R.V.B., Araujo C.N., Chiacetti A.C., Correa R.G. (2018). Exploring major signaling cascades in melanomagenesis: A rationale route for targetted skin cancer therapy. Biosci. Rep..

[190] Slaine P.D., Kleer M., Duguay B.A., Pringle E.S., Kadijk E., Ying S., Balgi A., Roberge M., McCormick C., Khaperskyy D.A. (2021). Thiopurines activate an antiviral unfolded protein response that blocks influenza A virus glycoprotein accumulation. J. Virol..

[191] Féral K., Jaud M., Philippe C., Di Bella D., Pyronnet S., Rouault-Pierre K., Mazzolini L., Touriol C. (2021). ER stress and unfolded protein response in leukemia: Friend, Foe, or Both?. Biomolecules.

[192] Robinson C.M., Talty A., Logue S.E., Mnich K., Gorman A.M., Samali A. (2021). An emerging role for the unfolded protein response in pancreatic cancer. Cancers.

[193] Rashid H.O., Yadav R.K., Kim H.R., Chae H.J. (2015). ER stress: Autophagy induction, inhibition and selection. Autophagy.

[194] Dymkowska D. (2021). The involvement of autophagy in the maintenance of endothelial homeostasis: The role of mitochondria. Mitochondrion.

[195] Polonikov A. (2020). Endogenous deficiency of glutathione as the most likely cause of serious manifestations and death in COVID-19 patients. ACS Infect. Dis..

[196] Silvagno F., Vernone A., Pescarmona G.P. (2020). The role of glutathione in protecting against the severe inflammatory response triggered by COVID-19. Antioxidants.

[197] De Angelis M., Amatore D., Checconi P., Zevini A., Fraternale A., Magnani M., Hiscott J., De Chiara G., Palamara A.T., Nencioni L. (2022). Influenza virus down-modulates G6PD expression and activity to induce oxidative stress and promote its replication. Front. Cell. Infect. Microbiol..

[198] Hosakote Y.M., Liu T., Castro S.M., Garofalo R.P., Casola A. (2009). Respiratory syncytial virus induces oxidative stress by modulating antioxidant enzymes. Am. J. Respir. Cell Mol. Biol..

[199] Fukai T., Ushio-Fukai M. (2011). Superoxide dismutases: Role in redox signaling, vascular function, and diseases. Antioxid. Redox Signal..

[200] Chu J., Hua L., Liu X., Xiong H., Jiang F., Zhou W., Wang L., Xue G. (2024). Superoxide dismutase alterations in COVID-19: Implications for disease severity and mortality prediction in the context of omicron variant infection. Front. Immunol..

[201] Tavassolifar M.J., Aghdaei H.A., Sadatpour O., Maleknia S., Fayazzadeh S., Mohebbi S.R., Montazer F., Rabbani A., Zali M.R., Izad M. (2023). New insights into extracellular and intracellular redox status in COVID-19 patients. Redox Biol..

[202] Choi A.M., Knobil K., Otterbein S.L., Eastman D.A., Jacoby D.B. (1996). Oxidant stress responses in influenza virus pneumonia: Gene expression and transcription factor activation. Am. J. Physiol..

[203] Chen F., Chen L., Liang J., Chen Z., Zhang C., Zhang Z., Yang J. (2023). Potential role of superoxide dismutase 3 (SOD3) in resistance to Influenza A virus infection. Antioxidants.

[204] Hasan Anber Z.N., Oied Saleh B., Hassan Majed R. (2024). Assessment of oxidative stress parameters in Iraqi male patients with Covid-19; A case control study. Rep. Biochem. Mol. Biol..

[205] Rodríguez-Tomàs E., Iftimie S., Castañé H., Baiges-Gaya G., Hernández-Aguilera A., González-Viñas M., Castro A., Camps J., Joven J. (2021). Clinical performance of paraoxonase-1-related variables and novel markers of inflammation in coronavirus disease-19. A machine learning approach. Antioxidants.

[206] Gabaldó X., Juanpere M., Castañé H., Rodríguez-Tomàs E., López-Azcona A.F., Baiges-Gaya G., Castro L., Valverde-Díaz E., Muñoz-Blázquez A., Giménez-Cuenca L. (2022). Usefulness of the measurement of serum paraoxonase-1 arylesterase activity in the diagnoses of COVID-19. Biomolecules.

[207] Cho K.H., Kim J.R., Lee I.C., Kwon H.J. (2021). Native high-density lipoproteins (HDL) with higher paraoxonase exerts a potent antiviral effect against SARS-CoV-2 (COVID-19), while glycated HDL lost the antiviral activity. Antioxidants.

[208] Wang B., Zhang L., Dai T., Qin Z., Lu H., Zhang L., Zhou F. (2021). Liquid-liquid phase separation in human health and diseases. Signal Transduct. Target. Ther..

[209] Huai Y., Mao W., Wang X., Lin X., Li Y., Chen Z., Qian A. (2022). How do RNA binding proteins trigger liquid-liquid phase separation in human health and diseases?. Biosci. Trends.

[210] Chau B.A., Chen V., Cochrane A.W., Parent L.J., Mouland A.J. (2023). Liquid-liquid phase separation of nucleocapsid proteins during SARS-CoV-2 and HIV-1 replication. Cell Rep..

[211] Lu S., Ye Q., Singh D., Cao Y., Diedrich J.K., Yates J.R., Villa E., Cleveland D.W., Corbett K.D. (2021). The SARS-CoV-2 nucleocapsid phosphoprotein forms mutually exclusive condensates with RNA and the membrane-associated M protein. Nat. Commun..

[212] Savastano A., Ibáñez de Opakua A., Rankovic M., Zweckstetter M. (2020). Nucleocapsid protein of SARS-CoV-2 phase separates into RNA-rich polymerase-containing condensates. Nat. Commun..

[213] Zachrdla M., Savastano A., Ibáñez de Opakua A., Cima-Omori M.S., Zweckstetter M. (2022). Contributions of the N-terminal intrinsically disordered region of the severe acute respiratory syndrome coronavirus 2 nucleocapsid protein to RNA-induced phase separation. Protein Sci..

[214] Nichols S.L., Nilsson E.M., Brown-Harding H., LaConte L.E.W., Acker J., Borodavka A., McDonald Esstman S. (2023). Flexibility of the rotavirus NSP2 C-terminal region supports factory formation via liquid-liquid phase separation. J. Virol..

[215] Yang W., Wang Y., Liu G., Wang Y., Wu C. (2024). TPM4 condensates glycolytic enzymes and facilitates actin reorganization under hyperosmotic stress. Cell Discov..

[216] Chen X., Jiang B., Gu Y., Yue Z., Liu Y., Lei Z., Yang G., Deng M., Zhang X., Luo Z. (2024). SARS-CoV-2 nucleocapsid protein interaction with YBX1 displays oncolytic properties through PKM mRNA destabilization. Mol. Cancer.

[217] Alenquer M., Vale-Costa S., Etibor T.A., Ferreira F., Sousa A.L., Amorim M.J. (2019). Influenza A virus ribonucleoproteins form liquid organelles at endoplasmic reticulum exit sites. Nat. Commun..

[218] Khaperskyy D.A., Emara M.M., Johnston B.P., Anderson P., Hatchette T.F., McCormick C. (2014). Influenza A virus host shutoff disables antiviral stress-induced translation arrest. PLoS Pathog..

[219] Girdhar A., Guo L. (2022). Regulating phase transition in neurodegenerative diseases by nuclear import rReceptors. Biology.

[220] Risso-Ballester J., Rameix-Welti M.A. (2023). Spatial resolution of virus replication: RSV and cytoplasmic inclusion bodies. Adv. Virus Res..

[221] Van Royen T., Rossey I., Sedeyn K., Schepens B., Saelens X. (2022). How RSV proteins join forces to overcome the host innate immune response. Viruses.

[222] Igelmann S., Lessard F., Ferbeyre G. (2022). Liquid-liquid phase separation in cancer signaling, metabolism and anticancer therapy. Cancers.

[223] He P., Zhang B., Jiang W., Zhu F., Liang Z., Gao L., Zhang Y., Wang Y., Wu C., Tang C. (2025). PKM2 is a key factor to regulate neurogenesis and cognition by controlling lactate homeostasis. Stem Cell Rep..

[224] Liu Z., Le Y., Chen H., Zhu J., Lu D. (2021). Role of PKM2-mediated immunometabolic reprogramming on development of cytokine storm. Front. Immunol..

[225] Alberti S., Gladfelter A., Mittag T. (2019). Considerations and challenges in studying liquid-liquid phase separation and biomolecular condensates. Cell.

[226] O’Flynn B.G., Mittag T. (2021). The role of liquid-liquid phase separation in regulating enzyme activity. Curr. Opin. Cell Biol..

[227] Tang Y., Zhang Y., Yang N., Shi H., Fu Y., Bai B., Li B., Yang B., Liu G. (2025). TGEV NSP1 enhances viral replication through antagonizing stress granule formation. Vet. Microbiol..

[228] Yang Y., Willis T.L., Button R.W., Strang C.J., Fu Y., Wen X., Grayson P.R.C., Evans T., Sipthorpe R.J., Roberts S.L. (2019). Cytoplasmic DAXX drives SQSTM1/p62 phase condensation to activate Nrf2-mediated stress response. Nat. Commun..

[229] Saito Y., Kimura W. (2021). Roles of phase separation for cellular redox maintenance. Front. Genet..

[230] Mayneris-Perxachs J., Moreno-Navarrete J.M., Ballanti M., Monteleone G., Alessandro Paoluzi O., Mingrone G., Lefebvre P., Staels B., Federici M., Puig J. (2021). Lipidomics and metabolomics signatures of SARS-CoV-2 mediators/receptors in peripheral leukocytes, jejunum and colon. Comput. Struct. Biotechnol. J..

[231] Xu S.W., Ilyas I., Weng J.P. (2023). Endothelial dysfunction in COVID-19: An overview of evidence, biomarkers, mechanisms and potential therapies. Acta Pharmacol. Sin..

[232] Li H., Ernst C., Kolonko-Adamska M., Greb-Markiewicz B., Man J., Parissi V., Ng B.W. (2022). Phase separation in viral infections. Trends Microbiol..

[233] Wang L., Zhou W. (2024). Phase separation as a new form of regulation in innate immunity. Mol. Cell.

[234] Thaker S.K., Ch’ng J., Christofk H.R. (2019). Viral hijacking of cellular metabolism. BMC Biol..

[235] Yan Y., Chen J., Liang Q., Zheng H., Ye Y., Nan W., Zhang X., Gao H., Li Y. (2022). Metabolomics profile in acute respiratory distress syndrome by nuclear magnetic resonance spectroscopy in patients with community-acquired pneumonia. Respir. Res..

[236] Hasin Y., Seldin M., Lusis A. (2017). Multi-omics approaches to disease. Genome Biol..

[237] Camps J., Jiménez-Franco A., García-Pablo R., Joven J., Arenas M. (2025). Artificial intelligence-driven integration of multi-omics and radiomics: A new hope for precision cancer diagnosis and prognosis. Biochim. Biophys. Acta Mol. Basis Dis..

[238] Hu L., Liu J., Zhang W., Wang T., Zhang N., Lee Y.H., Lu H. (2020). Functional metabolomics decipher biochemical functions and associated mechanisms underlie small-molecule metabolism. Mass Spectrom. Rev..

[239] Fuertes-Martín R., Taverner D., Vallvé J., Paredes S., Masana L., Correig Blanchar X. (2018). Characterization of 1H NMR plasma glycoproteins as a new strategy to identify inflammatory patterns in rheumatoid arthritis. J. Proteome Res..

[240] Fuertes-Martin R., Moncayo S., Insenser M., Martínez-García M.A., Luque-Ramírez M., Grau N.A., Blanchar X.C., Escobar-Morreale H.F. (2019). Glycoprotein A and B height-to-width ratios as obesity-independent novel biomarkers of low-grade chronic inflammation in women with polycystic ovary syndrome (PCOS). J. Proteome Res..

[241] Ghini V., Meoni G., Vignoli A., Di Cesare F., Tenori L., Turano P., Luchinat C. (2023). Fingerprinting and profiling in metabolomics of biosamples. Prog. Nucl. Magn. Reson. Spectrosc..

[242] Riera-Borrull M., Rodríguez-Gallego E., Hernández-Aguilera A., Luciano F., Ras R., Cuyàs E., Camps J., Segura-Carretero A., Menendez J.A., Joven J. (2016). Exploring the process of energy generation in pathophysiology by targeted metabolomics: Performance of a simple and quantitative method. J. Am. Soc. Mass Spectrom..

[243] Cuyàs E., Fernández-Arroyo S., Verdura S., García R.Á., Stursa J., Werner L., Blanco-González E., Montes-Bayón M., Joven J., Viollet B. (2018). Metformin regulates global DNA methylation via mitochondrial one-carbon metabolism. Oncogene.

[244] Nikolskiy I., Siuzdak G., Patti G.J. (2015). Discriminating precursors of common fragments for large-scale metabolite profiling by triple quadrupole mass spectrometry. Bioinformatics.

[245] Patti G.J. (2011). Separation strategies for untargeted metabolomics. J. Sep. Sci..

[246] Ivanisevic J., Want E.J. (2019). From samples to insights into metabolism: Uncovering biologically relevant information in LC-HRMS metabolomics data. Meta.

[247] Strimbu K., Tavel J.A. (2010). What are biomarkers?. Curr. Opin. HIV AIDS.

[248] FDA-NIH Biomarker Working Group (2016). BEST (Biomarkers, EndpointS, and other Tools) Resource [Internet].

[249] Pepe M.S., Janes H., Longton G., Leisenring W., Newcomb P. (2004). Limitations of the odds ratio in gauging the performance of a diagnostic, prognostic, or screening marker. Am. J. Epidemiol..

[250] Castañé H., Iftimie S., Baiges-Gaya G., Rodríguez-Tomàs E., Jiménez-Franco A., López-Azcona A.F., Garrido P., Castro A., Camps J., Joven J. (2022). Machine learning and semi-targeted lipidomics identify distinct serum lipid signatures in hospitalized COVID-19-positive and COVID-19-negative patients. Metabolism.

[251] Mai M., Tönjes A., Kovacs P., Stumvoll M., Fiedler G.M., Leichtle A.B. (2013). Serum levels of acylcarnitines are altered in prediabetic conditions. PLoS ONE.

[252] Ayres J.S. (2020). A metabolic handbook for the COVID-19 pandemic. Nat. Metab..

[253] Otsubo C., Bharathi S., Uppala R., Ilkayeva O.R., Wang D., McHugh K., Zou Y., Wang J., Alcorn J.F., Zuo Y.Y. (2015). Long-chain acylcarnitines reduce lung function by inhibiting pulmonary surfactant. J. Biol. Chem..

[254] Wu D., Shu T., Yang X., Song J.X., Zhang M., Yao C., Liu W., Huang M., Yu Y., Yang Q. (2020). Plasma metabolomic and lipidomic alterations associated with COVID-19. Natl. Sci. Rev..

[255] Song J.W., Lam S.M., Fan X., Cao W.J., Wang S.Y., Tian H., Chua G.H., Zhang C., Meng F.P., Xu Z. (2020). Omics-driven systems interrogation of metabolic dysregulation in COVID-19 pathogenesis. Cell Metab..

[256] Barberis E., Timo S., Amede E., Vanella V.V., Puricelli C., Cappellano G., Raineri D., Cittone M.G., Rizzi E., Pedrinelli A.R. (2020). Large-scale plasma analysis revealed new mechanisms and molecules associated with the host response to SARS-CoV-2. Int. J. Mol. Sci..

[257] Fraser D.D., Slessarev M., Martin C.M., Daley M., Patel M.A., Miller M.R., Patterson E.K., O’Gorman D.B., Gill S.E., Wishart D.S. (2020). Metabolomics profiling of critically ill coronavirus disease 2019 patients: Identification of diagnostic and prognostic biomarkers. Crit. Care Explor..

[258] Delafiori J., Navarro L.C., Siciliano R.F., de Melo G.C., Busanello E.N.B., Nicolau J.C., Sales G.M., de Oliveira A.N., Val F.F.A., de Oliveira D.N. (2021). Covid-19 automated diagnosis and risk assessment through metabolomics and machine learning. Anal. Chem..

[259] Hao Y., Zhang Z., Feng G., Chen M., Wan Q., Lin J., Wu L., Nie W., Chen S. (2021). Distinct lipid metabolic dysregulation in asymptomatic COVID-19. iScience.

[260] Lam S.M., Zhang C., Wang Z., Ni Z., Zhang S., Yang S., Huang X., Mo L., Li J., Lee B. (2021). A multi-omics investigation of the composition and function of extracellular vesicles along the temporal trajectory of COVID-19. Nat. Metab..

[261] Kyle J.E., Burnum-Johnson K.E., Wendler J.P., Eisfeld A.J., Halfmann P.J., Watanabe T., Sahr F., Smith R.D., Kawaoka Y., Waters K.M. (2019). Plasma lipidome reveals critical illness and recovery from human Ebola virus disease. Proc. Natl. Acad. Sci. USA.

[262] Nguyen M., Bourredjem A., Piroth L., Bouhemad B., Jalil A., Pallot G., Le Guern N., Thomas C., Pilot T., Bergas V. (2021). High plasma concentration of non-esterified polyunsaturated fatty acids is a specific feature of severe COVID-19 pneumonia. Sci. Rep..

[263] Bizkarguenaga M., Bruzzone C., Gil-Redondo R., SanJuan I., Martin-Ruiz I., Barriales D., Palacios A., Pasco S.T., González-Valle B., Laín A. (2022). Uneven metabolic and lipidomic profiles in recovered COVID-19 patients as investigated by plasma NMR metabolomics. NMR Biomed..

[264] Bruzzone C., Bizkarguenaga M., Gil-Redondo R., Diercks T., Arana E., García de Vicuña A., Seco M., Bosch A., Palazón A., San Juan I. (2020). SARS-CoV-2 infection dysregulates the metabolomic and lipidomic profiles of serum. iScience.

[265] Iftimie S., Gabaldó-Barrios X., Penadés-Nadal J., Canela-Capdevila M., Piñana R., Jiménez-Franco A., López-Azcona A.F., Castañé H., Cárcel M., Camps J. (2024). Serum levels of arachidonic acid, interleukin-6, and C-reactive protein as potential indicators of pulmonary viral infections: Comparative analysis of influenza A, respiratory syncytial virus infection, and COVID-19. Viruses.

[266] Yan B., Chu H., Yang D., Sze K.H., Lai P.M., Yuan S., Shuai H., Wang Y., Kao R.Y., Chan J.F. (2019). Characterization of the lipidomic profile of human coronavirus-infected cells: Implications for lipid metabolism remodeling upon coronavirus replication. Viruses.

[267] Shen B., Yi X., Sun Y., Bi X., Du J., Zhang C., Quan S., Zhang F., Sun R., Qian L. (2020). Proteomic and metabolomic characterization of COVID-19 patient sera. Cell.

[268] Torrente-Rodríguez R.M., Ruiz-Valdepeñas Montiel V., Iftimie S., Montero-Calle A., Pingarrón J.M., Castro A., Camps J., Barderas R., Campuzano S., Joven J. (2024). Contributing to the management of viral infections through simple immunosensing of the arachidonic acid serum level. Mikrochim. Acta.

[269] Meoni G., Ghini V., Maggi L., Vignoli A., Mazzoni A., Salvati L., Capone M., Vanni A., Tenori L., Fontanari P. (2021). Metabolomic/lipidomic profiling of COVID-19 and individual response to tocilizumab. PLoS Pathog..

[270] Julkunen H., Cichońska A., Slagboom P.E., Würtz P., Nightingale Health UK Biobank Initiative (2021). Metabolic biomarker profiling for identification of susceptibility to severe pneumonia and COVID-19 in the general population. Elife.

[271] Amigó N., Martínez-Micaelo N., Velasco M., Casas M.L., Correig X., Guijarro C. (2024). Identification of a molecular signature associated with covid-19 severity using a comprehensive 1H-NMR serum metabolomics profiling strategy. Atherosclerosis.

[272] López-Hernández Y., Oropeza-Valdez J.J., García Lopez D.A., Borrego J.C., Murgu M., Valdez J., López J.A., Monárrez-Espino J. (2023). Untargeted analysis in post-COVID-19 patients reveals dysregulated lipid pathways two years after recovery. Front. Mol. Biosci..

[273] Washirasaksiri C., Sayabovorn N., Ariyakunaphan P., Kositamongkol C., Chaisathaphol T., Sitasuwan T., Tinmanee R., Auesomwang C., Nimitpunya P., Woradetsittichai D. (2023). Long-term multiple metabolic abnormalities among healthy and high-risk people following nonsevere COVID-19. Sci. Rep..

[274] Kyo M., Zhu Z., Shibata R., Fujiogi M., Mansbach J.M., Camargo C.A., Hasegawa K. (2023). Respiratory virus-specific nasopharyngeal lipidome signatures and severity in infants with bronchiolitis: A prospective multicenter study. J. Infect. Dis..

[275] Loo R.L., Lodge S., Kimhofer T., Bong S.H., Begum S., Whiley L., Gray N., Lindon J.C., Nitschke P., Lawler N.G. (2020). Quantitative in-vitro diagnostic NMR spectroscopy for lipoprotein and metabolite measurements in plasma and serum: Recommendations for analytical artifact minimization with special reference to COVID-19/SARS-CoV-2 samples. J. Proteome Res..

[276] Corn G., Lund M., Andersson N.W., Dohlmann T.L., Hlatky M.A., Wohlfahrt J., Melbye M. (2024). Low-density lipoprotein cholesterol response to statins according to comorbidities and co-medications: A population-based study. Am. Heart J..

[277] Zimodro J.M., Mucha M., Berthold H.K., Gouni-Berthold I. (2024). Lipoprotein metabolism, dyslipidemia, and lipid-lowering therapy in women: A comprehensive review. Pharmaceuticals.

[278] Berta E., Zsíros N., Bodor M., Balogh I., Lőrincz H., Paragh G., Harangi M. (2022). Clinical aspects of genetic and non-genetic cardiovascular risk factors in familial hypercholesterolemia. Genes.

[279] Baiges-Gaya G., Iftimie S., Castañé H., Rodríguez-Tomàs E., Jiménez-Franco A., López-Azcona A.F., Castro A., Camps J., Joven J. (2023). Combining semi-targeted metabolomics and machine learning to identify metabolic alterations in the serum and urine of hospitalized patients with COVID-19. Biomolecules.

[280] Sun H., Zhang A.H., Song Q., Fang H., Liu X.Y., Su J., Yang L., Yu M.D., Wang X.J. (2018). Functional metabolomics discover pentose and glucuronate interconversion pathways as promising targets for Yang Huang syndrome treatment with Yinchenhao Tang. RSC Adv..

[281] Chen S., Niu C., Lv W. (2022). Multi-omics insights reveal the remodeling of gut mycobiome with *P. gingivalis*. Front. Cell. Infect. Microbiol..

[282] Lu X., Liu T., Zhou J., Liu J., Yuan Z., Guo L. (2022). Subgingival microbiome in periodontitis and type 2 diabetes mellitus: An exploratory study using metagenomic sequencing. J. Periodontal Implant Sci..

[283] Xiong H., Li N., Zhao L., Li Z., Yu Y., Cui X., Liu Q., Zhao C. (2022). Integrated serum pharmacochemistry, metabolomics, and network pharmacology to reveal the material basis and mechanism of Danggui Shaoyao San in the treatment of primary dysmenorrhea. Front. Pharmacol..

[284] Wu Y., Li K., Zeng M., Qiao B., Zhou B. (2022). Serum metabolomics analysis of the anti-inflammatory effects of gallic acid on rats with acute inflammation. Front. Pharmacol..

[285] Li Y., Zhang D., Gao X., Wang X., Zhang L. (2022). 2′- and 3′-ribose modifications of nucleotide analogues establish the structural basis to inhibit the viral replication of SARS-CoV-2. J. Phys. Chem. Lett..

[286] Guo X., Wu S., Li N., Lin Q., Liu L., Liang H., Niu Y., Huang Z., Fu X. (2019). Accelerated metabolite levels of aerobic glycolysis and the pentose phosphate pathway are required for efficient replication of infectious spleen and kidney necrosis virus in Chinese perch brain cells. Biomolecules.

[287] Sen S., Kaminiski R., Deshmane S., Langford D., Khalili K., Amini S., Datta P.K. (2015). Role of hexokinase-1 in the survival of HIV-1-infected macrophages. Cell Cycle.

[288] Stincone A., Prigione A., Cramer T., Wamelink M.M., Campbell K., Cheung E., Olin-Sandoval V., Grüning N.M., Krüger A., Tauqeer Alam M. (2015). The return of metabolism: Biochemistry and physiology of the pentose phosphate pathway. Biol. Rev. Camb. Philos. Soc..

[289] Chen I.T., Aoki T., Huang Y.T., Hirono I., Chen T.C., Huang J.Y. (2011). White spot Syndrome virus induces metabolic changes resembling the Warburg effect in shrimp hemocytes in the early stage of infection. J. Virol..

[290] Pérez-Torres I., Soto M.E., Guarner-Lans V., Manzano-Pech L., Soria-Castro E. (2022). The possible role of glucose-6-phosphate dehydrogenase in the SARS-CoV-2 infection. Cells.

[291] Bojkova D., Costa R., Reus P., Bechtel M., Jaboreck M.C., Olmer R., Martin U., Ciesek S., Michaelis M., Cinatl J. (2021). Targeting the pentose phosphate pathway for SARS-CoV-2 therapy. Metabolites.

[292] Isaacs C.E., Kim K.S., Thormar H. (1994). Inactivation of enveloped viruses in human bodily fluids by purified lipids. Ann. N. Y. Acad. Sci..

[293] Nefedova E., Koptev V., Bobikova A.S., Cherepushkina V., Mironova T., Afonyushkin V., Shkil N., Donchenko N., Kozlova Y., Sigareva N. (2021). The infectious bronchitis coronavirus pneumonia model presenting a novel insight for the SARS-CoV-2 dissemination route. Vet. Sci..

[294] Thormar H., Isaacs C.E., Brown H.R., Barshatzky M.R., Pessolano T. (1987). Inactivation of enveloped viruses and killing of cells by fatty acids and monoglycerides. Antimicrob. Agents Chemother..

[295] Cheudjeu A. (2020). Correlation of D-xylose with severity and morbidity-related factors of COVID-19 and possible therapeutic use of D-xylose and antibiotics for COVID-19. Life Sci..

[296] Ferreira A.S., Ad Souza M., Raposo N.R.B., Ferreira A.P., Silva S.S. (2011). Xylitol inhibits J774A.1 macrophage adhesion in vitro. Braz. Arch. Biol. Technol..

[297] Xu M.L., Wi G., Kim H.J., Kim H.J. (2016). Ameliorating effect of dietary xylitol on human respiratory syncytial virus (hRSV) infection. Biol. Pharm. Bull..

[298] Yin S.Y., Kim H.J., Kim H.J. (2014). Protective effect of dietary xylitol on influenza A virus infection. PLoS ONE.

[299] Anand S., Mande S.S. (2018). Diet, microbiota and gut-lung connection. Front. Microbiol..

[300] Shukla S.D., Budden K.F., Neal R., Hansbro P.M. (2017). Microbiome effects on immunity, health and disease in the lung. Clin. Transl. Immunol..

[301] Russell S.L., Gold M.J., Willing B.P., Thorson L., Mcnagny K.M., Finlay B.B. (2013). Perinatal antibiotic treatment affects murine microbiota, immune responses and allergic asthma. Gut Microbes.

[302] Looft T., Allen H.K. (2012). Collateral effects of antibiotics on mammalian gut microbiomes. Gut Microbes.

[303] Xie J., Cho H., Lin B.M., Pillai M., Heimisdottir L.H., Bandyopadhyay D., Zou F., Roach J., Divaris K., Wu D. (2021). Improved metabolite prediction using microbiome data-based elastic net models. Front. Cell. Infect. Microbiol..

[304] Wan J., Zhang Y., He W., Tian Z., Lin J., Liu Z., Li Y., Chen M., Han S., Liang J. (2022). Gut microbiota and metabolite changes in patients with ulcerative colitis and Clostridioides difficile infection. Front. Microbiol..

[305] Colonetti K., de Carvalho E.L., Rangel D.L., Pinto P.M., Roesch L.F.W., Pinheiro F.C., Schwartz I.V.D. (2022). Are the bacteria and their metabolites contributing for gut inflammation on GSD-Ia patients?. Metabolites.

[306] Liu A., Ma T., Xu N., Jin H., Zhao F., Kwok L.Y., Zhang H., Zhang S., Sun Z. (2021). Adjunctive probiotics alleviates asthmatic symptoms via modulating the gut microbiome and serum metabolome. Microbiol. Spectr..

[307] Tong W., Hannou S.A., Wang Y., Astapova I., Sargsyan A., Monn R., Thiriveedi V., Li D., McCann J.R., Rawls J.F. (2022). The intestine is a major contributor to circulating succinate in mice. FASEB J..

[308] Nagata N., Takeuchi T., Masuoka H., Aoki R., Ishikane M., Iwamoto N., Sugiyama M., Suda W., Nakanishi Y., Terada-Hirashima J. (2023). Human gut microbiota and its metabolites impact immune responses in COVID-19 and its complications. Gastroenterology.

[309] Liao J., Li Q., Lei C., Yu W., Deng J., Guo J., Han Q., Hu L., Li Y., Pan J. (2021). Toxic effects of copper on the jejunum and colon of pigs: Mechanisms related to gut barrier dysfunction and inflammation influenced by the gut microbiota. Food Funct..

[310] Yu W., Shang J., Guo R., Zhang F., Zhang W., Zhang Y., Wu F., Ren H., Liu C., Xiao J. (2020). The gut microbiome in differential diagnosis of diabetic kidney disease and membranous nephropathy. Ren. Fail..

[311] Yin J., Li Y., Han H., Liu Z., Zeng X., Li T., Yin Y. (2018). Long-term effects of lysine concentration on growth performance, intestinal microbiome, and metabolic profiles in a pig model. Food Funct..

[312] Ren L., Zhang W., Zhang J., Zhang J., Zhang H., Zhu Y., Meng X., Yi Z., Wang R. (2021). Influenza A virus (H1N1) infection induces glycolysis to facilitate viral replication. Virol. Sin..

[313] Martín-Vicente M., González-Riaño C., Barbas C., Jiménez-Sousa M.Á., Brochado-Kith O., Resino S., Martínez I. (2020). Metabolic changes during respiratory syncytial virus infection of epithelial cells. PLoS ONE.

[314] Fratta Pasini A.M., Stranieri C., Girelli D., Busti F., Cominacini L. (2021). Is ferroptosis a key component of the process leading to multiorgan damage in COVID-19?. Antioxidants.

[315] Morris G., Bortolasci C.C., Puri B.K., Olive L., Marx W., O’Neil A., Athan E., Carvalho A.F., Maes M., Walder K. (2020). The pathophysiology of SARS-CoV-2: A suggested model and therapeutic approach. Life Sci..

[316] Cecchini R., Cecchini A.L. (2020). SARS-CoV-2 infection pathogenesis is related to oxidative stress as a response to aggression. Med. Hypotheses.

[317] Wang M., Cao R., Zhang L., Yang X., Liu J., Xu M., Shi Z., Hu Z., Zhong W., Xiao G. (2020). Remdesivir and chloroquine effectively inhibit the recently emerged novel coronavirus (2019-nCoV) in vitro. Cell Res..

[318] Watkins L.C., DeGrado W.F., Voth G.A. (2020). Influenza A M2 inhibitor binding understood through mechanisms of excess proton stabilization and channel dynamics. J. Am. Chem. Soc..

[319] Kumar G., Sakharam K.A. (2024). Tackling Influenza A virus by M2 ion channel blockers: Latest progress and limitations. Eur. J. Med. Chem..

[320] CDC Antiviral Drug Resistance Among Influenza Viruses. https://www.cdc.gov/flu/hcp/antivirals/antiviral-drug-resistance.html#:~:text=Amantadine%20and%20Rimantadine%20(Adamantanes)&text=Resistance%20to%20adamantanes%20remains%20high,circulating%20influenza%20A%20virus%20strains.

[321] Heida R., Bhide Y.C., Gasbarri M., Kocabiyik Ö., Stellacci F., Huckriede A.L.W., Hinrichs W.L.J., Frijlink H.W. (2021). Advances in the development of entry inhibitors for sialic-acid-targeting viruses. Drug. Discov. Today.

[322] De Clercq E. (2015). Chemotherapy of respiratory syncytial virus infections: The final breakthrough. Int. J. Antimicrob Agents..

[323] Song Q., Zhu H., Qiu M., Cai J., Hu Y., Yang H., Rao S., Li Y., Li M., Hu L. (2024). A new mechanism of respiratory syncytial virus entry inhibition by small-molecule to overcome K394R-associated resistance. mBio.

[324] Ammer E., Nietzsche S., Rien C., Kühnl A., Mader T., Heller R., Sauerbrei A., Henke A. (2015). The anti-obesity drug orlistat reveals anti-viral activity. Med. Microbiol. Immunol..

[325] Kow C.S., Hasan S.S. (2020). Meta-analysis of effect of statins in patients with COVID-19. Am. J. Cardiol..

[326] Diaz-Arocutipa C., Melgar-Talavera B., Alvarado-Yarasca Á., Saravia-Bartra M.M., Cazorla P., Belzusarri I., Hernandez A.V. (2021). Statins reduce mortality in patients with COVID-19: An updated meta-analysis of 147 824 patients. Int. J. Infect. Dis..

[327] Onorato D., Pucci M., Carpene G., Henry B.M., Sanchis-Gomar F., Lippi G. (2021). Protective effects of statins administration in European and North American patients infected with COVID-19: A meta-analysis. Semin. Thromb. Hemost..

[328] Florêncio de Mesquita C., Rivera A., Araújo B., Durães V.L., Queiroz I., Carvalho V.H., Haque T., Bes T.M. (2024). Adjunctive statin therapy in patients with Covid-19: A systematic review and meta-analysis of randomized controlled trials. Am. J. Med..

[329] Arnardottir H., Pawelzik S.C., Öhlund Wistbacka U., Artiach G., Hofmann R., Reinholdsson I., Braunschweig F., Tornvall P., Religa D., Bäck M. (2021). Stimulating the resolution of inflammation through omega-3 polyunsaturated fatty acids in COVID-19: Rationale for the *COVID-Omega-F* trial. Front. Physiol..

[330] Magulick J.P., Frei C.R., Ali S.K., Mortensen E.M., Pugh M.J., Oramasionwu C.U., Daniels K.R., Mansi I.A. (2014). The effect of statin therapy on the incidence of infections: A retrospective cohort analysis. Am. J. Med. Sci..

[331] Vahedian-Azimi A., Mannarino M.R., Shojaie S., Rahimibashar F., Galeh H.E.G., Banach M., Bianconi V., Pirro M., Sahebkar A. (2022). The effect of statins on the prevalence and mortality of influenza virus infection: A systematic review and meta-analysis. Arch. Med. Sci..

[332] Djuricic I., Calder P.C. (2024). Omega-3 (n-3) fatty acid-statin interaction: Evidence for a novel therapeutic strategy for atherosclerotic cardiovascular disease. Nutrients.

[333] Huang Z., Chavda V.P., Vora L.K., Gajjar N., Apostolopoulos V., Shah N., Chen Z.S. (2022). 2-deoxy-D-glucose and its derivatives for the COVID-19 treatment: An update. Front. Pharmacol..

[334] Bhatt A.N., Shenoy S., Munjal S., Chinnadurai V., Agarwal A., Vinoth Kumar A., Shanavas A., Kanwar R., Chandna S. (2022). 2-deoxy-D-glucose as an adjunct to standard of care in the medical management of COVID-19: A proof-of-concept and dose-ranging randomised phase II clinical trial. BMC Infect. Dis..

[335] Verma A., Adhikary A., Woloschak G., Dwarakanath B.S., Papineni R.V.L. (2020). A combinatorial approach of a polypharmacological adjuvant 2-deoxy-D-glucose with low dose radiation therapy to quell the cytokine storm in COVID-19 management. Int. J. Radiat. Biol..

[336] Sandepogu T.S., Dara C., Mallamgunta S., Jogi S., Sree Podila K., Chandrasekhar J., N V., Sivakumar S. (2024). Role of 2-deoxy-D-glucose in enhancing the efficacy of standard of care for moderate to severe COVID-19: A comparative analysis of clinical outcomes. Cureus.

[337] Ergashev A., Shi F., Liu Z., Pan Z., Xie H., Kong L., Wu L., Sun H., Jin Y., Kong H. (2024). KAN0438757, a novel PFKFB3 inhibitor, prevent the progression of severe acute pancreatitis via the Nrf2/HO-1 pathway in infiltrated macrophage. Free Radic. Biol. Med..

[338] Yuan S., Ye Z.W., Chu N. (2023). Host PFKFB3-dependent glycolytic reprogramming as a broad-spectrum antiviral strategy. Open Forum Infect. Dis..

[339] Klarer A.C., O’Neal J., Imbert-Fernandez Y., Clem A., Ellis S.R., Clark J., Clem B., Chesney J., Telang S. (2014). Inhibition of 6-phosphofructo-2-kinase (PFKFB3) induces autophagy as a survival mechanism. Cancer Metab..

[340] Clem B.F., O’Neal J., Tapolsky G., Clem A.L., Imbert-Fernandez Y., Kerr D.A., Klarer A.C., Redman R., Miller D.M., Trent J.O. (2013). Targeting 6-phosphofructo-2-kinase (PFKFB3) as a therapeutic strategy against cancer. Mol. Cancer Ther..

[341] Boyd S., Brookfield J.L., Critchlow S.E., Cumming I.A., Curtis N.J., Debreczeni J., Degorce S.L., Donald C., Evans N.J., Groombridge S. (2015). Structure-based design of potent and selective inhibitors of the metabolic kinase PFKFB3. J. Med. Chem..

[342] Xu X., Zhao Y., Zhu Z., Wen W., Li X. (2025). Mitofusin-mediated mitochondrial fusion inhibits Pseudorabies virus infection in porcine cells. Vet. Sci..

[343] Gregorczyk-Zboroch K., Szulc-Dąbrowska L., Pruchniak P., Gieryńska M., Mielcarska M.B., Biernacka Z., Wyżewski Z., Lasocka I., Świtlik W., Szepietowska A. (2024). Modifications of mitochondrial network morphology affect the MAVS-dependent immune response in L929 murine fibroblasts during Ectromelia virus infection. Pathogens.

[344] Zhang R., Wang X., Ni L., Di X., Ma B., Niu S., Liu C., Reiter R.J. (2020). COVID-19: Melatonin as a potential adjuvant treatment. Life Sci..

[345] Gutierrez-Mariscal F.M., Arenas-de Larriva A.P., Limia-Perez L., Romero-Cabrera J.L., Yubero-Serrano E.M., López-Miranda J. (2020). Coenzyme Q_10_ supplementation for the reduction of oxidative stress: Clinical implications in the treatment of chronic diseases. Int. J. Mol. Sci..

[346] Gutierrez-Mariscal F.M., Yubero-Serrano E.M., Villalba J.M., Lopez-Miranda J. (2019). Coenzyme Q_10_: From bench to clinic in aging diseases, a translational review. Crit. Rev. Food Sci. Nutr..

[347] Shikama Y., Otsuka K., Shikama Y., Furukawa M., Ishimaru N., Matsushita K. (2025). Involvement of metformin and aging in salivary expression of ACE2 and TMPRSS2. Biofactors.

[348] Chen X., Shi S., Sun H., Zhou L., Wang H., Li Y., Gilson E., Lu Y., Hu L., Ye J. (2025). Metformin alleviates inflammatory response and severity rate of COVID-19 infection in elderly individuals. Sci. Rep..

[349] Rocha M., Hernandez-Mijares A., Garcia-Malpartida K., Bañuls C., Bellod L., Victor V.M. (2010). Mitochondria-targeted antioxidant peptides. Curr. Pharm. Des..

[350] Gasanoff E.S., Yaguzhinsky L., Garab G. (2021). Cardiolipin, non-bilayer structures and mitochondrial bioenergetics: Relevance to cardiovascular disease. Cells.

[351] Ding X.W., Robinson M., Li R., Aldhowayan H., Geetha T., Babu J.R. (2021). Mitochondrial dysfunction and beneficial effects of mitochondria-targeted small peptide SS-31 in Diabetes Mellitus and Alzheimer’s disease. Pharmacol. Res..

[352] Li X., Ma L., Fu P. (2021). The mitochondrion-targeted antioxidants in kidney disease. Curr. Med. Chem..

[353] Oliver D.M.A., Reddy P.H. (2019). Small molecules as therapeutic drugs for Alzheimer’s disease. Mol. Cell. Neurosci..

[354] Jantz-Naeem N., Guvencli N., Böttcher-Loschinski R., Böttcher M., Mougiakakos D., Kahlfuss S. (2025). Metabolic T-cell phenotypes: From bioenergetics to function. Am. J. Physiol. Cell Physiol..

[355] Official Study Title: NIRVANA: NIcotinamide Riboside in SARSCoV-2 pAtients for reNAl Protection. https://cdn.clinicaltrials.gov/large-docs/16/NCT04818216/Prot_SAP_002.pdf.

[356] Naidu A.S., Wang C.K., Rao P., Mancini F., Clemens R.A., Wirakartakusumah A., Chiu H.F., Yen C.H., Porretta S., Mathai I. (2024). Precision nutrition to reset virus-induced human metabolic reprogramming and dysregulation (HMRD) in long-COVID. NPJ Sci. Food.

[357] Park H.S., Liu G., Liu Q., Zhou Y. (2018). Swine Influenza virus induces RIPK1/DRP1-mediated interleukin-1 beta production. Viruses.

[358] Hu M., Schulze K.E., Ghildyal R., Henstridge D.C., Kolanowski J.L., New E.J., Hong Y., Hsu A.C., Hansbro P.M., Wark P.A. (2019). Respiratory syncytial virus co-opts host mitochondrial function to favour infectious virus production. Elife.

[359] Bahrampour Juybari K., Pourhanifeh M.H., Hosseinzadeh A., Hemati K., Mehrzadi S. (2020). Melatonin potentials against viral infections including COVID-19: Current evidence and new findings. Virus Res..

[360] Yen F.S., Wei J.C., Shih Y.H., Hsu C.Y., Hsu C.C., Hwu C.M. (2022). Metformin use before Influenza vaccination may lower the risks of influenza and related complications. Vaccines.

[361] Assimakopoulos S.F., Aretha D., Komninos D., Dimitropoulou D., Lagadinou M., Leonidou L., Oikonomou I., Mouzaki A., Marangos M. (2021). N-acetyl-cysteine reduces the risk for mechanical ventilation and mortality in patients with COVID-19 pneumonia: A two-center retrospective cohort study. Infect. Dis..

[362] Ibrahim H., Perl A., Smith D., Lewis T., Kon Z., Goldenberg R., Yarta K., Staniloae C., Williams M. (2020). Therapeutic blockade of inflammation in severe COVID-19 infection with intravenous N-acetylcysteine. Clin. Immunol..

[363] Geiler J., Michaelis M., Naczk P., Leutz A., Langer K., Doerr H.W., Cinatl J. (2010). N-acetyl-L-cysteine (NAC) inhibits virus replication and expression of pro-inflammatory molecules in A549 cells infected with highly pathogenic H5N1 influenza A virus. Biochem. Pharmacol..

[364] De Flora S., Grassi C., Carati L. (1997). Attenuation of influenza-like symptomatology and improvement of cell-mediated immunity with long-term N-acetylcysteine treatment. Eur. Respir. J..

[365] Wei Y.Y., Ye J.J., Zhang D.W., Hu L., Wu H.M., Fei G.H. (2024). Melatonin rescues Influenza A virus-induced cellular energy exhaustion via OMA1-OPA1-S in acute exacerbation of COPD. J. Pineal Res..

[366] Xu M.M., Kang J.Y., Wang Q.Y., Zuo X., Tan Y.Y., Wei Y.Y., Zhang D.W., Zhang L., Wu H.M., Fei G.H. (2024). Melatonin improves influenza virus infection-induced acute exacerbation of COPD by suppressing macrophage M1 polarization and apoptosis. Respir. Res..

[367] Huang Y., Jiang C., Liu X., Tang W., Gui H., Sun T., Xu D., He M., Han M., Qiu H. (2024). Melatonin suppresses TLR4-mediated RSV infection in the central nervous cells by inhibiting NLRP3 inflammasome formation and autophagy. J. Cell. Mol. Med..

[368] Qin J., Wang G., Han D. (2025). Benefits of melatonin on mortality in severe-to-critical COVID-19 patients: A systematic review and meta-analysis of randomized controlled trials. Clinics.

[369] Kakad U.U., Khopkar-Kale P.S., Tripathy S.P., Bhawalkar J.S. (2025). Potential of melatonin as a treatment option for long COVID: A call for research. Br. J. Clin. Pharmacol..

[370] Tirkan A., Eskandari D., Roham M., Aloosh O., Ramim T., Afshar H. (2024). Investigating the effectiveness of melatonin in the treatment of critically ill patients with COVID-19 hospitalized in the Intensive Care Unit: A double-blind randomized clinical trial. Med. J. Islam. Repub. Iran.

[371] Bahmyari R., Zare M., Sharma R., Agarwal A., Halvaei I. (2020). The efficacy of antioxidants in sperm parameters and production of reactive oxygen species levels during the freeze-thaw process: A systematic review and meta-analysis. Andrologia.

[372] Maio N., Cherry S., Schultz D.C., Hurst B.L., Linehan W.M., Rouault T.A. (2022). TEMPOL inhibits SARS-CoV-2 replication and development of lung disease in the Syrian hamster model. iScience.

[373] Jin Z., Du X., Xu Y., Deng Y., Liu M., Zhao Y., Zhang B., Li X., Zhang L., Peng C. (2020). Structure of M^pro^ from SARS-CoV-2 and discovery of its inhibitors. Nature.

[374] Gouédard C., Barouki R., Morel Y. (2004). Dietary polyphenols increase paraoxonase 1 gene expression by an aryl hydrocarbon receptor-dependent mechanism. Mol. Cell. Biol..

[375] Camps J., Marsillach J., Joven J. (2009). Pharmacological and lifestyle factors modulating serum paraoxonase-1 activity. Mini Rev. Med. Chem..

[376] Mellor A.L., Munn D.H. (2004). IDO expression by dendritic cells: Tolerance and tryptophan catabolism. Nat. Rev. Immunol..

[377] Rothhammer V., Quintana F.J. (2019). The aryl hydrocarbon receptor: An environmental sensor integrating immune responses in health and disease. Nat. Rev. Immunol..

[378] Cicin I., Plimack E.R., Gurney H., Leibowitz R., Alekseev B.Y., Parnis F.X., Peer A., Necchi A., Bellmunt J., Nishiyam H. (2024). Epacadostat plus pembrolizumab versus placebo plus pembrolizumab for advanced urothelial carcinoma: Results from the randomized phase III ECHO-303/KEYNOTE-698 study. BMC Cancer.

[379] Lara P.N., Villanueva L., Ibanez C., Erman M., Lee J.L., Heinrich D., Lipatov O.N., Gedye C., Gokmen E., Acevedo A. (2024). A randomized, open-label, phase 3 trial of pembrolizumab plus epacadostat versus sunitinib or pazopanib as first-line treatment for metastatic renal cell carcinoma (KEYNOTE-679/ECHO-302). BMC Cancer.

[380] Chen F., Zhao D., Huang Y., Wen X., Feng S. (2023). Synergetic impact of combined navoximod with cisplatin mitigates chemo-immune resistance via blockading IDO1+ CAFs-secreted Kyn/AhR/IL-6 and pol ζ-prevented CIN in human oral squamous cell carcinoma. Life Sci..

[381] Guillemin G.J., Brew B.J. (2002). Implications of the kynurenine pathway and quinolinic acid in Alzheimer’s disease. Redox Rep..

[382] Låg M., Skuland T., Ballangby J., Grytting V.S., Jørgensen R.B., Snilsberg B., Øvrevik J., Holme J.A., Refsnes M. (2025). Mechanisms involved in pro-inflammatory responses to traffic-derived particulate matter (PM) in THP-1 macrophages compared to HBEC3-KT bronchial epithelial cells. Toxicology.

[383] Bogan K.L., Brenner C. (2008). Nicotinic acid, nicotinamide, and nicotinamide riboside: A molecular evaluation of NAD+ precursor vitamins in human nutrition. Annu. Rev. Nutr..

[384] Agus A., Planchais J., Sokol H. (2018). Gut microbiota regulation of tryptophan metabolism in health and disease. Cell Host Microbe.

[385] Overmyer K.A., Shishkova E., Miller I.J., Balnis J., Bernstein M.N., Peters-Clarke T.M., Meyer J.G., Quan Q., Muehlbauer L.K., Trujillo E.A. (2021). Large-scale multi-omic analysis of COVID-19 severity. Cell Syst..

[386] Grimes J.M., Khan S., Badeaux M., Rao R.M., Rowlinson S.W., Carvajal R.D. (2021). Arginine depletion as a therapeutic approach for patients with COVID-19. Int. J. Infect. Dis..

[387] Greene K.S., Choi A., Chen M., Yang N., Li R., Qiu Y., Lukey M.J., Rojas K.S., Shen J., Wilson K.F. (2023). Inhibiting glutamine metabolism blocks coronavirus replication in mammalian cells. bioRxiv.

[388] Rodríguez-Vera D., Salazar J.R., Soriano-Ursúa M.A., Guzmán-Pérez J., Vergara-Castañeda A., Muñoz-Durán H., Ramírez-Velez G.L., Vivar-Sierra A., Naranjo-Navarro C.R., Meza-Meneses P.A. (2024). Effectiveness of omega-3 fatty acid supplementation in improving the metabolic and inflammatory profiles of Mexican adults hospitalized with COVID-19. Diseases.

[389] Safaei Ardestani S.Z., Rahideh S.T. (2022). The effect of omega-3 fatty acid supplementation on clinical and biochemical parameters of critically ill patients with COVID-19: A randomized clinical trial. J. Transl. Med..

[390] Wang K., Khoramjoo M., Srinivasan K., Gordon P.M.K., Mandal R., Jackson D., Sligl W., Grant M.B., Penninger J.M., Borchers C.H. (2023). Sequential multi-omics analysis identifies clinical phenotypes and predictive biomarkers for long COVID. Cell Rep. Med..

[391] Pinero S., Li X., Liu L., Li J., Lee S.H., Winter M., Nguyen T., Zhang J., Le T.D. (2025). Integrative multi-omics framework for causal gene discovery in long COVID. medRxiv.

[392] Centers for Disease Control and Prevention Influenza Severity Assessment and Estimated Influenza Illnesses, Medical Visits, Hospitalizations, and Deaths That Occurred and Those That Were Prevented by Vaccination in the United States–2023–2024 Influenza Season. https://www.cdc.gov/flu/whats-new/flu-summary-addendum-2023-2024.html.

[393] Havers F.P., Whitaker M., Melgar M., Chatwani B., Chai S.J., Alden N.B., Meek J., Openo K.P., Ryan P.A., Kim S. (2023). Characteristics and outcomes among adults aged ≥60 years hospitalized with laboratory-confirmed respiratory syncytial virus-RSV-NET, 12 States, July 2022–June 2023. MMWR Morb. Mortal. Wkly. Rep..

[394] Makowski L., Chaib M., Rathmell J.C. (2020). Immunometabolism: From basic mechanisms to translation. Immunol. Rev..

[395] Darweesh M., Mohammadi S., Rahmati M., Al-Hamadani M., Al-Harrasi A. (2025). Metabolic reprogramming in viral infections: The interplay of glucose metabolism and immune responses. Front. Immunol..

[396] Teer E., Mukonowenzou N.C., Essop M.F. (2025). HIV, inflammation, and immunometabolism: A model of the inflammatory theory of disease. Viruses.

[397] Lee C.H., Banoei M.M., Ansari M., Cheng M.P., Lamontagne F., Griesdale D., Lasry D.E., Demir K., Dhingra V., Tran K.C. (2024). Using a targeted metabolomics approach to explore differences in ARDS associated with COVID-19 compared to ARDS caused by H1N1 influenza and bacterial pneumonia. Crit. Care..

[398] Lin Z., Xue M., Lu M., Liu S., Jiang Y., Yang Q., Cui H., Huang X., Zheng Z., Sun B. (2025). Multi-omics driven biomarker discovery and pathological insights into Pseudomonas aeruginosa pneumonia. BMC Infect. Dis..

[399] Ghini V., Pecchioli V., Celli T., Boccia N., Bertini L., Veneziani F., Vannucchi V., Turano P. (2025). Metabolomic and lipoproteomic differences and similarities between COVID-19 and other types of pneumonia. Sci. Rep..

[400] Meoni G., Lorini S., Monti M., Madia F., Corti G., Luchinat C., Zignego A.L., Tenori L., Gragnani L. (2019). The metabolic fingerprints of HCV and HBV infections studied by Nuclear Magnetic Resonance Spectroscopy. Sci. Rep..

